# Regioselective Oxidative Arylation of Fluorophenols

**DOI:** 10.1002/anie.201910352

**Published:** 2019-10-31

**Authors:** Congjun Yu, Frederic W. Patureau

**Affiliations:** ^1^ Institute of Organic Chemistry, RWTH Aachen University Landoltweg 1 52074 Aachen Germany

**Keywords:** arylated quinone, cross dehydrogenative coupling, fluorophenols, hypervalent iodine, regioselective arylation

## Abstract

A metal free and highly regioselective oxidative arylation reaction of fluorophenols is described. The relative position of the fluoride leaving group (i.e., *ortho* or *para*) controls the 1,2 or 1,4 nature of the arylated quinone product, lending versatility and generality to this oxidative, defluorinative, arylation concept.

## Graphical abstract

**F trick**: A metal free and highly regioselective oxidative arylation reaction of fluorophenols is described.

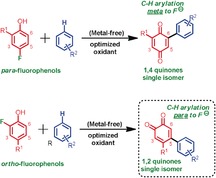

The quinone motif is ubiquitous in chemistry, material science, nanotechnology, and medicine.^1^ Due to their electron and hydrogen transport properties, quinone derivatives participate in various biological activities.^2^ For example, they act as membrane‐bound compounds found in many living organism,^3^ and play a key role in cell respiration.^4^ In addition, they are embedded in many natural products, drugs,^5^ and materials.^6^ Among them, arylated quinones (Figure 1) are moreover particularly important. They possess unique photochemical and electronic properties with applications in photosynthesis^7^ photocatalysis and the dye industry.^8^ Thus, the efficient synthesis of aryl substituted quinones remains a strategic objective, especially in terms of arylation regioselectivity, a largely unaddressed challenge.

**Figure 1:**
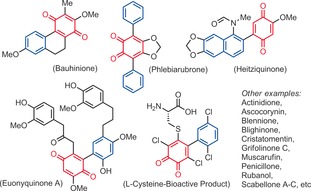
Natural products containing arylated quinones.

Some established methods to synthesize arylated quinones include pre‐halogenation of the quinone core followed by palladium‐catalyzed cross‐coupling,^9^ or utilizing diazonium salts as aryl radical source to functionalize the quinone.^10^ The latter methods however are neither step nor atom economical, and typically rely on sensitive/onerous palladium/phosphine catalysts. In 2011, Baran et al.^11^ reported boronic acids to be ideal radical sources to react with quinones in the presence of catalytic amounts of AgNO_3_. Since then, several radical sources and catalysts were developed to achieve this reaction.^12^ Nevertheless, these methods are typically not regioselective. The presence of a single R^1^ functional group at the quinone core generates three inequivalent but similarly reactive electrophilic positions, usually leading to regioisomeric mixtures of diversely arylated coupling products. Cross dehydrogenative coupling (CDC) approaches were also developed with success,^13^ for example by You and co‐workers. Nevertheless, these modern methods, while highly step and atom economical, do not solve the regioselectivity problem at the quinone. Herein, we propose to solve this problem for the 1,4 and the more challenging 1,2 quinones (Scheme 1).

**Scheme 1:**
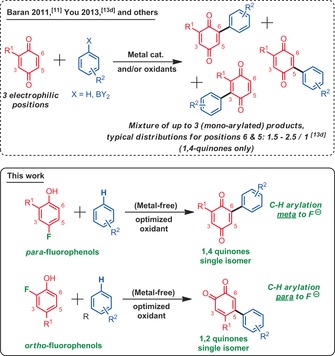
Synthesis of arylated quinones.

Since a few years, our research group has been focused on the development of new oxidant mediated CDCs.^14^ In this context, the nature of the oxidant is particularly important because of its specific interactions with the substrates (and catalysts). Therefore, tuning its design can alter reactivity, and thus, lead to new reaction concepts. Along this strategy, we were attempting to develop a cross dehydrogenative arylation method of phenols with notably hypervalent iodine oxidants, when we serendipitously came across the very regioselective arylated quinone **1 c** depicted in Scheme 2. We then optimized the reaction conditions. It rapidly transpired that the reaction only operates with fluorophenols, wherein the fluoride acts as a leaving group. Moreover, the *ipso* arylation event does not occur. Instead, the arylation consistently occurs in *meta* position with respect to the fluoride leaving group (in the case of *para*‐fluorophenols starting materials, see beneath for *ortho*‐fluorophenols). Importantly, the arylation product is not observed when the fluoride leaving group is replaced by chloride, bromide, or nitro. Furthermore, neither quinones nor hydroquinones deliver any arylation products, indicating that the latter structures are not reaction intermediates. In other words, the remote fluoride anion leaving group plays an essential enabling role in this arylation reaction.

**Scheme 2:**
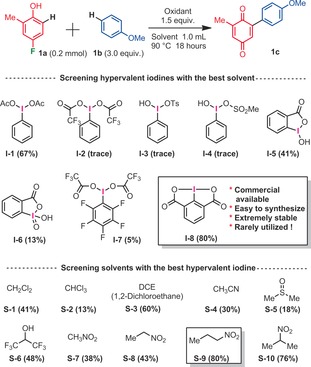
Reaction optimization, yields determined by ^1^H NMR spectroscopy of the crude reaction mixture with 1,3,5‐trimethoxybenzene as an internal standard.

Different hypervalent iodine oxidants^15^ were tested in this reaction. Iodosodilactone (**I‐8**), a rare but trivial (non‐fluorinated) hypervalent iodine reagent, performed best (80 % yield of test coupling product **1 c**). Iodosodilactone **I‐8** was firstly synthesized and reported by Agosta^16^ in 1965. However, almost no reactions enabled by this iodine reagent were reported since then. In 2012, the Zhang group^17^ reported the single crystal structure of iodosodilactone **I‐8**, showing an unusual planar shape which is unlike other typical aryl‐λ^3^‐iodanes (i.e., PIDA), with a typical T‐shape structure. In any case, iodosodilactone **I‐8** can be prepared in large amounts with high yield. Iodosodilactone **I‐8** is quite stable, it is neither air nor moisture sensitive, and can be stored for several months at room temperature without any detectable decomposition. Interestingly, the solvent also has a large impact on the reaction outcome. 1‐Nitropropane, a cheap but also relatively rarely utilized solvent, outperforms other nitroalkanes and diverse usual organic solvents such as 1,2‐dichloroethane (DCE).

With the best conditions in hand, we then explored the scope of this metal‐free arylation reaction, for the synthesis of a variety of arylated 1,4‐quniones (Scheme 3). For all the examples in which a *para*‐fluorophenol was utilized as one of the building blocks, the arylation occurred exclusively at the 6‐C−H‐position. No 5‐C−H or *ipso*‐(4‐C−F)‐arylations were detected. Moreover, electron rich arylation coupling partners were found to be important. Indeed, anisoles react particularly well. It should be noted that the C−H regioselectivity on the side of the anisole coupling partner is usually high, even if in some cases some minor (*o/m/p*) isomers could be detected.

**Scheme 3:**
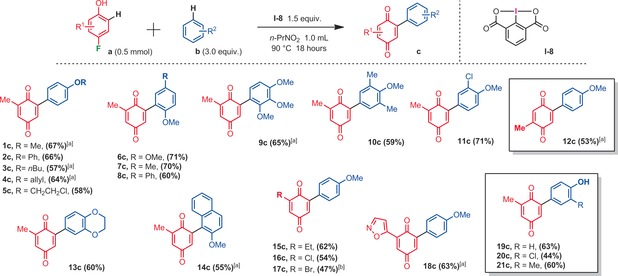
Regioselective synthesis of arylated 1,4‐quinones, isolated yields. [a] Trace amounts of different selectivities (*o/m/p*) at the anisole moiety. [b] Iodosobenzene diacetate (PIDA, **I‐1**) was utilized instead of iodosodilactone.

Some interesting functional groups were well tolerated, such as halides (**5 c, 11 c, 16 c, 20 c**), an allyl ether (**4 c**), an isoxazole heterocycle (**18 c**), and even phenols (**19**–**21 c**). In the latter cases, very important phenolic quinones could be constructed in a single step, which would be difficult to synthetize otherwise (for the relevance of such phenolic quinone structures, see for example Euonyquinone A, Figure 1). In addition to arylated 1,4‐quinone products, arylated 1,2‐quinones could be accessed from *ortho*‐fluorophenol substrates (Scheme 4). 1,2‐Quinones are also ubiquitous in many natural products, drugs and materials.^18^ However, the regioselective arylation of 1,2‐quinones is very challenging.^19^ Interestingly, in this configuration, the *ortho*‐fluorophenol building block must also carry a *para*‐methoxy electron donating functional group. Moreover, the arylation regioselectivity has now changed from *meta* to *para* C−H arylation with respect to the fluoride leaving group, delivering 4,5‐disubstituted‐1,2‐quinones. While the isolated yields remain moderate (**1 e**–**9 e**), a large variety of anisole arylation coupling partners were well tolerated. In spite of the mentioned limitations above, the operational simplicity of this approach may pave the way for future arylation methods of 1,2‐quinones. Finally, we also explored and optimized a diarylation method derived from these reaction conditions, with a single fluoride leaving group.^20^ Re‐optimizing the hypervalent iodine oxidant (PIFA, **I‐2**) as well as the reaction temperature (−20 °C) afforded 2,6‐diarylated‐1,4‐quinones in promising yields (Scheme 5).

**Scheme 4:**
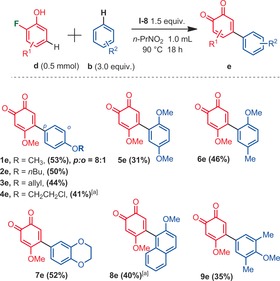
Regioselective synthesis of arylated 1,2‐quinones, isolated yields. [a] Trace amounts of different selectivities (*o/m/p*) at the anisole moiety.

**Scheme 5:**
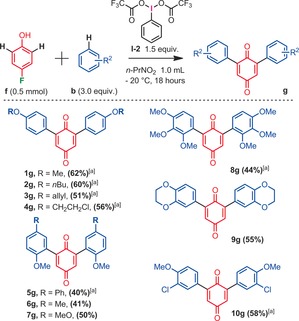
Regioselective synthesis of diarylated 1,4‐quinones, isolated yields. [a] Trace amounts of different selectivities (*o/m/p*) at the anisole moiety.

Experiments to elucidate the mechanistic pathway were then performed. In situ ^19^F NMR spectroscopy in a polytetrafluoroethylene (PTFE) tube was carried out. As oxidant **I‐1** (PIDA) was utilized under otherwise standard conditions, and the ^19^F NMR spectra were measured every 30 minutes after the reaction started. The rapid appearance of a characteristic quartet was observed increasing with time at around *δ*=+49 ppm (q, ^3^ *J*_F‐H_=7 Hz), calibrated in relation to benzotrifluoride (−63 ppm),^21^ which corresponds to the buildup of acetyl fluoride (AcF, see Supporting Information).^22^

This result indicates the role and fate of the fluoride leaving group. Based on this observation, we propose that the “pseudo‐ketal”^23^ intermediate **int‐II** 24, 25, 26 is formed (Scheme 6, mechanistic proposal). The fluoride anion would then attack the adjacent carboxyl ester upon the remote nucleophilic attack of the arene coupling partner (intermediates **int‐III** and **int‐IV**). This would release the oxygen atom towards the hydroquinone intermediate **int‐V**, which would further oxidize under aerobic conditions to the final 1,4‐quinone coupling product **c**. In support of this hypothesis, we furthermore noted that, while the reaction still operates under carefully degassed condition (N_2_), the isolated yield of product **1 c** decreases to only 23 % (see Supporting Information). These two observations: 1) the presence of AcF, and 2)  the reaction still operating under N_2_, albeit in low yield, allow the conclusion that the second oxygen atom is indeed originated from the hypervalent iodine oxidant. In the case of *ortho*‐fluorophenol building blocks, the corresponding reaction intermediate **int‐V′** is in principle blocked at the quaternary carbon stage. We propose that this structure is unlocked by the 1,2‐aryl migration to the neighboring most electrophilic carbon, yielding catechol intermediate **int‐VI′/ int‐VII′**, which is oxidized to the 1,2 quinone final coupling product **e** under aerobic conditions. When *meta*‐ fluorophenol (**h**) was engaged as a substrate with either anisole or alternatively 1,4‐dimethoxybenzene, no coupling products were found. Finally, we propose the double arlyation products **g** of Scheme 5 to arise from a simple Brønsted acid catalyzed second arylation event (intermediate **int‐V′′**), followed by aerobic oxidation. This is in good agreement with the fact that the more electrophilic/acidic hypervalent iodine reagent **I‐2** performs particularly well in this double arylation approach (Scheme 5 & 6). Moreover, it should be noted that, in the absence of a second fluoride leaving group, the regioselectivity of this second arylation event is probably controlled by the steric impact of the first aryl group in combination with a very low reaction temperature (−20 °C).

**Scheme 6:**
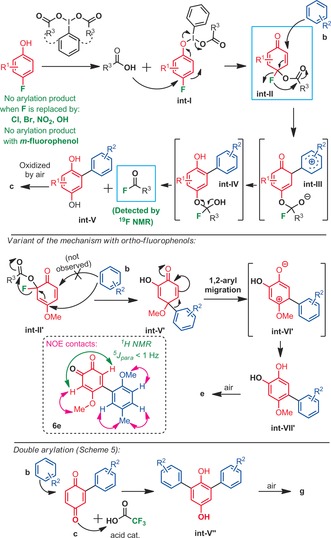
Proposed mechanistic pathways.

The versatility of this reaction was then examined with the late stage C−H “quinonation” of Etofenprox, an insecticide containing multiple ether functional groups as well as multiple C(sp)^2^−H bond candidates (Scheme 7). A single cross‐coupling product was obtained in promising (for such a large structure) 42 % yield, without further optimization.

**Scheme 7:**
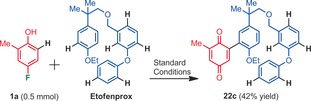
Late stage C−H “quinonation” of Etofenprox.

In summary, we have developed a method for regioselective oxidative arylation of fluorophenols. In contrast to the state of the art in this field, this method directly provides highly regioselective arylated *para* and even *ortho* quinones. The high regioselectivity is dictated by the relative position of the fluoride leaving group. We anticipate that this method will impact the field of bioactive and material based quinones.

## Conflict of interest

The authors declare no conflict of interest.

## Supporting information

As a service to our authors and readers, this journal provides supporting information supplied by the authors. Such materials are peer reviewed and may be re‐organized for online delivery, but are not copy‐edited or typeset. Technical support issues arising from supporting information (other than missing files) should be addressed to the authors.

Supplementary
