# Supplementary material for: Regioselective Oxidative Arylation of Fluorophenols
Source: Angew Chem Int Ed Engl. 2019 Oct 31;58(51):18530–4. doi: 10.1002/anie.201910352 (PMC6916641; doi:10.1002/anie.201910352)

Angewandte Chemie
Eine Zeitschrift der Gesellschaft Deutscher Chemiker

Supporting Information

Regioselective Oxidative Arylation of Fluorophenols

*Congjun Yu and Frederic W. Patureau**

anie_201910352_sm_miscellaneous_information.pdf

Table of Contents

1. General information……………………………………………………………………….2
2. General procedure for highly selective arylation of quinones………………………………..2
3. Synthesis of the hypervalent iodine reagent……………………………………...……......3
4. Product characterization…………..…………………………………….……………..….4
5. Other substrates………………………………………….....…………..……….……….21
6. Mechanistic experiments…………………………...…………………………….…..….22
7. Copies of ^{1}H, ^{13}C and NOESY Spectra…………….…………………………….…..….23

1. General information

All reactions were carried out in dried reaction vials with sealed aluminous headspace caps under air, unless otherwise specified. NMR spectra were obtained on a VNMRS 300 or VNMRS 400 or VNMRS 600 using $CDCl_3$ or DMSO-d_6 as solvents. Chemical shifts are given in ppm and coupling constants (*J*) in Hz. For spectra with $CDCl_3$ as a solvent, ^{1}H spectra were calibrated in relation to the reference measurement of TMS (0.00 ppm), ^{13}C spectra were calibrated in relation to deuterated solvent, namely $CDCl_3$ (77.16 ppm). The following abbreviations were used for ^{1}H NMR spectra to indicate the signal multiplicity: s (singlet), d (doublet), t (triplet), q (quartet) and m (multiplet) as well as combinations of them. Flash chromatography was performed on silica gel (60 M, 0.04-0.063 mm) by standard technique. Substrates were purchased either from Sigma Aldrich, ABCR, Alfa Aesar, TCI, or chemPUR. High resolution mass spectra (HRMS) were obtained on a Thermo Scientific LTQ Orbitrap XL spectrometer.

2. General procedure for highly selective arylation of quinones

(1) Procedure A

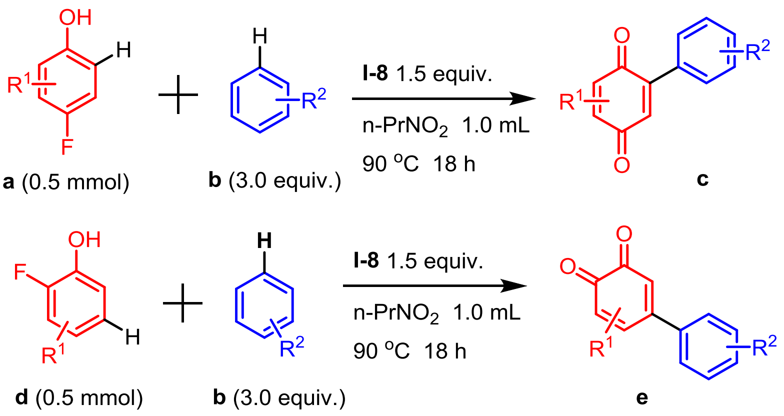

Unless otherwise specified, the phenol **a** or **d** (0.5 mmol scale) and the aryls **b** (3.0 equiv.) are added into the solvent 1-nitropropane (1.0 mL) in a 20 mL reaction vial. After that, the hypervalent iodine reagent iodosodilactone **I-8** (1.5 equiv.) is added. The reaction vial is then sealed with aluminous headspace cap with air inside and exposed to 90 °C for 18 hours. Magnetic stirring set to approx. 240 turns/min. The reactor is then cooled to room temperature. The crude is directly engaged on SiO_2 gel column chromatography for purification. The expected cross-coupling product generally appears as a colorful spot which is visible to human eyes on the TLC plate without UV light.

(2) Procedure B

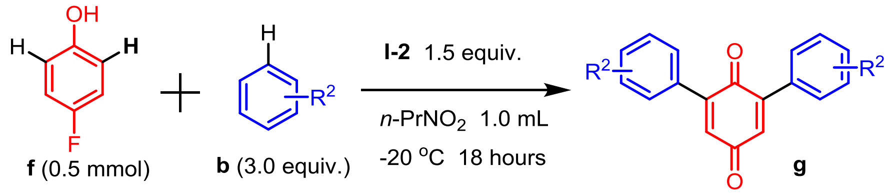

Unless otherwise specified, the phenol **f** (0.5 mmol scale) and the aryls **b** (3.0 equiv.) are added into the solvent 1-nitropropane (1.0 mL) in a 20.0 mL reaction vial. The reaction vial is then sealed with aluminous headspace cap with air inside and kept stirring for 20 minutes under -20 °C. After that, the hypervalent iodine reagent [Bis(trifluoroacetoxy)iodo]benzene **I-2** (1.5 equiv.) is added. Then keep the reaction stirring for another 18 hours. After that, the crude is directly engaged on SiO_2 gel column chromatography for purification. The expected cross-coupling product generally appears as a colorful spot which is visible to the human eye on the TLC plate without UV light.

3. Synthesis of hypervalent iodine reagent iodosodilactone (I-8)

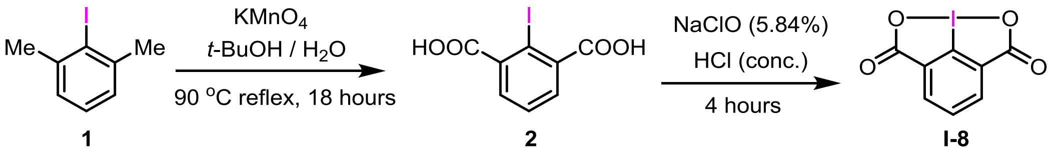

(1) synthesis of **2**

10.0 g (43.0 mmol, 1.0 equiv.) 2-iodo-1,3-dimethylbenzene **1** was dissolved into the mixed solvents *t*-BuOH (40.0 mL) and water (40.0 mL). Then 40 g (240.0 mmol, 5.6 equiv.) $KMnO_4$ was added. After refluxing for 18 hours, the reaction was stopped and the reaction liquid was filtered to get rid of the solid inside. Concentrated HCl was then added into the filtrate slowly till its pH arrived at 1. Then separated the organic layer from the aqueous layer and washed the aqueous layer by ethyl acetate. After that, saturated $NaHCO_3$ solvent was added into the combined organic liquid. The aqueous layer was separated from the organic layer and washed by ethyl acetate to get rid of organic byproducts. Concentrated HCl was again added into the collected aqueous liquid to get white solid. After filtration, white solid product **2** was obtained (6.5 g) in 52% yield.

(2) synthesis of **I-8**

5.5 g (18.9 mmol, 1.0 equiv.) **2** was dispersed in 72.0 mL concentrated HCl liquid. 144 mL NaClO solution (5.8% in water) was then added dropwise over an hour at room temperature. After that, kept stirring for another 4 hours. Then the reaction was stopped and the mixture was filtrated to get the white solid. Washed the solid by water and acetone to get the pure white product **I-8** by 5.2 g in 95% yield.

^{1}H NMR (400 MHz, DMSO-d_6) δ 8.32 (d, J = 7.4 Hz, 2H), 8.01 (t, J = 7.5 Hz, 1H).

^{13}C NMR (101 MHz, DMSO-d_6) δ 166.31 (s, C_{quat}), 133.81 (s, CH), 133.59 (s, CH), 128.95 (s, C_{quat}), 125.68 (s, C_{quat}).

IR (neat, cm^{-1}): ṽ: 3376, 3063, 2339, 2184, 2075, 2001, 1909, 1691, 1652, 1565, 1463, 1421, 1339, 1282, 1148, 1093, 1049, 907, 830, 797, 749, 724, 660.

ESI-HRMS: $[M+Na]^+$ *m/z*: calculated for $[C_8H_3IO_4Na]^+$ 312.89682, found 312.89679.

4. Product characterization

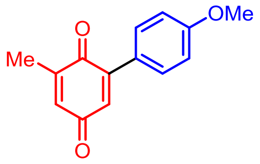

Chemical Formula: $C_{14}H_{12}O_3$

1c: Following the general procedure A, the product was converted from 4-fluoro-2-methylphenol and anisole at 90 °C for 18 hours. The crude mixture is purified by SiO_2 gel column chromatography with petane/EA (from 50/1 to 20/1). Isolated yield: 67% (dark orange solid).

^1H NMR (400 MHz, Chloroform-*d*) δ 7.47 – 7.33 (m, 2H), 7.04 – 6.83 (m, 2H), 6.68 (d, *J* = 2.5 Hz, 1H), 6.57 (dd, *J* = 2.7, 1.5 Hz, 1H), 3.77 (s, 3H), 2.05 (d, *J* = 1.6 Hz, 3H).

^{13}C NMR (101 MHz, Chloroform-*d*) δ 187.83 (s, C_{quat}), 187.59 (s, C_{quat}), 161.32 (s, C_{quat}), 146.18 (s, C_{quat}), 145.59 (s, C_{quat}), 133.31 (s, CH), 131.35 (s, CH), 130.92 (s, CH), 125.54 (s, C_{quat}), 114.13 (s, CH), 55.50 (s, CH_3), 16.47 (s, CH_3).

IR (neat, cm^{-1}): ṽ: 3867, 3300, 3023, 2924, 2843, 2556, 2287, 2160, 2032, 1903, 1748, 1646, 1597, 1506, 1458, 1376, 1305, 1281, 1245, 1177, 1118, 1079, 1026, 948, 915, 871, 830, 775, 725, 665.

ESI-HRMS: $[M+Na]^+$ *m/z*: calculated for $[C_{14}H_{12}O_3Na]^+$ 251.06787, found 251.06793.

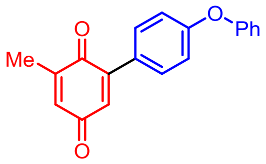

Chemical Formula: $C_{19}H_{14}O_3$

2c: Following the general procedure A, the product was converted from 4-fluoro-2-methylphenol and Diphenyl ether at 90 °C for 18 hours. The crude mixture is purified by SiO_2 gel column chromatography with petane/EA (from 50/1 to 30/1). Isolated yield: 66% (gold solid).

^1H NMR (400 MHz, Chloroform-*d*) δ 7.42 – 7.35 (m, 2H), 7.34 – 7.27 (m, 2H), 7.14 – 7.06 (m, 1H), 7.03 – 6.93 (m, 4H), 6.70 (d, *J* = 2.6 Hz, 1H), 6.62 – 6.57 (m, 1H), 2.06 (d, *J* = 1.6 Hz, 3H).

^{13}C NMR (101 MHz, Chloroform-*d*) δ 186.73 (s, C_{quat}), 186.40 (s, C_{quat}), 158.54 (s, C_{quat}), 155.20 (s, C_{quat}), 145.24 (s, C_{quat}), 144.48 (s, C_{quat}), 132.38 (s, CH), 131.01 (s, CH), 130.06 (s, CH), 129.08 (s, CH), 126.63 (s, C_{quat}), 123.29 (s, CH), 118.93 (s, CH), 117.11 (s, CH), 15.49 (s, CH_3).

IR (neat, cm^{-1}): ṽ: 3303, 3060, 2923, 2851, 2326, 2162, 2079, 2037, 1977, 1873, 1648, 1602, 1485, 1384, 1300, 1247, 1188, 1144, 1073, 1024, 976, 945, 907, 870, 802, 751, 728, 693, 666.

ESI-HRMS: $[M+Na]^+$ *m/z*: calculated for $[C_{19}H_{14}O_3Na]^+$ 313.08352, found 313.08368.

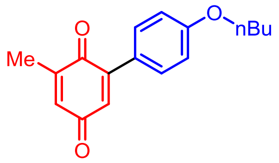

Chemical Formula: $C_{17}H_{18}O_3$

3c: Following the general procedure A, the product was converted from 4-fluoro-2-methylphenol and butoxybenzene at 90 °C for 18 hours. The crude mixture is purified by SiO_2 gel column chromatography with petane/EA (from 50/1 to 20/1). Isolated yield: 57% (dark orange solid).

^{1}H NMR (600 MHz, Chloroform-*d*) δ 7.37 (d, *J* = 8.3 Hz, 2H), 6.87 (d, *J* = 8.5 Hz, 2H), 6.68 (s, 1H), 6.58 (s, 1H), 3.93 (t, *J* = 6.5 Hz, 2H), 2.05 (s, 3H), 1.77 – 1.66 (m, 2H), 1.48 – 1.36 (m, 2H), 0.91 (t, *J* = 7.4 Hz, 3H).

^{13}C NMR (151 MHz, Chloroform-*d*) δ 187.89 (s, C_{quat}), 187.66 (s, C_{quat}), 160.96 (s, C_{quat}), 146.18 (s, C_{quat}), 145.66 (s, C_{quat}), 133.32 (s, CH), 131.23 (s, CH), 130.91 (s, CH), 125.26 (s, C_{quat}), 114.64 (s, CH), 67.94 (s, CH_2), 31.32 (s, CH_2), 19.34 (s, CH_2), 16.51 (s, CH_3), 13.97 (s, CH_3).

IR (neat, cm^{-1}): ṽ: 3323, 3045, 2956, 2872, 2554, 2159, 2022, 1976, 1653, 1603, 1509, 1468, 1376, 1247, 1180, 1121, 1073, 1027, 1006, 970, 913, 874, 833, 804, 768, 732, 686.

ESI-HRMS: $[M+Na]^+$ *m/z*: calculated for $[C_{17}H_{18}O_3Na]^+$ 293.11482, found 293.11481.

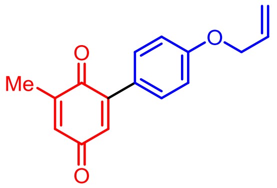

Chemical Formula: $C_{16}H_{14}O_3$

4c: Following the general procedure A, the product was converted from 4-fluoro-2-methylphenol and allyl phenyl ether at 90 °C for 18 hours. The crude mixture is purified by SiO_2 gel column chromatography with petane/EA (from 50/1 to 30/1). Isolated yield: 64% (dark orange solid).

^{1}H NMR (300 MHz, Chloroform-*d*) δ 7.49 – 7.30 (m, 2H), 6.98 – 6.82 (m, 2H), 6.68 (d, *J* = 2.6 Hz, 1H), 6.63 – 6.54 (m, 1H), 6.11 – 5.90 (m, 1H), 5.44 – 5.29 (m, 1H), 5.24 (dd, *J* = 10.3, 1.9 Hz, 1H), 4.58 – 4.48 (m, 2H), 2.05 (d, *J* = 1.6 Hz, 3H).

^{13}C NMR (75 MHz, Chloroform-*d*) δ 187.84 (s, C_{quat}), 187.60 (s, C_{quat}), 160.33 (s, C_{quat}), 146.19 (s, C_{quat}), 145.57 (s, C_{quat}), 133.33 (s, CH), 132.92 (s, CH), 131.39 (s, CH), 130.91 (s, CH), 125.66 (s, C_{quat}), 118.15 (s, CH_2), 114.89 (s, CH), 68.97 (s, CH_2), 16.49 (s, CH_3).

IR (neat, cm^{-1}): ṽ: 3270, 3046, 2923, 2866, 2551, 2162, 2030, 1975, 1898, 1650, 1599, 1608, 1456, 1423, 1376, 1300, 1238, 1179, 1118, 1079, 997, 914, 831, 725, 686.

ESI-HRMS: $[M+Na]^+$ *m/z*: calculated for $[C_{16}H_{14}O_3Na]^+$ 277.08352, found 277.08353.

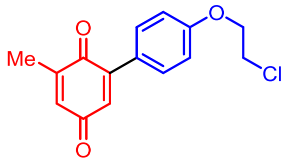

Chemical Formula: $C_{15}H_{13}ClO_3$

5c: Following the general procedure A, the product was converted from 4-fluoro-2-methylphenol and (2-chloroethoxy)benzene at 90 °C for 18 hours. The crude mixture is purified by SiO_2 gel

column chromatography with petane/EA (from 40/1 to 10/1). Isolated yield: 58% (orange solid).
^{1}H NMR (300 MHz, Chloroform-*d*) δ 7.44 – 7.35 (m, 2H), 6.95 – 6.86 (m, 2H), 6.68 (d, *J* = 2.8 Hz, 1H), 6.62 – 6.55 (m, 1H), 4.20 (t, *J* = 5.7 Hz, 2H), 3.76 (t, *J* = 5.8 Hz, 2H), 2.05 (d, *J* = 1.9 Hz, 3H).
^{13}C NMR (75 MHz, Chloroform-*d*) δ 187.77 (s, C_{quat}), 187.50 (s, C_{quat}), 159.86 (s, C_{quat}), 146.20 (s, C_{quat}), 145.45 (s, C_{quat}), 133.34 (s, CH), 131.61 (s, CH), 131.01 (s, CH), 126.25 (s, C_{quat}), 114.78 (s, CH), 68.15 (s, CH_2), 41.83 (s, CH_2), 16.49 (s, CH_3).
IR (neat, cm^{-1}): ṽ: 3260, 3058, 2966, 2921, 2868, 2747, 2512, 2325, 2161, 2079, 2027, 1920, 1766, 1647, 1600, 1507, 1448, 1421, 1378, 1279, 1243, 1180, 1116, 1079, 1036, 1003, 906, 865, 836, 808, 726, 669.
ESI-HRMS: $[M+H]^+$ *m/z*: calculated for $[C_{15}H_{14}ClO_3]^+$ 277.06260, found 277.06259.

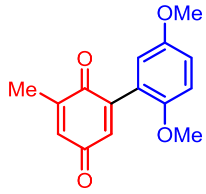

Chemical Formula: $C_{15}H_{14}O_4$

6c: Following the general procedure A, the product was converted from 4-fluoro-2-methylphenol and 1,4-dimethoxybenzene at 90 °C for 18 hours. The crude mixture is purified by SiO_2 gel column chromatography with petane/EA (from 40/1 to 10/1). Isolated yield: 71% (dark red solid).
^{1}H NMR (400 MHz, Chloroform-*d*) δ 6.92 – 6.77 (m, 2H), 6.69 – 6.63 (m, 2H), 6.60 – 6.49 (m, 1H), 3.70 (s, 3H), 3.65 (s, 3H), 2.04 (d, *J* = 1.5 Hz, 3H).
^{13}C NMR (101 MHz, Chloroform-*d*) δ 187.84 (s, C_{quat}), 186.06 (s, C_{quat}), 153.58 (s, C_{quat}), 151.41 (s, C_{quat}), 146.46 (s, C_{quat}), 146.07 (s, C_{quat}), 134.52 (s, CH), 133.16 (s, CH), 123.91 (s, C_{quat}), 116.20 (s, CH), 115.91 (s, CH), 112.63 (s, CH), 56.48 (s, CH_3), 55.93 (s, CH_3), 16.47 (s, CH_3).
IR (neat, cm^{-1}): ṽ: 3867, 3243, 3066, 3004, 2923, 2840, 2323, 2190, 2076, 1970, 1922, 1743, 1649, 1605, 1496, 1453, 1417, 1375, 1287, 1220, 1185, 1138, 1074, 1037, 903, 875, 805, 724, 668.
ESI-HRMS: $[M+Na]^+$ *m/z*: calculated for $[C_{15}H_{14}O_4Na]^+$ 281.07843, found 281.07883.

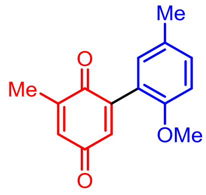

Chemical Formula: $C_{15}H_{14}O_3$

7c: Following the general procedure A, the product was converted from 4-fluoro-2-methylphenol and 1-methoxy-4-methylbenzene at 90 °C for 18 hours. The crude mixture is purified by SiO_2 gel column chromatography with petane/EA (from 50/1 to 30/1). Isolated yield: 70% (orange solid).
^{1}H NMR (400 MHz, Chloroform-*d*) δ 7.11 (dd, *J* = 8.2, 2.3 Hz, 1H), 6.88 (d, *J* = 2.3 Hz, 1H), 6.78 (d, *J* = 8.4 Hz, 1H), 6.64 (d, *J* = 2.7 Hz, 1H), 6.59 – 6.52 (m, 1H), 3.66 (s, 3H), 2.23 (s, 3H), 2.04 (d, *J* = 1.6 Hz, 3H).
^{13}C NMR (101 MHz, Chloroform-*d*) δ 187.95 (s, C_{quat}), 186.29 (s, C_{quat}), 155.16 (s, C_{quat}), 146.43 (s, C_{quat}), 146.39 (s, C_{quat}), 134.33 (s, CH), 133.13 (s, CH), 131.49 (s, CH), 131.02 (s, CH), 130.02 (s, C_{quat}), 122.94 (s, C_{quat}), 111.38 (s, CH), 55.97 (s, CH_3), 20.49 (s, CH_3), 16.47 (s, CH_3).
IR (neat, cm^{-1}): ṽ: 3263, 2924, 2845, 2544, 2332, 2037, 1886, 1818, 1758, 1645, 1602, 1496, 1458, 1383, 1297, 1253, 1179, 1149, 1076, 1026, 907, 883, 804, 729, 663.

ESI-HRMS: [M+Na]$^+$ *m/z*: calculated for $[C_{15}H_{14}O_3Na]^+$ 265.08352, found 265.08353.

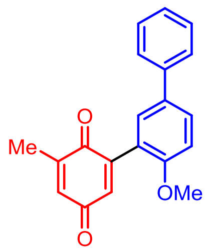

Chemical Formula: $C_{20}H_{16}O_3$

8c: Following the general procedure A, the product was converted from 4-fluoro-2-methylphenol and 4-methoxy-1,1'-biphenyl at 90 °C for 18 hours. The crude mixture is purified by SiO_2 gel column chromatography with DCM. Isolated yield: 60% (orange solid).

^{1}H NMR (400 MHz, Chloroform-*d*) δ 7.63 (dd, *J* = 8.6, 2.4 Hz, 1H), 7.58 – 7.51 (m, 2H), 7.47 – 7.37 (m, 3H), 7.36 – 7.28 (m, 1H), 7.04 (d, *J* = 8.6 Hz, 1H), 6.80 (d, *J* = 2.7 Hz, 1H), 6.67 (p, *J* = 1.6 Hz, 1H), 3.82 (s, 3H), 2.14 (d, *J* = 1.6 Hz, 3H).

^{13}C NMR (101 MHz, Chloroform-*d*) δ 187.87 (s, C_{quat}), 186.16 (s, C_{quat}), 156.73 (s, C_{quat}), 146.47 (s, C_{quat}), 146.13 (s, C_{quat}), 140.22 (s, C_{quat}), 134.63 (s, CH), 133.98 (s, C_{quat}), 133.21 (s, CH), 129.67 (s, CH), 129.31 (s, CH), 128.92 (s, CH), 127.13 (s, CH), 126.92 (s, CH), 123.49 (s, C_{quat}), 111.71 (s, CH), 56.03 (s, CH_3), 16.51 (s, CH_3).

IR (neat, cm^{-1}): ṽ: 3460, 3010, 2967, 2844, 2291, 2158, 2096, 1990, 1943, 1739, 1649, 1609, 1481, 1366, 1265, 1213, 1076, 1019, 911, 815, 765, 695.

ESI-HRMS: [M+Na]$^+$ *m/z*: calculated for $[C_{20}H_{16}O_3Na]^+$ 327.09917, found 327.09836.

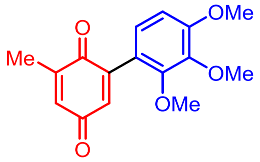

Chemical Formula: $C_{16}H_{16}O_5$

9c: Following the general procedure A, the product was converted from 4-fluoro-2-methylphenol and 1,2,3-trimethoxybenzene at 90 °C for 18 hours. The crude mixture is purified by SiO_2 gel column chromatography with petane/EA (from 40/1 to 5/1). Isolated yield: 65% (orange solid).

^{1}H NMR (400 MHz, Chloroform-*d*) δ 6.80 (d, *J* = 8.6 Hz, 1H), 6.66 – 6.60 (m, 2H), 6.59 – 6.52 (m, 1H), 3.82 (s, 3H), 3.80 (s, 3H), 3.75 (s, 3H), 2.05 (d, *J* = 1.6 Hz, 3H).

^{13}C NMR (101 MHz, Chloroform-*d*) δ 187.79 (s, C_{quat}), 186.68 (s, C_{quat}), 155.45 (s, C_{quat}), 151.92 (s, C_{quat}), 146.39 (s, C_{quat}), 146.14 (s, C_{quat}), 142.12 (s, C_{quat}), 133.83 (s, CH), 133.25 (s, CH), 124.81 (s, CH), 120.76 (s, C_{quat}), 107.13 (s, CH), 61.21 (s, CH_3), 60.84 (s, CH_3), 56.19 (s, CH_3), 16.49 (s, CH_3).

IR (neat, cm^{-1}): ṽ: 3273, 2996, 2928, 2849, 2562, 2337, 2162, 2017, 1892, 1650, 1599, 1493, 1461, 1414, 1359, 1272, 1235, 1199, 1086, 1037, 1004, 912, 811, 772, 680.

ESI-HRMS: [M+Na]$^+$ *m/z*: calculated for $[C_{16}H_{16}O_5Na]^+$ 311.08899, found 311.08902.

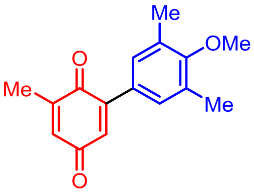

Chemical Formula: $C_{16}H_{16}O_3$

10c: Following the general procedure A, the product was converted from 4-fluoro-2-methylphenol and 2-methoxy-1,3-dimethylbenzene at 90 °C for 18 hours. The crude mixture is purified by SiO_2 gel column chromatography with DCM. Isolated yield: 59% (gold solid).

^{1}H NMR (600 MHz, Chloroform-*d*) δ 7.13 (s, 2H), 6.74 (d, *J* = 2.7 Hz, 1H), 6.65 (d, *J* = 2.6 Hz, 1H), 3.75 (s, 3H), 2.32 (s, 6H), 2.12 (s, 3H).

^{13}C NMR (151 MHz, Chloroform-*d*) δ 187.67 (s, C_{quat}), 187.39 (s, C_{quat}), 158.71 (s, C_{quat}), 146.07 (s, C_{quat}), 145.89 (s, C_{quat}), 133.16 (s, CH), 132.00 (s, CH), 131.14 (s, C_{quat}), 129.84 (s, CH), 128.52 (s, C_{quat}), 59.70 (s, CH_3), 16.32 (s, CH_3), 16.19 (s, CH_3).

IR (neat, cm^{-1}): ṽ: 3307, 2927, 2733, 2576, 2324, 2187, 2021, 1651, 1599, 1483, 1441, 1376, 1287, 1233, 1161, 1096, 1063, 1007, 912, 885, 801, 777, 753, 669.

ESI-HRMS: $[M+Na]^+$ *m/z*: calculated for $[C_{16}H_{16}O_3Na]^+$ 279.09917, found 279.09918.

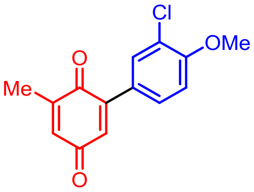

Chemical Formula: $C_{14}H_{11}ClO_3$

11c: Following the general procedure A, the product was converted from 4-fluoro-2-methylphenol and 1-chloro-2-methoxybenzene at 90 °C for 18 hours. The crude mixture is purified by SiO_2 gel column chromatography with petane/EA (from 40/1 to 10/1). Isolated yield: 71% (yellow solid).

^{1}H NMR (600 MHz, Chloroform-*d*) δ 7.48 (d, *J* = 2.6 Hz, 1H), 7.33 (dd, *J* = 8.4, 2.6 Hz, 1H), 6.91 (d, *J* = 8.3 Hz, 1H), 6.68 (d, *J* = 2.7 Hz, 1H), 6.62 – 6.55 (m, 1H), 3.88 (s, 3H), 2.06 (s, 3H).

^{13}C NMR (151 MHz, Chloroform-*d*) δ 187.52 (s, C_{quat}), 187.16 (s, C_{quat}), 156.56 (s, C_{quat}), 146.24 (s, C_{quat}), 144.42 (s, C_{quat}), 133.41 (s, CH), 131.98 (s, CH), 131.21 (s, CH), 129.14 (s, CH), 126.27 (s, C_{quat}), 122.85 (s, C_{quat}), 111.89 (s, CH), 56.41 (s, CH_3), 16.48 (s, CH_3).

IR (neat, cm^{-1}): ṽ: 3868, 3301, 3076, 2925, 2845, 2523, 2324, 2167, 2075, 2036, 1984, 1920, 1648, 1596, 1499, 1436, 1375, 1281, 1185, 1154, 1084, 1062, 1013, 928, 904, 817, 724, 697.

ESI-HRMS: $[M+Na]^+$ *m/z*: calculated for $[C_{14}H_{11}ClO_3Na]^+$ 285.02889, found 285.02917.

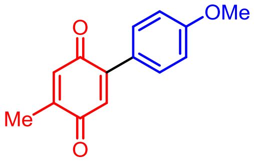

Chemical Formula: $C_{14}H_{12}O_3$

12c: Following the general procedure A, the product was converted from 4-fluoro-3-methylphenol and anisole at 90 °C for 18 hours. The crude mixture is purified by SiO_2 gel column chromatography with DCM. Isolated yield: 53% (orange solid).

^1H NMR (600 MHz, Chloroform-*d*) δ 7.48 (d, *J* = 9.1 Hz, 2H), 6.96 (d, *J* = 8.4 Hz, 2H), 6.81 (s, 1H), 6.68 (d, *J* = 2.1 Hz, 1H), 3.85 (s, 3H), 2.09 (s, 3H).

^{13}C NMR (151 MHz, Chloroform-*d*) δ 188.16 (s, C_{quat}), 187.36 (s, C_{quat}), 161.26 (s, C_{quat}), 145.61 (s, C_{quat}), 145.11 (s, C_{quat}), 133.77 (s, CH), 131.14 (s, CH), 130.88 (s, CH), 125.02 (s, C_{quat}), 114.06 (s, CH), 55.38 (s, CH_3), 15.44 (s, CH_3).

IR (neat, cm^{-1}): ṽ: 3878, 3455, 3255, 3106, 3034, 2922, 2850, 2650, 2494, 2313, 2183, 2101, 1892, 1832, 1743, 1640, 1594, 1500, 1441, 1350, 1295, 1237, 1172, 1114, 1024, 926, 828, 685.

ESI-HRMS: $[M+Na]^+$ *m/z*: calculated for $[C_{14}H_{12}O_3Na]^+$ 251.06787 found 251.06702.

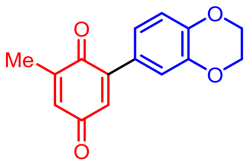

Chemical Formula: $C_{15}H_{12}O_4$

13c: Following the general procedure A, the product was converted from 4-fluoro-2-methylphenol and benzo-1,4-dioxane at 90 °C for 18 hours. The crude mixture is purified by SiO_2 gel column chromatography with petane/EA (from 40/1 to 10/1). Isolated yield: 60% (orange solid).

^1H NMR (600 MHz, Chloroform-*d*) δ 6.98 (d, *J* = 3.0 Hz, 1H), 6.92 (dd, *J* = 8.3, 2.5 Hz, 1H), 6.84 (d, *J* = 8.3 Hz, 1H), 6.66 (d, *J* = 2.9 Hz, 1H), 6.59 – 6.54 (m, 1H), 4.26 – 4.18 (m, 4H), 2.05 (s, 3H).

^{13}C NMR (151 MHz, Chloroform-*d*) δ 187.78 (s, C_{quat}), 187.37 (s, C_{quat}), 146.23 (s, C_{quat}), 145.59 (s, C_{quat}), 145.45 (s, C_{quat}), 143.55 (s, C_{quat}), 133.28 (s, CH), 131.70 (s, CH), 126.41 (s, C_{quat}), 122.88 (s, CH), 118.55 (s, CH), 117.53 (s, CH), 64.70 (s, CH_2), 64.36 (s, CH_2), 16.49 (s, CH_3).

IR (neat, cm^{-1}): ṽ: 3845, 3300, 3152, 3001, 2952, 2724, 2638, 2558, 2264, 2190, 2162, 2077, 2020, 1971, 1917, 1871, 1740, 1649, 1613, 1575, 1504, 1451, 1425, 1363, 1318, 1276, 1186, 1129, 1063, 913, 881, 804, 740, 664.

ESI-HRMS: $[M+Na]^+$ *m/z*: calculated for $[C_{15}H_{12}O_4Na]^+$ 279.06278, found 279.06302.

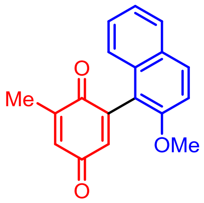

Chemical Formula: $C_{18}H_{14}O_3$

14c: Following the general procedure A, the product was converted from 4-fluoro-2-methylphenol and 2-methoxynaphthalene at 90 °C for 18 hours. The crude mixture is purified by SiO_2 gel column chromatography with DCM. Isolated yield: 55% (maroon solid).

^1H NMR (600 MHz, Chloroform-*d*) δ 7.93 (d, *J* = 9.3 Hz, 1H), 7.83 (d, *J* = 8.1 Hz, 1H), 7.56 (d, *J* = 8.5 Hz, 1H), 7.47 – 7.40 (m, 1H), 7.36 (t, *J* = 7.5 Hz, 1H), 7.33 (d, *J* = 9.3 Hz, 1H), 6.78 (d, *J* = 2.7 Hz, 1H), 6.75 (d, *J* = 2.5 Hz, 1H), 3.87 (s, 3H), 2.16 (s, 3H).

^{13}C NMR (151 MHz, Chloroform-*d*) δ 187.73 (s, C_{quat}), 186.39 (s, C_{quat}), 154.45 (s, C_{quat}), 146.71 (s, C_{quat}), 145.04 (s, C_{quat}), 136.97 (s, CH), 133.49 (s, CH), 132.50 (s, C_{quat}), 131.20 (s, CH), 129.06 (s, C_{quat}), 128.49 (s, CH), 127.32 (s, CH), 123.98 (s, CH), 123.78 (s, CH), 116.72 (s, C_{quat}), 113.14 (s, CH), 56.73 (s, CH_3), 16.56 (s, CH_3).

IR (neat, cm^{-1}): ṽ: 3847, 3269, 3055, 3008, 2924, 2844, 2514, 2324, 2188, 2158, 2072, 2038, 2016, 1978, 1921, 1744, 1651, 1596, 1509, 1469, 1433, 1378, 1339, 1259, 1188, 1147, 1125, 1073, 1024, 993, 928, 909, 860, 813, 742, 704, 676.
ESI-HRMS: $[M+Na]^+$ *m/z*: calculated for $[C_{18}H_{14}O_3Na]^+$ 301.08352, found 301.08347.

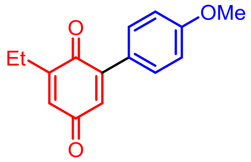

Chemical Formula: $C_{15}H_{14}O_3$

15c: Following the general procedure A, the product was converted from 4-fluoro-2-ethylphenol and anisole at 90 °C for 18 hours. The crude mixture is purified by SiO_2 gel column chromatography with DCM. Isolated yield: 62% (orange solid).
^{1}H NMR (600 MHz, Chloroform-*d*) δ 7.45 (d, *J* = 8.4 Hz, 2H), 6.95 (d, *J* = 8.9 Hz, 2H), 6.75 (d, *J* = 2.7 Hz, 1H), 6.59 (d, *J* = 2.7 Hz, 1H), 3.85 (s, 3H), 2.53 (q, *J* = 7.4 Hz, 2H), 1.17 (t, *J* = 7.4 Hz, 3H).
^{13}C NMR (151 MHz, Chloroform-*d*) δ 188.10 (s, C_{quat}), 187.34 (s, C_{quat}), 161.31 (s, C_{quat}), 151.18 (s, C_{quat}), 145.76 (s, C_{quat}), 131.60 (s, CH), 131.17 (s, CH), 130.95 (s, CH), 125.61 (s, C_{quat}), 114.14 (s, CH), 55.51 (s, CH_3), 22.67 (s, CH_2), 11.92 (s, CH_3).
IR (neat, cm^{-1}): ṽ: 3847, 3233, 2968, 2922, 2838, 2514, 2296, 2171, 2075, 2031, 1980, 1873, 1779, 1645, 1597, 1509, 1460, 1419, 1384, 1313, 1284, 1241, 1177, 1119, 1087, 1049, 1017, 986, 905, 837, 808, 762, 669.
ESI-HRMS: $[M+Na]^+$ *m/z*: calculated for $[C_{15}H_{14}O_3Na]^+$ 265.08352, found 265.08356.

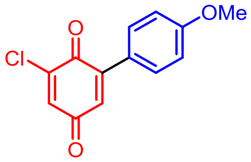

Chemical Formula: $C_{13}H_9ClO_3$

16c: Following the general procedure A, the product was converted from 2-chloro-4-fluorophenol and anisole at 90 °C for 18 hours. The crude mixture is purified by SiO_2 gel column chromatography with DCM. Isolated yield: 54% (dark red solid).
^{1}H NMR (600 MHz, Chloroform-*d*) δ 7.48 (d, *J* = 9.0 Hz, 2H), 7.04 (d, *J* = 2.8 Hz, 1H), 6.97 (d, *J* = 9.1 Hz, 2H), 6.83 (d, *J* = 2.7 Hz, 1H), 3.86 (s, 3H).
^{13}C NMR (151 MHz, Chloroform-*d*) δ 185.18 (s, C_{quat}), 179.52 (s, C_{quat}), 161.82 (s, C_{quat}), 145.57 (s, C_{quat}), 144.39 (s, C_{quat}), 133.64 (s, CH), 131.33 (s, CH), 131.03 (s, CH), 124.86 (s, C_{quat}), 114.36 (s, CH), 55.58 (s, CH_3).
IR (neat, cm^{-1}): ṽ: 3851, 3331, 3269, 3052, 3020, 2924, 2848, 2544, 2323, 2190, 2138, 2038, 1993, 1886, 1740, 1672, 1638, 1597, 1506, 1458, 1418, 1292, 1242, 1181, 1128, 999, 910, 879, 837, 811, 751, 660.
ESI-HRMS: $[M+H]^+$ *m/z*: calculated for $[C_{13}H_{10}ClO_3]^+$ 249.03130, found 249.03111.

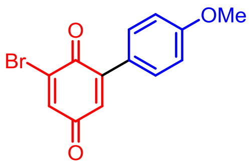

Chemical Formula: $C_{13}H_9BrO_3$

17c: Following the general procedure A, the product was converted from 2-bromo-4-fluorophenol and anisole at 90 °C for 18 hours. The crude mixture is purified by SiO_2 gel column chromatography with DCM. Isolated yield: 47% (brown solid).

^{1}H NMR (600 MHz, Chloroform-*d*) δ 7.40 (d, *J* = 9.0 Hz, 2H), 7.27 – 7.23 (m, 1H), 6.89 (d, *J* = 9.0 Hz, 2H), 6.76 (d, *J* = 2.7 Hz, 1H), 3.79 (s, 3H).

^{13}C NMR (151 MHz, Chloroform-*d*) δ 184.98 (s, C_{quat}), 179.39 (s, C_{quat}), 161.79 (s, C_{quat}), 145.35 (s, C_{quat}), 138.14 (s, CH), 137.87 (s, C_{quat}), 131.22 (s, CH), 131.02 (s, CH), 125.07 (s, C_{quat}), 114.32 (s, CH), 55.56 (s, CH_3).

IR (neat, cm^{-1}): ṽ: 3845, 3330, 3267, 3061, 3027, 2925, 2844, 2324, 2074, 1979, 1905, 1768, 1673, 1635, 1594, 1507, 1460, 1433, 1336, 1267, 1239, 1173, 1116, 1020, 987, 920, 870, 829, 799, 722.

ESI-HRMS: $[M]^+$ *m/z*: calculated for $[C_{13}H_9BrO_3]^+$ 291.97296, found 291.97402.

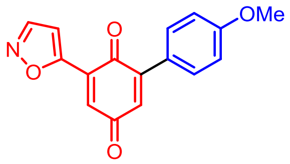

Chemical Formula: $C_{16}H_{11}NO_4$

18c: Following the general procedure A, the product was converted from 4-Fluoro-2-(5-isoxazolyl)phenol and anisole at 90 °C for 18 hours. The crude mixture is purified by SiO_2 gel column chromatography with petane/EA (from 40/1 to 10/1). Isolated yield: 63% (brown solid).

^{1}H NMR (600 MHz, Chloroform-*d*) δ 8.32 (d, *J* = 1.8 Hz, 1H), 7.42 (d, *J* = 9.0 Hz, 2H), 7.35 (d, *J* = 2.7 Hz, 1H), 7.20 (s, 1H), 6.93 (d, *J* = 8.3 Hz, 2H), 6.83 (d, *J* = 3.4 Hz, 1H), 3.80 (s, 3H).

^{13}C NMR (151 MHz, Chloroform-*d*) δ 186.75 (s, C_{quat}), 183.96 (s, C_{quat}), 161.75 (s, C_{quat}), 160.98 (s, C_{quat}), 151.45 (s, CH), 145.87 (s, C_{quat}), 131.79 (s, CH), 131.66 (s, C_{quat}), 131.51 (s, CH), 131.11 (s, CH), 124.92 (s, C_{quat}), 114.37 (s, CH), 108.52 (s, CH), 55.59 (s, CH_3).

IR (neat, cm^{-1}): ṽ: 3858, 3330, 3277, 3185, 3123, 3050, 2922, 2851, 2699, 2322, 2193, 2166, 2083, 2034, 1970, 1897, 1740, 1671, 1636, 1600, 1547, 1511, 1485, 1422, 1372, 1276, 1241, 1179, 1119, 1020, 959, 912, 829, 800, 726, 664.

ESI-HRMS: $[M+Na]^+$ *m/z*: calculated for $[C_{16}H_{11}NO_4Na]^+$ 304.05803, found 304.05841.

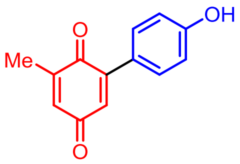

Chemical Formula: $C_{13}H_{10}O_3$

19c: Following the general procedure A, the product was converted from 4-fluoro-2-methylphenol and phenol at 90 °C for 18 hours. The crude mixture is purified by SiO_2 gel column chromatography with DCM/EA (40/1). Isolated yield: 63% (black solid).

^{1}H NMR (600 MHz, Chloroform-*d*) δ 7.41 (d, *J* = 8.4 Hz, 2H), 6.89 (d, *J* = 8.4 Hz, 2H), 6.75 (d, *J* = 2.9 Hz, 1H), 6.66 (d, *J* = 2.4 Hz, 1H), 5.14 (s, 1H), 2.12 (s, 3H).

^{13}C NMR (151 MHz, Chloroform-*d*) δ 188.03 (s, C_{quat}), 187.62 (s, C_{quat}), 157.62 (s, C_{quat}), 146.35 (s, C_{quat}), 145.71 (s, C_{quat}), 133.34 (s, CH), 131.40 (s, CH), 131.20 (s, CH), 125.69 (s, C_{quat}), 115.70 (s, CH), 16.51 (s, CH_3).

IR (neat, cm^{-1}): ṽ: 3847, 3587, 3281, 3058, 2924, 2854, 2676, 2624, 2496, 2320, 2163, 2068, 1984, 1908, 1724, 1646, 1589, 1511, 1449, 1378, 1310, 1247, 1179, 1116, 1077, 1009, 961, 912, 837, 726, 666.

ESI-HRMS: $[M+Na]^+$ *m/z*: calculated for $[C_{13}H_{10}O_3Na]^+$ 237.05222, found 237.05223.

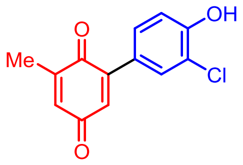

Chemical Formula: $C_{13}H_9ClO_3$

20c: Following the general procedure A, the product was converted from 4-fluoro-2-methylphenol and 2-chlorophenol at 90 °C for 18 hours. The crude mixture is purified by SiO_2 gel column chromatography with DCM. Isolated yield: 44% (black solid).

^{1}H NMR (600 MHz, Chloroform-*d*) δ 7.47 (d, *J* = 2.3 Hz, 1H), 7.30 – 7.23 (m, 1H), 7.01 (d, *J* = 8.3 Hz, 1H), 6.69 (d, *J* = 2.5 Hz, 1H), 6.60 (d, *J* = 2.2 Hz, 1H), 5.73 (s, 1H), 2.06 (s, 3H).

^{13}C NMR (151 MHz, Chloroform-*d*) δ 187.53 (s, C_{quat}), 187.14 (s, C_{quat}), 153.07 (s, C_{quat}), 146.27 (s, C_{quat}), 144.40 (s, C_{quat}), 133.44 (s, CH), 132.06 (s, CH), 130.23 (s, CH), 129.68 (s, CH), 126.53 (s, C_{quat}), 120.30 (s, C_{quat}), 116.49 (s, CH), 16.49 (s, CH_3).

IR (neat, cm^{-1}): ṽ: 3845, 3470, 3260, 3042, 2922, 2854, 2662, 2564, 2323, 2168, 2081, 2018, 1803, 1648, 1587, 1500, 1409, 1353, 1284, 1253, 1165, 1082, 1053, 1006, 951, 918, 891, 809, 780, 701, 674.

ESI-HRMS: $[M+H]^+$ *m/z*: calculated for $[C_{13}H_{10}ClO_3]^+$ 249.03130, found 249.03131.

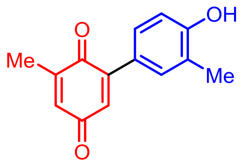

Chemical Formula: $C_{14}H_{12}O_3$

21c: Following the general procedure A, the product was converted from 4-fluoro-2-methylphenol and *o*-cresol at 90 °C for 18 hours. The crude mixture is purified by SiO_2 gel column chromatograph with DCM. Isolated yield: 60% (black solid).

^{1}H NMR (600 MHz, Chloroform-*d*) δ 7.28 (d, *J* = 2.1 Hz, 1H), 7.23 (dd, *J* = 8.2, 2.5 Hz, 1H), 6.82 (d, *J* = 8.3 Hz, 1H), 6.74 (d, *J* = 2.7 Hz, 1H), 6.65 (s, 1H), 5.53 (s, 1H), 2.28 (s, 3H), 2.12 (s, 3H).

^{13}C NMR (151 MHz, Chloroform-*d*) δ 187.96 (s, C_{quat}), 187.62 (s, C_{quat}), 155.92 (s, C_{quat}), 146.19 (s, C_{quat}), 145.80 (s, C_{quat}), 133.16 (s, CH), 132.15 (s, CH), 131.07 (s, CH), 128.51 (s, CH), 125.38 (s, C_{quat}), 124.23 (s, C_{quat}), 115.05 (s, CH), 16.34 (s, CH_3), 15.81 (s, CH_3).
IR (neat, cm^{-1}): ṽ: 3831, 3529, 3277, 3045, 2924, 2854, 2733, 2518, 2322, 2161, 2079, 1828, 1648, 1581, 1506, 1456, 1411, 1380, 1355, 1315, 1269, 1177, 1119, 1082, 994, 910, 806, 779, 675.
ESI-HRMS: $[M+Na]^+$ *m/z*: calculated for $[C_{14}H_{12}O_3Na]^+$ 251.06787, found 251.06807.

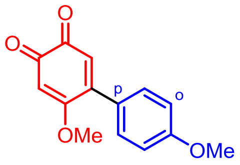

Chemical Formula: $C_{14}H_{12}O_4$

1e: Following the general procedure A, the product was converted from 2-fluoro-4-methoxyphenol and anisole at 90 °C for 18 hours. The crude mixture is purified by SiO_2 gel column chromatograph with DCM. Isolated yield: 53% (gold solid).
^1H NMR (600 MHz, Chloroform-*d*) δ 7.41 (d, *J* = 9.1 Hz, 2H), 6.89 (d, *J* = 9.1 Hz, 2H), 6.70 (s, 1H), 5.95 (s, 1H), 3.79 (s, 3H), 3.78 (s, 3H).
^{13}C NMR (151 MHz, Chloroform-*d*) δ 187.09 (s, C_{quat}), 182.18 (s, C_{quat}), 161.48 (s, C_{quat}), 158.65 (s, C_{quat}), 145.80 (s, C_{quat}), 131.21 (s, CH), 129.22 (s, CH), 125.03 (s, C_{quat}), 114.09 (s, CH), 107.94 (s, CH), 56.30 (s, CH_3), 55.39 (s, CH_3).
IR (neat, cm^{-1}): ṽ: 3871, 3263, 3036, 2923, 2851, 2516, 2303, 2048, 1890, 1720, 1653, 1581, 1502, 1454, 1358, 1304, 1261, 1176, 1022, 921, 873, 825, 735, 663.
ESI-HRMS: $[M+Na]^+$ *m/z*: calculated for $[C_{14}H_{12}O_4Na]^+$ 267.06278, found 267.06210.

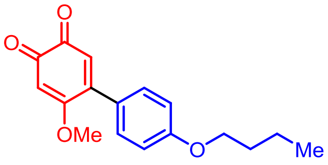

Chemical Formula: $C_{17}H_{18}O_4$

2e: Following the general procedure A, the product was converted from 2-fluoro-4-methoxyphenol and butoxybenzene at 90 °C for 18 hours. The crude mixture is purified by SiO_2 gel column chromatograph with DCM. Isolated yield: 50% (yellow solid).
^1H NMR (600 MHz, Chloroform-*d*) δ 7.40 (d, *J* = 8.9 Hz, 2H), 6.87 (d, *J* = 8.7 Hz, 2H), 6.69 (s, 1H), 5.94 (s, 1H), 3.94 (t, *J* = 6.5 Hz, 2H), 3.79 (s, 3H), 1.76 – 1.67 (m, 2H), 1.48 – 1.37 (m, 2H), 0.91 (t, *J* = 7.3 Hz, 3H).
^{13}C NMR (151 MHz, Chloroform-*d*) δ 187.30 (s, C_{quat}), 182.35 (s, C_{quat}), 161.28 (s, C_{quat}), 158.79 (s, C_{quat}), 146.00 (s, C_{quat}), 131.34 (s, CH), 129.22 (s, CH), 124.92 (s, C_{quat}), 114.75 (s, CH), 108.09 (s, CH), 67.99 (s, CH_2), 56.44 (s, CH_3), 31.31 (s, CH_2), 19.34 (s, CH_2), 13.94 (s, CH_3).
IR (neat, cm^{-1}): ṽ: 3271, 3057, 2923, 2866, 2312, 2190, 2101, 1898, 1837, 1744, 1640, 1588, 1503, 1460, 1367, 1295, 1257, 1177, 1067, 1003, 919, 870, 826, 734, 665.
ESI-HRMS: $[M+Na]^+$ *m/z*: calculated for $[C_{17}H_{18}O_4Na]^+$ 309.10973, found 309.10910.

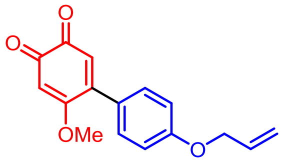

Chemical Formula: $C_{16}H_{14}O_4$

3e: Following the general procedure A, the product was converted from 2-fluoro-4-methoxyphenol and allyl phenyl ether at 90 °C for 18 hours. The crude mixture is purified by SiO_2 gel column chromatograph with DCM. Isolated yield: 44% (gold solid).

^{1}H NMR (600 MHz, Chloroform-*d*) δ 7.40 (d, *J* = 9.1 Hz, 2H), 6.90 (d, *J* = 9.1 Hz, 2H), 6.70 (s, 1H), 6.02 – 5.95 (m, 1H), 5.94 (s, 1H), 5.43 – 5.32 (m, 1H), 5.30 – 5.19 (m, 1H), 4.52 (d, *J* = 5.2 Hz, 2H), 3.79 (s, 3H).

^{13}C NMR (151 MHz, Chloroform-*d*) δ 187.24 (s, C_{quat}), 182.33 (s, C_{quat}), 160.65 (s, C_{quat}), 158.80 (s, C_{quat}), 145.91 (s, C_{quat}), 132.88 (s, CH), 131.34 (s, CH), 129.40 (s, CH), 125.31 (s, C_{quat}), 118.18 (s, CH_2), 114.99 (s, CH), 108.10 (s, CH), 69.01 (s, CH_3), 56.46 (s, CH_2).

IR (neat, cm^{-1}): ṽ: 3881, 3407, 3265, 3046, 2921, 2857, 2624, 2516, 2424, 2324, 2208, 2093, 2006, 1878, 1744, 1646, 1577, 1502, 1423, 1359, 1304, 1252, 1176, 998, 927, 870, 828, 732, 682.

ESI-HRMS: $[M+Na]^+$ *m/z*: calculated for $[C_{16}H_{14}O_4Na]^+$ 293.07843, found 293.07782.

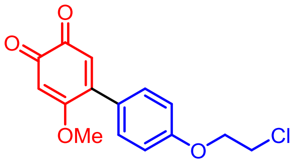

Chemical Formula: $C_{15}H_{13}ClO_4$

4e: Following the general procedure A, the product was converted from 2-fluoro-4-methoxyphenol and (2-chloroethoxy)benzene at 90 °C for 18 hours. The crude mixture is purified by SiO_2 gel column chromatograph with firstly DCM then pentane/EA (from 50/1 to 10/1). Isolated yield: 41% (orange solid).

^{1}H NMR (600 MHz, Chloroform-*d*) δ 7.42 (d, *J* = 8.8 Hz, 2H), 6.91 (d, *J* = 8.6 Hz, 2H), 6.71 (s, 1H), 5.95 (s, 1H), 4.21 (t, *J* = 5.9 Hz, 2H), 3.80 (s, 3H), 3.77 (t, *J* = 5.7 Hz, 2H).

^{13}C NMR (151 MHz, Chloroform-*d*) δ 187.15 (s, C_{quat}), 182.32 (s, C_{quat}), 160.16 (s, C_{quat}), 158.81 (s, C_{quat}), 145.81 (s, C_{quat}), 131.45 (s, CH), 129.64 (s, CH), 125.88 (s, C_{quat}), 114.87 (s, CH), 108.10 (s, CH), 68.17 (s, CH_2), 56.49 (s, CH_3), 41.81 (s, CH_2).

IR (neat, cm^{-1}): ṽ: 3851, 3270, 3059, 2923, 2857, 2511, 2293, 2180, 2082, 1903, 1837, 1719, 1641, 1589, 1504, 1455, 1368, 1299, 1262, 1180, 1080, 1036, 1000, 920, 873, 826, 732, 666.

ESI-HRMS: $[M+Na]^+$ *m/z*: calculated for $[C_{15}H_{13}ClO_4Na]^+$ 315.03946, found 315.03897.

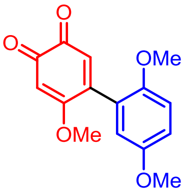

Chemical Formula: $C_{15}H_{14}O_5$

5e: Following the general procedure A, the product was converted from 2-fluoro-4-methoxyphenol

and 1,4-dimethoxybenzene at 90 °C for 18 hours. The crude mixture is purified by SiO_2 gel column chromatograph with DCM. Isolated yield: 31% (brown solid).

^{1}H NMR (600 MHz, Chloroform-*d*) δ 6.87 (dd, *J* = 9.1, 2.8 Hz, 1H), 6.83 (d, *J* = 9.2 Hz, 1H), 6.70 (s, 1H), 6.66 (d, *J* = 3.5 Hz, 1H), 5.96 (s, 1H), 3.79 (s, 3H), 3.71 (s, 3H), 3.67 (s, 3H).

^{13}C NMR (151 MHz, Chloroform-*d*) δ 185.71 (s, C_{quat}), 182.27 (s, C_{quat}), 158.40 (s, C_{quat}), 153.39 (s, C_{quat}), 151.46 (s, C_{quat}), 146.17 (s, C_{quat}), 132.60 (s, CH), 123.22 (s, C_{quat}), 116.31 (s, CH), 116.00 (s, CH), 112.54 (s, CH), 108.00 (s, CH), 56.35 (s, CH_3), 56.23 (s, CH_3), 55.81 (s, CH_3).

IR (neat, cm^{-1}): ṽ: 3828, 3293, 2924, 2850, 2293, 2197, 2099, 2001, 1850, 1737, 1648, 1602, 1490, 1452, 1371, 1281, 1194, 1008, 910, 851, 810, 750, 717.

ESI-HRMS: $[M+Na]^+$ *m/z*: calculated for $[C_{15}H_{14}O_5Na]^+$ 297.07334, found 297.07303.

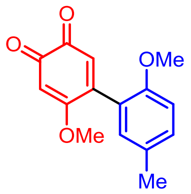

Chemical Formula: $C_{15}H_{14}O_4$

6e: Following the general procedure A, the product was converted from 2-fluoro-4-methoxyphenol and 1-methoxy-4-methylbenzene at 90 °C for 18 hours. The crude mixture is purified by SiO_2 gel column chromatograph with DCM. Isolated yield: 46% (dark red solid).

^{1}H NMR (600 MHz, Chloroform-*d*) δ 7.13 (d, *J* = 8.3 Hz, 1H), 6.89 (s, 1H), 6.79 (d, *J* = 8.4 Hz, 1H), 6.68 (s, 1H), 5.96 (s, 1H), 3.79 (s, 3H), 3.68 (s, 3H), 2.24 (s, 3H).

^{13}C NMR (151 MHz, Chloroform-*d*) δ 186.09 (s, C_{quat}), 182.52 (s, C_{quat}), 158.55 (s, C_{quat}), 155.36 (s, C_{quat}), 146.70 (s, C_{quat}), 132.54 (s, CH), 131.73 (s, CH), 131.26 (s, CH), 130.02 (s, C_{quat}), 122.44 (s, C_{quat}), 111.45 (s, CH), 108.15 (s, CH), 56.36 (s, CH_3), 56.00 (s, CH_3), 20.52 (s, CH_3).

IR (neat, cm^{-1}): ṽ: 3826, 3283, 3060, 2921, 2854, 2648, 2289, 2226, 2105, 1972, 1866, 1735, 1659, 1593, 1497, 1453, 1353, 1195, 1014, 868, 791, 739, 692.

ESI-HRMS: $[M+Na]^+$ *m/z*: calculated for $[C_{15}H_{14}O_4Na]^+$ 281.07843, found 281.07782

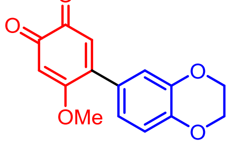

Chemical Formula: $C_{15}H_{12}O_5$

7e: Following the general procedure A, the product was converted from 2-fluoro-4-methoxyphenol and benzo-1,4-dioxane at 90 °C for 18 hours. The crude mixture is purified by SiO_2 gel column chromatograph with DCM. Isolated yield: 52% (brown solid).

^{1}H NMR (600 MHz, Chloroform-*d*) δ 7.00 (d, *J* = 2.6 Hz, 1H), 6.95 (dd, *J* = 9.1, 1.8 Hz, 1H), 6.84 (d, *J* = 8.3 Hz, 1H), 6.67 (s, 1H), 5.94 (s, 1H), 4.25 – 4.19 (m, 4H), 3.79 (s, 3H).

^{13}C NMR (151 MHz, Chloroform-*d*) δ 187.01 (s, C_{quat}), 182.28 (s, C_{quat}), 158.74 (s, C_{quat}), 145.93 (s, C_{quat}), 145.77 (s, C_{quat}), 143.60 (s, C_{quat}), 129.75 (s, CH), 126.04 (s, C_{quat}), 123.30 (s, CH), 118.93 (s, CH), 117.63 (s, CH), 108.12 (s, CH), 64.74 (s, CH_2), 64.36 (s, CH_2), 56.45 (s, CH_3).

IR (neat, cm^{-1}): ṽ: 3885, 3462, 3262, 3063, 2931, 2646, 2320, 2093, 1899, 1731, 1654, 1574, 1501, 1448, 1357, 1283, 1189, 1132, 1059, 1003, 972, 871, 821, 735.
ESI-HRMS: $[M+Na]^+$ *m/z*: calculated for $[C_{15}H_{12}O_5Na]^+$ 295.05769, found 295.05707.

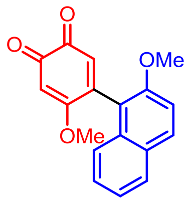

Chemical Formula: $C_{18}H_{14}O_4$

8e: Following the general procedure A, the product was converted from 2-fluoro-4-methoxyphenol and 2-methoxynaphthalene at 90 °C for 18 hours. The crude mixture is purified by SiO_2 gel column chromatograph with DCM. Isolated yield: 40% (maroon solid).
^{1}H NMR (600 MHz, Chloroform-*d*) δ 7.86 (d, *J* = 9.0 Hz, 1H), 7.74 (d, *J* = 8.6 Hz, 1H), 7.46 (d, *J* = 8.5 Hz, 1H), 7.34 (t, *J* = 7.5 Hz, 1H), 7.30 – 7.24 (m, 2H), 6.71 (s, 1H), 6.05 (s, 1H), 3.83 (s, 3H), 3.80 (s, 3H).
^{13}C NMR (151 MHz, Chloroform-*d*) δ 186.09 (s, C_{quat}), 182.22 (s, C_{quat}), 158.78 (s, C_{quat}), 154.60 (s, C_{quat}), 145.80 (s, C_{quat}), 135.05 (s, CH), 132.56 (s, C_{quat}), 131.37 (s, CH), 129.02 (s, C_{quat}), 128.49 (s, CH), 127.35 (s, CH), 124.00 (s, CH), 123.81 (s, CH), 116.35 (s, C_{quat}), 113.16 (s, CH), 108.44 (s, CH), 56.74 (s, CH_3), 56.42 (s, CH_3).
IR (neat, cm^{-1}): ṽ: 3907, 3289, 3066, 3013, 2922, 2851, 2527, 2321, 2204, 2093, 1829, 1751, 1657, 1595, 1507, 1462, 1371, 1328, 1260, 1181, 1101, 1061, 997, 950, 901, 855, 809, 744, 661.
ESI-HRMS: $[M+Na]^+$ *m/z*: calculated for $[C_{18}H_{14}O_4Na]^+$ 317.07843, found 317.07791.

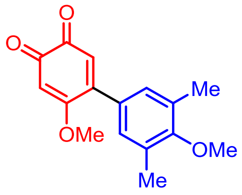

Chemical Formula: $C_{16}H_{16}O_4$

9e: Following the general procedure A, the product was converted from 2-fluoro-4-methoxyphenol and 2-methoxy-1,3-dimethylbenzene at 90 °C for 18 hours. The crude mixture is purified by SiO_2 gel column chromatograph with DCM. Isolated yield: 35% (orange solid).
^{1}H NMR (600 MHz, Chloroform-*d*) δ 7.08 (s, 2H), 6.68 (s, 1H), 5.95 (s, 1H), 3.80 (s, 3H), 3.68 (s, 3H), 2.25 (s, 6H).
^{13}C NMR (151 MHz, Chloroform-*d*) δ 187.15 (s, C_{quat}), 182.34 (s, C_{quat}), 159.19 (s, C_{quat}), 158.77 (s, C_{quat}), 146.42 (s, C_{quat}), 131.37 (s, C_{quat}), 130.35 (s, CH), 130.26 (s, CH), 128.32 (s, C_{quat}), 108.12 (s, CH), 59.86 (s, CH_3), 56.47 (s, CH_3), 16.37 (s, CH_3).
IR (neat, cm^{-1}): ṽ: 3866, 3318, 3272, 3062, 2922, 2855, 2586, 2427, 2289, 2186, 2093, 2030, 1972, 1930, 1851, 1714, 1630, 1591, 1478, 1362, 1294, 1200, 1149, 1057, 1006, 926, 876, 763, 703.
ESI-HRMS: $[M+Na]^+$ *m/z*: calculated for $[C_{16}H_{16}O_4Na]^+$ 295.09408, found 295.09351.

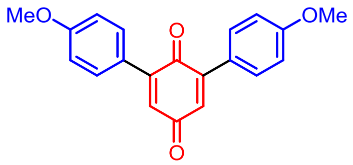

Chemical Formula: $C_{20}H_{16}O_4$

1g: Following the general procedure B, the product was converted from 4-fluorophenol and anisole at -20 °C for 18 hours. The crude mixture is purified by SiO_2 gel column chromatograph with DCM. Isolated yield: 62% (dark red solid).

^{1}H NMR (600 MHz, Chloroform-*d*) δ 7.42 (d, *J* = 8.3 Hz, 4H), 6.90 (d, *J* = 8.3 Hz, 4H), 6.78 (s, 2H), 3.78 (s, 6H).

^{13}C NMR (151 MHz, Chloroform-*d*) δ 187.85 (s, C_{quat}), 187.08 (s, C_{quat}), 161.35 (s, C_{quat}), 145.93 (s, C_{quat}), 131.23 (s, CH), 131.09 (s, CH), 125.71 (s, C_{quat}), 114.16 (s, CH), 55.52 (s, CH_3).

IR (neat, cm^{-1}): ṽ: 3865, 3470, 3209, 2929, 2843, 2555, 2304, 2207, 2158, 2048, 1979, 1899, 1752, 1642, 1597, 1506, 1450, 1358, 1306, 1244, 1177, 1108, 1022, 914, 876, 826, 785, 736, 663.

ESI-HRMS: $[M+Na]^+$ *m/z*: calculated for $[C_{20}H_{16}O_4Na]^+$ 343.09408, found 343.09241.

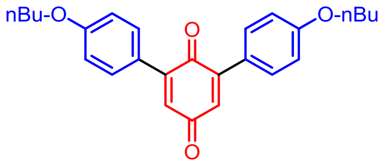

Chemical Formula: $C_{26}H_{28}O_4$

2g: Following the general procedure B, the product was converted from 4-fluorophenol and butoxybenzene at -20 °C for 18 hours. The crude mixture is purified by SiO_2 gel column chromatograph with DCM. Isolated yield: 60% (orange solid).

^{1}H NMR (600 MHz, Chloroform-*d*) δ 7.40 (d, *J* = 8.7 Hz, 4H), 6.87 (d, *J* = 9.0 Hz, 4H), 6.76 (s, 2H), 3.93 (t, *J* = 6.5 Hz, 4H), 1.77 – 1.65 (m, 4H), 1.48 – 1.35 (m, 4H), 0.90 (t, *J* = 7.4 Hz, 6H).

^{13}C NMR (151 MHz, Chloroform-*d*) δ 187.85 (s, C_{quat}), 187.14 (s, C_{quat}), 160.96 (s, C_{quat}), 145.94 (s, C_{quat}), 131.05 (s, CH), 125.45 (s, C_{quat}), 114.63 (s, CH), 67.93 (s, CH_2), 31.31 (s, CH_2), 19.33 (s, CH_2), 13.95 (s, CH_3).

IR (neat, cm^{-1}): ṽ: 3291, 3200, 3042, 2932, 2871, 2476, 2302, 2154, 2055, 2003, 1907, 1773, 1647, 1599, 1503, 1471, 1387, 1321, 1282, 1242, 1177, 1114, 1070, 997, 970, 912, 876, 835, 740, 669.

ESI-HRMS: $[M+Na]^+$ *m/z*: calculated for $[C_{26}H_{28}O_4Na]^+$ 427.18798, found 427.18570.

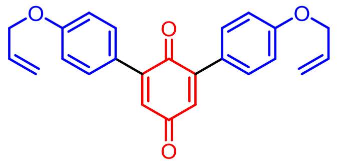

Chemical Formula: $C_{24}H_{20}O_4$

3g: Following the general procedure B, the product was converted from 4-fluorophenol and allyl phenyl ether at -20 °C for 18 hours. The crude mixture is purified by SiO_2 gel column chromatograph with DCM. Isolated yield: 51% (dark orange solid).

^{1}H NMR (400 MHz, Chloroform-*d*) δ 7.55 – 7.40 (m, 4H), 7.03 – 6.93 (m, 4H), 6.85 (s, 2H), 6.15 – 5.96 (m, 2H), 5.49 – 5.20 (m, 4H), 4.65 – 4.53 (m, 4H).

^{13}C NMR (101 MHz, Chloroform-*d*) δ 187.82 (s, C_{quat}), 187.07 (s, C_{quat}), 160.38 (s, C_{quat}), 145.90 (s, C_{quat}), 132.92 (s, CH), 131.26 (s, CH), 131.08 (s, CH), 125.84 (s, C_{quat}), 118.16 (s, CH_2), 114.91 (s, CH), 68.99 (s, CH_2).

IR (neat, cm^{-1}): ṽ: 3366, 3200, 3032, 2922, 2863, 2553, 2310, 2206, 2156, 2060, 1996, 1874, 1754, 1657, 1597, 1506, 1454, 1417, 1368, 1241, 1177, 1110, 1063, 999, 923, 831,

ESI-HRMS: $[M+Na]^+$ *m/z*: calculated for $[C_{24}H_{20}O_4Na]^+$ 395.12538, found 395.12323.

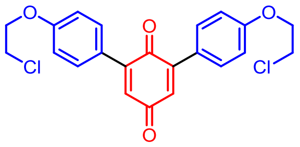

Chemical Formula: $C_{22}H_{18}Cl_2O_4$

4g: Following the general procedure B, the product was converted from 4-fluorophenol and (2-chloroethoxy)benzene at -20 °C for 18 hours. The crude mixture is purified by SiO_2 gel column chromatograph with DCM. Isolated yield: 56% (dark red solid).

^{1}H NMR (400 MHz, Chloroform-*d*) δ 7.64 – 7.42 (m, 4H), 7.04 – 6.91 (m, 4H), 6.86 (s, 2H), 4.28 (t, *J* = 5.8 Hz, 4H), 3.84 (t, *J* = 5.8 Hz, 4H).

^{13}C NMR (101 MHz, Chloroform-*d*) δ 187.57 (s, C_{quat}), 186.75 (s, C_{quat}), 159.80 (s, C_{quat}), 145.65 (s, C_{quat}), 131.37 (s, CH), 131.05 (s, CH), 126.25 (s, C_{quat}), 114.69 (s, CH), 68.03 (s, CH_2), 41.68 (s, CH_2).

IR (neat, cm^{-1}): ṽ: 3292, 3201, 3045, 2923, 2861, 2488, 2306, 2161, 2056, 1910, 1744, 1648, 1599, 1505, 1454, 1426, 1385, 1240, 1180, 1112, 1035, 980, 895, 837, 730, 672.

APCI-HRMS: $[M+H]^+$ *m/z*: calculated for $[C_{22}H_{19}Cl_2O_4]^+$ 417.0655, found 417.0641.

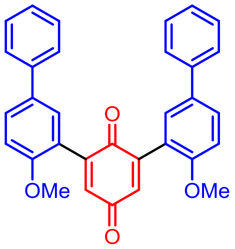

Chemical Formula: $C_{32}H_{24}O_4$

5g: Following the general procedure B, the product was converted from 4-fluorophenol and 4-methoxy-1,1'-biphenyl at -20 °C for 18 hours. The crude mixture is purified by SiO_2 gel column chromatograph with DCM. Isolated yield: 40% (orange solid).

^{1}H NMR (400 MHz, Chloroform-*d*) δ 7.64 (dd, *J* = 8.6, 2.4 Hz, 2H), 7.60 – 7.54 (m, 4H), 7.48 (d, *J* = 2.4 Hz, 2H), 7.43 (t, *J* = 7.6 Hz, 4H), 7.33 (t, *J* = 7.4 Hz, 2H), 7.06 (d, *J* = 8.6 Hz, 2H), 6.96 (s, 2H), 3.86 (s, 6H).

^{13}C NMR (101 MHz, Chloroform-*d*) δ 188.03 (s, C_{quat}), 184.53 (s, C_{quat}), 156.91 (s, C_{quat}), 146.49 (s, C_{quat}), 140.28 (s, C_{quat}), 134.19 (s, CH), 133.96 (s, C_{quat}), 129.74 (s, CH), 129.68 (s, CH), 128.92 (s, CH), 127.12 (s, CH), 126.93 (s, CH), 123.68 (s, C_{quat}), 111.78 (s, CH), 56.08 (s, CH_3).

IR (neat, cm^{-1}): ṽ: 3459, 3019, 2928, 2854, 2556, 2318, 2206, 2165, 2085, 1990, 1883, 1740, 1644, 1598, 1481, 1366, 1280, 1217, 1093, 1052, 1016, 906, 819, 758, 695.

APCI-HRMS: $[M+H]^+$ *m/z*: calculated for $[C_{32}H_{25}O_4]^+$ 473.1747, found 473.1733.

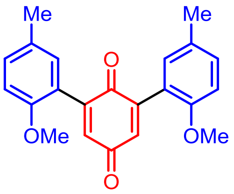

Chemical Formula: $C_{22}H_{20}O_4$

6g: Following the general procedure B, the product was converted from 4-fluorophenol and 1-methoxy-4-methylbenzene at -20 °C for 18 hours. The crude mixture is purified by SiO_2 gel column chromatograph with DCM. Isolated yield: 41% (dark orange solid).

^{1}H NMR (400 MHz, Chloroform-*d*) δ 7.18 (dd, *J* = 8.6, 2.3 Hz, 2H), 7.01 (d, *J* = 2.3 Hz, 2H), 6.86 (d, *J* = 8.5 Hz, 2H), 6.84 (s, 2H), 3.77 (s, 6H), 2.30 (s, 6H).

^{13}C NMR (101 MHz, Chloroform-*d*) δ 188.05 (s, C_{quat}), 184.61 (s, C_{quat}), 155.20 (s, C_{quat}), 146.57 (s, C_{quat}), 133.73 (s, CH), 131.38 (s, CH), 131.24 (s, CH), 129.82 (s, C_{quat}), 123.05 (s, C_{quat}), 111.29 (s, CH), 55.89 (s, CH_3), 20.39 (s, CH_3).

IR (neat, cm^{-1}): ṽ: 3767, 3399, 2926, 2848, 2638, 2307, 2159, 2081, 1984, 1941, 1741, 1649, 1590, 1494, 1244, 1185, 1141, 1093, 1024, 910, 797, 726, 662.

ESI-HRMS: $[M+Na]^+$ *m/z*: calculated for $[C_{22}H_{20}O_4Na]^+$ 371.12538, found 371.12436.

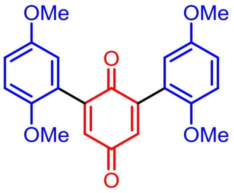

Chemical Formula: $C_{22}H_{20}O_6$

7g: Following the general procedure B, the product was converted from 4-fluorophenol and 1,4-dimethoxybenzene at -20 °C for 18 hours. The crude mixture is purified by SiO_2 gel column chromatograph with DCM. Isolated yield: 50% (brown solid).

^{1}H NMR (600 MHz, Chloroform-*d*) δ 6.87 (dd, *J* = 8.9, 3.0 Hz, 2H), 6.83 (d, *J* = 9.0 Hz, 2H), 6.79 (s, 2H), 6.71 (d, *J* = 3.5 Hz, 2H), 3.71 (s, 6H), 3.69 (s, 6H).

^{13}C NMR (151 MHz, Chloroform-*d*) δ 188.00 (s, C_{quat}), 184.29 (s, C_{quat}), 153.57 (s, C_{quat}), 151.60 (s, C_{quat}), 146.43 (s, C_{quat}), 134.13 (s, CH), 124.11 (s, C_{quat}), 116.46 (s, CH), 116.07 (s, CH), 112.70 (s, CH), 56.56 (s, CH_3), 55.96 (s, CH_3).

IR (neat, cm^{-1}): ṽ: 3884, 3391, 2923, 2853, 2320, 2211, 2160, 2057, 1979, 1913, 1740, 1650, 1583, 1494, 1458, 1275, 1217, 1036, 913, 866, 802, 719.

ESI-HRMS: $[M+Na]^+$ *m/z*: calculated for $[C_{22}H_{20}O_6Na]^+$ 403.11521, found 403.11295.

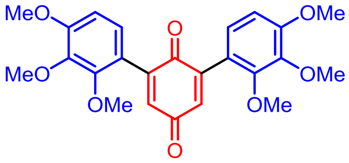

Chemical Formula: $C_{24}H_{24}O_8$

8g: Following the general procedure B, the product was converted from 4-fluorophenol and 1,2,3-trimethoxybenzene at -20 °C for 18 hours. The crude mixture is purified by SiO_2 gel column

chromatograph with DCM. Isolated yield: 44% (dark orange solid).

^{1}H NMR (600 MHz, Chloroform-*d*) δ 6.86 (d, *J* = 8.9 Hz, 2H), 6.73 (s, 2H), 6.64 (d, *J* = 8.8 Hz, 2H), 3.83 (s, 6H), 3.81 (s, 6H), 3.79 (s, 6H).

^{13}C NMR (151 MHz, Chloroform-*d*) δ 187.88 (s, C_{quat}), 185.35 (s, C_{quat}), 155.47 (s, C_{quat}), 152.14 (s, C_{quat}), 146.62 (s, C_{quat}), 142.20 (s, C_{quat}), 133.46 (s, CH), 125.10 (s, CH), 121.18 (s, C_{quat}), 107.18 (s, CH), 61.26 (s, CH_3), 60.92 (s, CH_3), 56.21 (s, CH_3).

IR (neat, cm^{-1}): ṽ: 3313, 2934, 2847, 2567, 2344, 2162, 2080, 1989, 1903, 1757, 1655, 1595, 1463, 1417, 1341, 1278, 1196, 1092, 991, 906, 834, 799, 692.

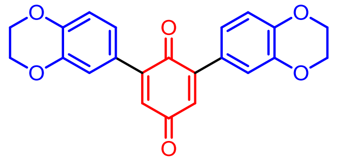

Chemical Formula: $C_{22}H_{16}O_6$

9g: Following the general procedure B, the product was converted from 4-fluorophenol and benzo-1,4-dioxane at -20 °C for 18 hours. The crude mixture is purified by SiO_2 gel column chromatograph with DCM. Isolated yield: 55% (dark orange solid).

^{1}H NMR (600 MHz, Chloroform-*d*) δ 7.01 (d, *J* = 2.6 Hz, 2H), 6.96 (dd, *J* = 8.6, 2.3 Hz, 2H), 6.86 (d, *J* = 8.3 Hz, 2H), 6.75 (s, 2H), 4.26 – 4.20 (m, 8H).

^{13}C NMR (151 MHz, Chloroform-*d*) δ 187.75 (s, C_{quat}), 186.69 (s, C_{quat}), 145.82 (s, C_{quat}), 145.67 (s, C_{quat}), 143.59 (s, C_{quat}), 131.49 (s, CH), 126.56 (s, C_{quat}), 123.08 (s, CH), 118.72 (s, CH), 117.57 (s, CH), 64.73 (s, CH_2), 64.38 (s, CH_2).

IR (neat, cm^{-1}): ṽ: 3271, 3049, 2924, 2589, 2254, 2070, 1991, 1739, 1637, 1577, 1501, 1418, 1275, 1104, 1060, 897, 822, 730, 680.

ESI-HRMS: $[M+Na]^+$ *m/z*: calculated for $[C_{22}H_{16}O_6Na]^+$ 399.08391, found 399.08209.

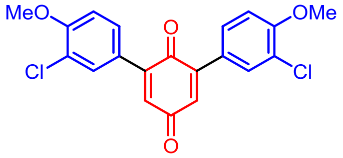

Chemical Formula: $C_{20}H_{14}Cl_2O_4$

10g: Following the general procedure B, the product was converted from 4-fluorophenol and 1-chloro-2-methoxybenzene at -20 °C for 18 hours. The crude mixture is purified by SiO_2 gel column chromatograph with DCM. Isolated yield: 58% (red solid).

^{1}H NMR (600 MHz, Chloroform-*d*) δ 7.53 (d, *J* = 2.6 Hz, 2H), 7.37 (dd, *J* = 8.3, 2.6 Hz, 2H), 6.93 (d, *J* = 9.0 Hz, 2H), 6.80 (s, 2H), 3.89 (s, 6H).

^{13}C NMR (151 MHz, Chloroform-*d*) δ 187.26 (s, C_{quat}), 186.22 (s, C_{quat}), 156.71 (s, C_{quat}), 144.75 (s, C_{quat}), 131.93 (s, CH), 131.37 (s, CH), 129.34 (s, CH), 126.25 (s, C_{quat}), 122.95 (s, C_{quat}), 111.93 (s, CH), 56.45 (s, CH_3).

IR (neat, cm^{-1}): ṽ: 3854, 3745, 3451, 3296, 3185, 2927, 2854, 2649, 2558, 2460, 2309, 2171, 2065, 1978, 1912, 1746, 1650, 1589, 1498, 1453, 1394, 1344, 1263, 1188, 1065, 1013, 888, 830, 782, 707.

ESI-HRMS: $[M+Na]^+$ *m/z*: calculated for $[C_{20}H_{14}Cl_2O_4Na]^+$ 411.01614, found 411.01453.

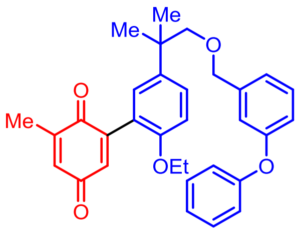

Chemical Formula: $C_{32}H_{32}O_5$

22c: Following the general procedure A, the product was converted from 4-fluoro-2-methylphenol and Etofenprox at 90 °C for 18 hours. The crude mixture is purified by SiO_2 gel column chromatograph with DCM. Isolated yield: 42% (dark orange oil).

^{1}H NMR (400 MHz, Chloroform-*d*) δ 7.36 – 7.21 (m, 3H), 7.21 – 7.14 (m, 1H), 7.07 (d, *J* = 2.4 Hz, 1H), 7.05 – 6.98 (m, 1H), 6.98 – 6.86 (m, 3H), 6.85 (s, 1H), 6.81 (dd, *J* = 7.9, 2.5 Hz, 1H), 6.76 (d, *J* = 8.7 Hz, 1H), 6.65 (d, *J* = 2.7 Hz, 1H), 6.55 (d, *J* = 2.6 Hz, 1H), 4.37 (s, 2H), 3.91 (q, *J* = 7.0 Hz, 2H), 3.33 (s, 2H), 2.03 (s, 3H), 1.24 (s, 6H), 1.19 (t, *J* = 7.0 Hz, 3H).

^{13}C NMR (101 MHz, Chloroform-*d*) δ 188.03 (s, C_{quat}), 186.30 (s, C_{quat}), 157.48 (s, C_{quat}), 157.28 (s, C_{quat}) 154.81 (s, C_{quat}), 146.99 (s, C_{quat}), 146.48 (s, C_{quat}), 141.01 (s, C_{quat}), 139.67 (s, C_{quat}), 134.15 (s, CH), 133.02 (s, CH), 129.84 (s, CH), 129.70 (s, CH), 128.84 (s, CH), 128.30 (s, CH), 123.37 (s, CH), 122.87 (s, C_{quat}), 122.11 (s, CH), 119.11 (s, CH), 117.79 (s, CH), 117.71 (s, CH), 111.94 (s, CH), 80.23 (s, CH_2), 72.88 (s, CH_2), 64.26 (s, CH_2), 38.70 (s, C_{quat}), 26.21 (s, CH_3), 16.51 (s, CH_3), 14.81 (s, CH_3).

IR (neat, cm^{-1}): ṽ: 3313, 3059, 2967, 2926, 2866, 2336, 2159, 2087, 1994, 1941, 1865, 1654, 1589, 1487, 1362, 1289, 1250, 1151, 1086, 1036, 915, 777, 692.

ESI-HRMS: $[M+Na]^+$ *m/z*: calculated for $[C_{32}H_{32}O_5Na]^+$ 519.21420, found 519.21277.

5. Other substrates

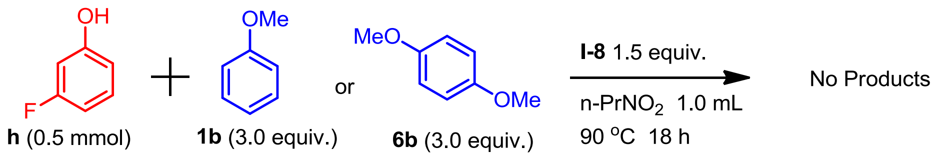

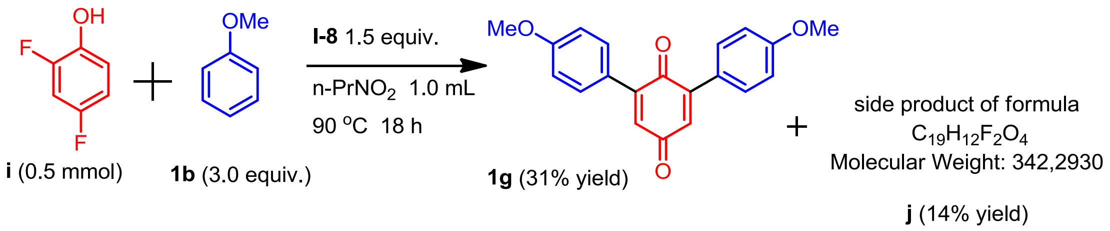

j: Following the general procedure A, the product was converted from 2,4-difluorophenol and anisole at 90 °C for 18 hours. The crude mixture is purified by SiO_2 gel column chromatograph with DCM. Isolated yield of **j**: 14% (red solid).

^{1}H NMR (600 MHz, Chloroform-*d*) δ 7.45 (d, *J* = 8.4 Hz, 2H), 7.13 (td, *J* = 8.8, 5.3 Hz, 1H), 6.98 – 6.85 (m, 4H), 6.70 (d, *J* = 2.5 Hz, 1H), 5.66 (d, *J* = 2.5 Hz, 1H), 3.80 (s, 3H).

^{13}C NMR (151 MHz, Chloroform-*d*) δ 187.15 (s, C_{quat}), 180.69 (s, C_{quat}), 161.60 (s, C_{quat}), 160.67 (dd, J = 249.5, 10.8 Hz, C_{quat}), 157.43 (s, C_{quat}), 153.91 (dd, J = 254.8, 12.7 Hz, C_{quat}), 144.01 (s, C_{quat}), 136.25 (dd, J = 12.3, 4.6 Hz, C_{quat}), 131.62 (s, CH), 130.97 (s, CH), 124.59 (s, CH), 124.05 (d, J = 9.8 Hz, C_{quat}), 114.34 (s, CH), 112.45 (dd, J = 23.0, 3.7 Hz, CH), 110.63 (s, CH), 106.21 (dd, J = 26.7, 21.7 Hz, CH), 55.56 (s, CH_3).

IR (neat, cm^{-1}): ṽ: 3259, 3083, 3000, 2930, 2837, 2523, 2291, 2196, 2159, 2043, 1997, 1898, 1788, 1676, 1638, 1598, 1503, 1458, 1434, 1359, 1305, 1283, 1248, 1182, 1142, 1078, 1028, 959, 894, 833, 763, 730, 670.

APCI-HRMS: $[M+H]^+$ *m/z*: calculated for $[C_{19}H_{13}F_2O_4]^+$ 343.0776, found 343.07665.

6. Mechanistic experiments

(a) Experiments to explore the effect of air on the reaction

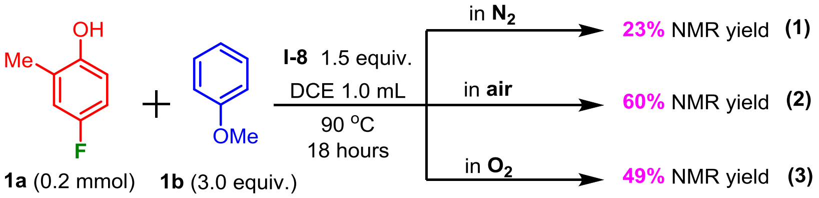

(b) in situ ^{19}F NMR experiments

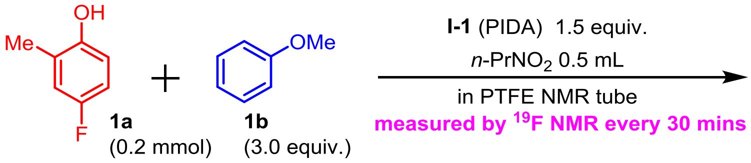

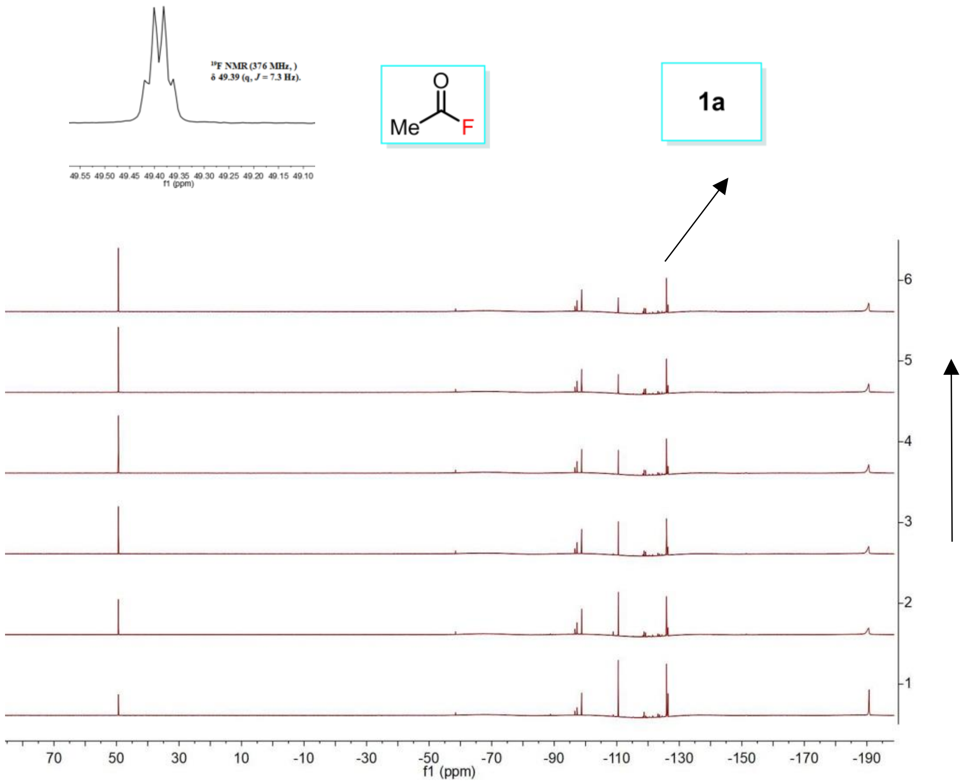

7. Copies of ^{1}H, ^{13}C and NOESY Spectra

^{1}H NMR (**I-8**)

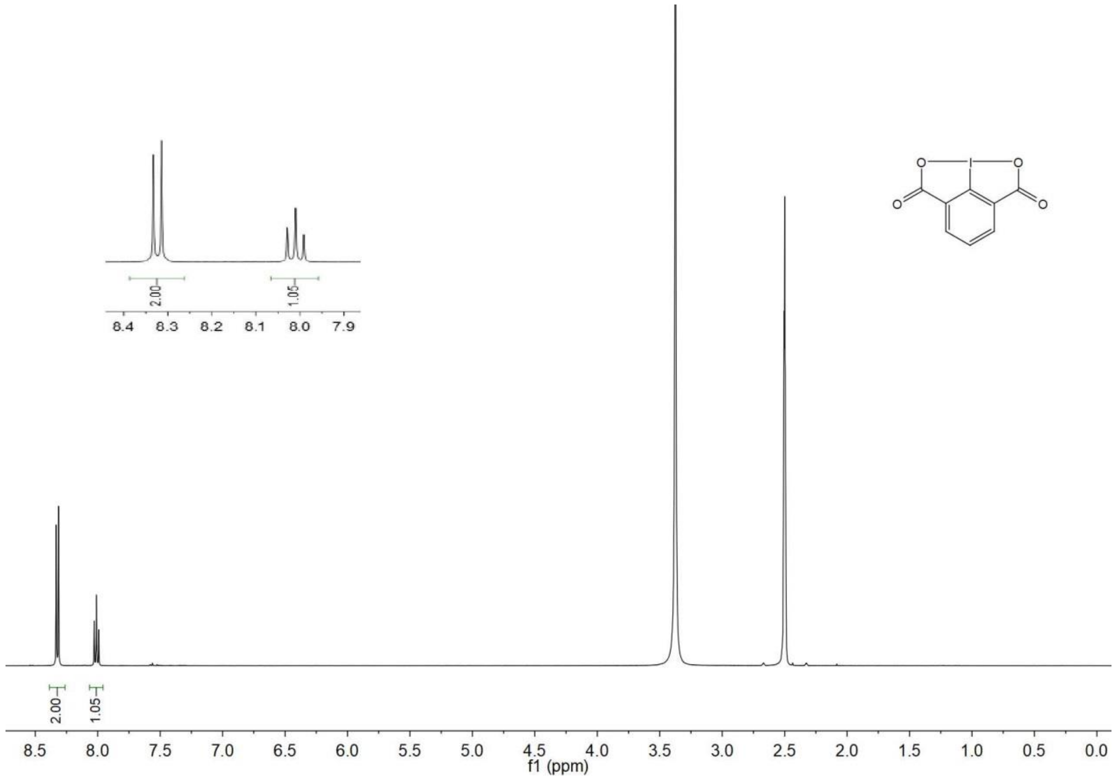

^{13}C NMR (**I-8**)

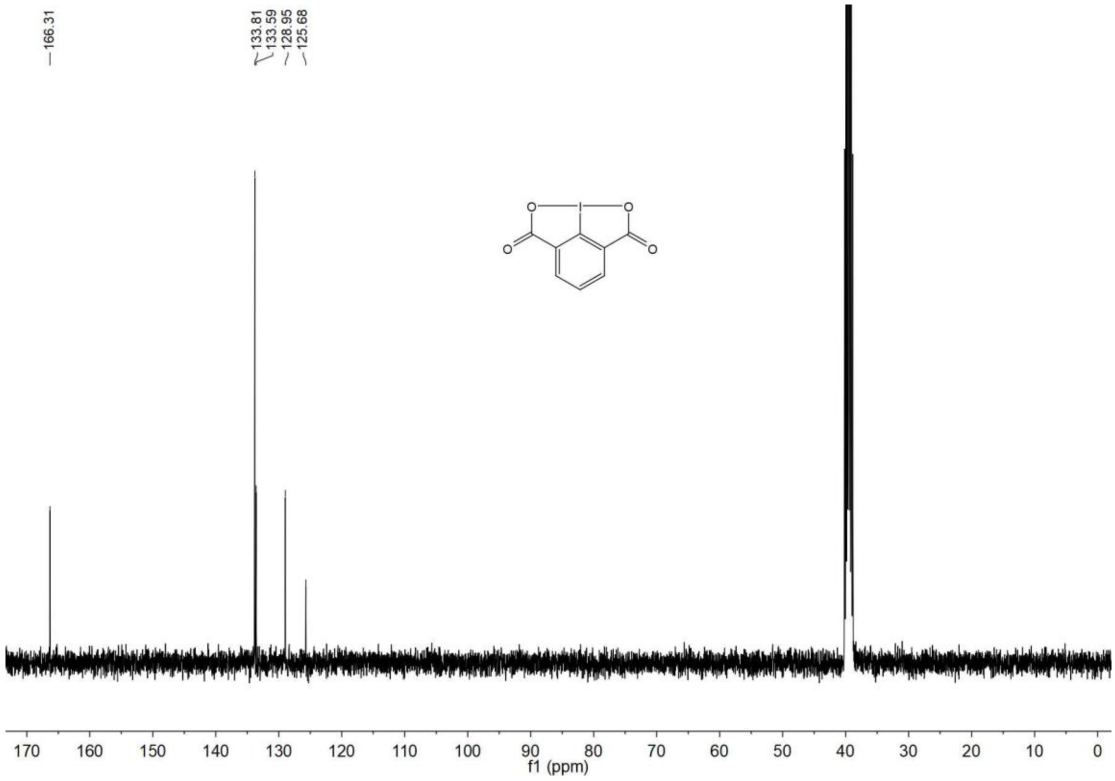

^{1}H NMR (**1c**)

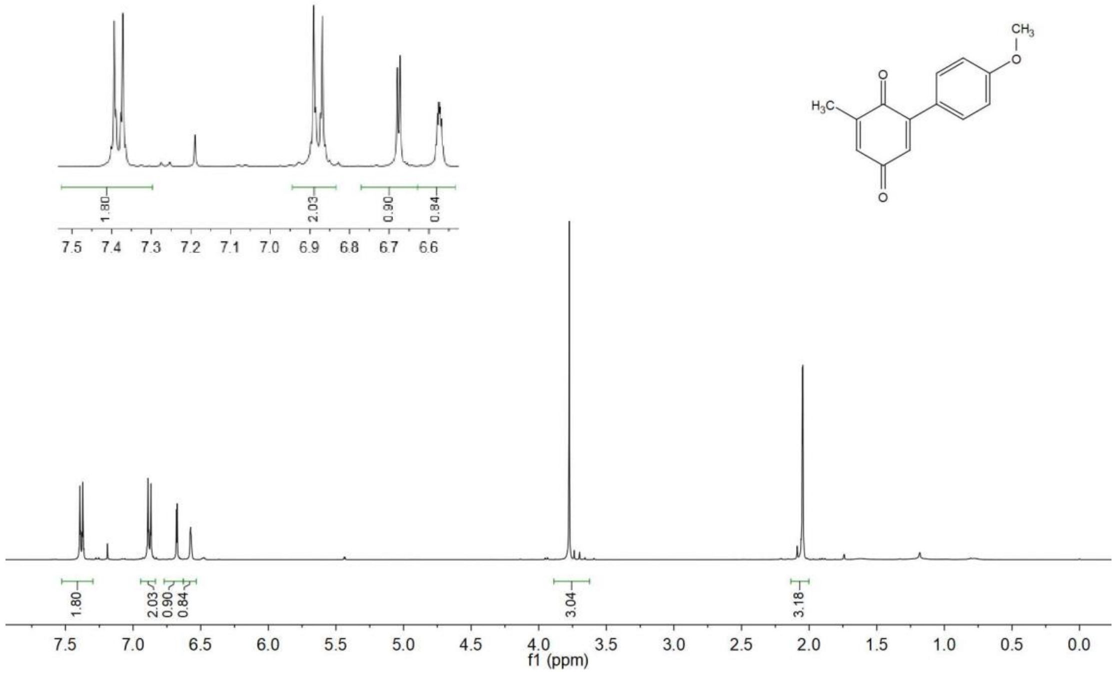

^{13}C NMR (**1c**)

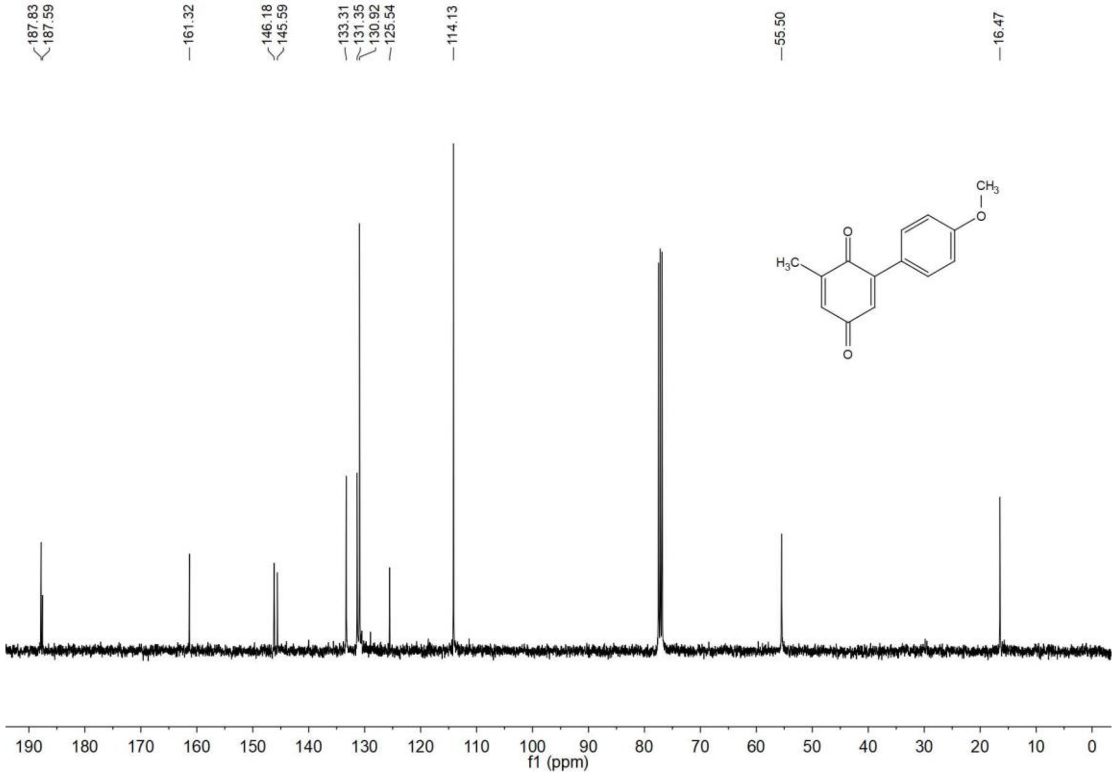

^{1}H NMR (**2c**)

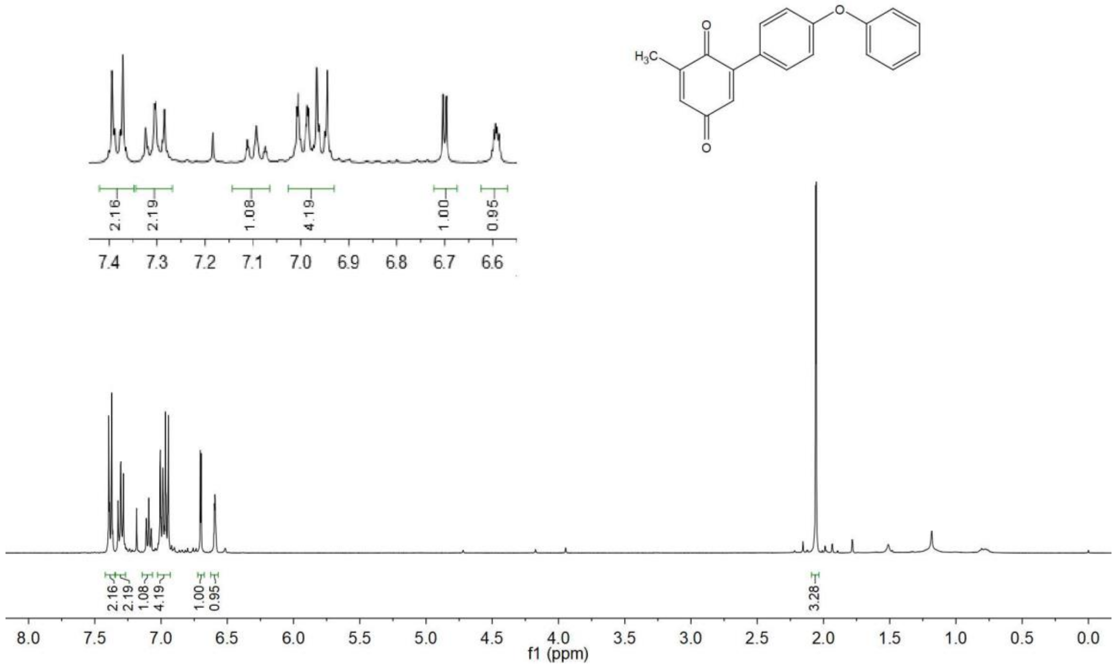

^{13}C NMR (**2c**)

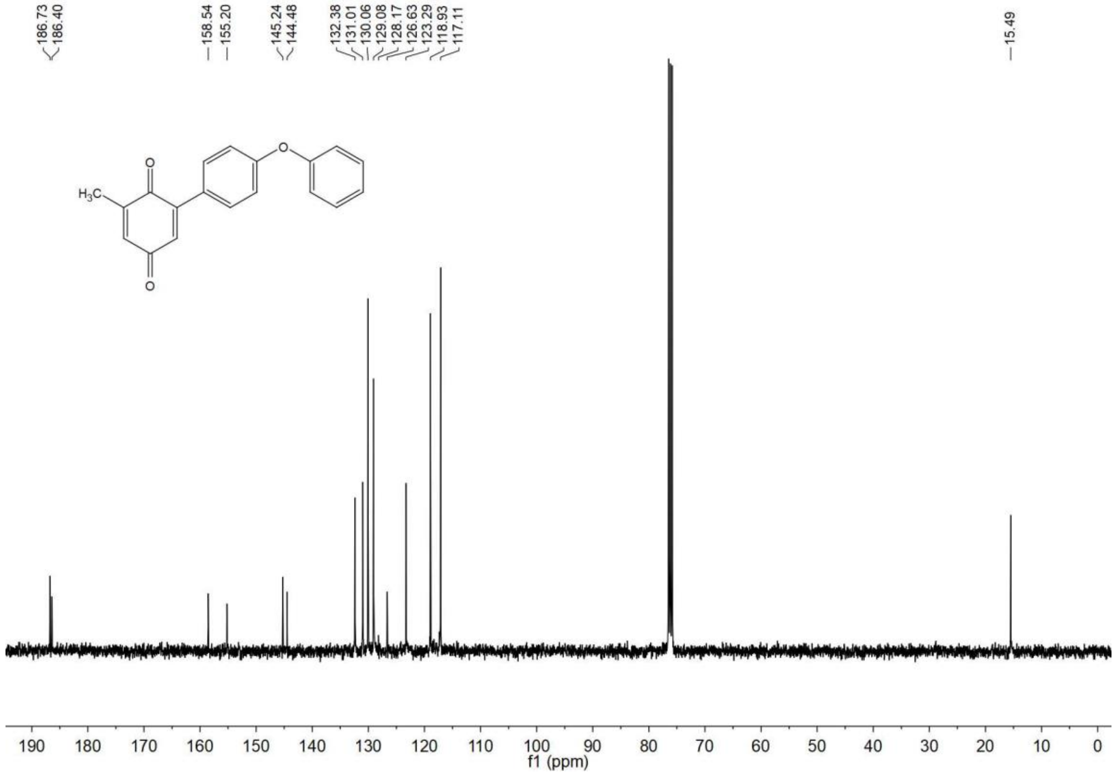

^{1}H NMR (**3c**)

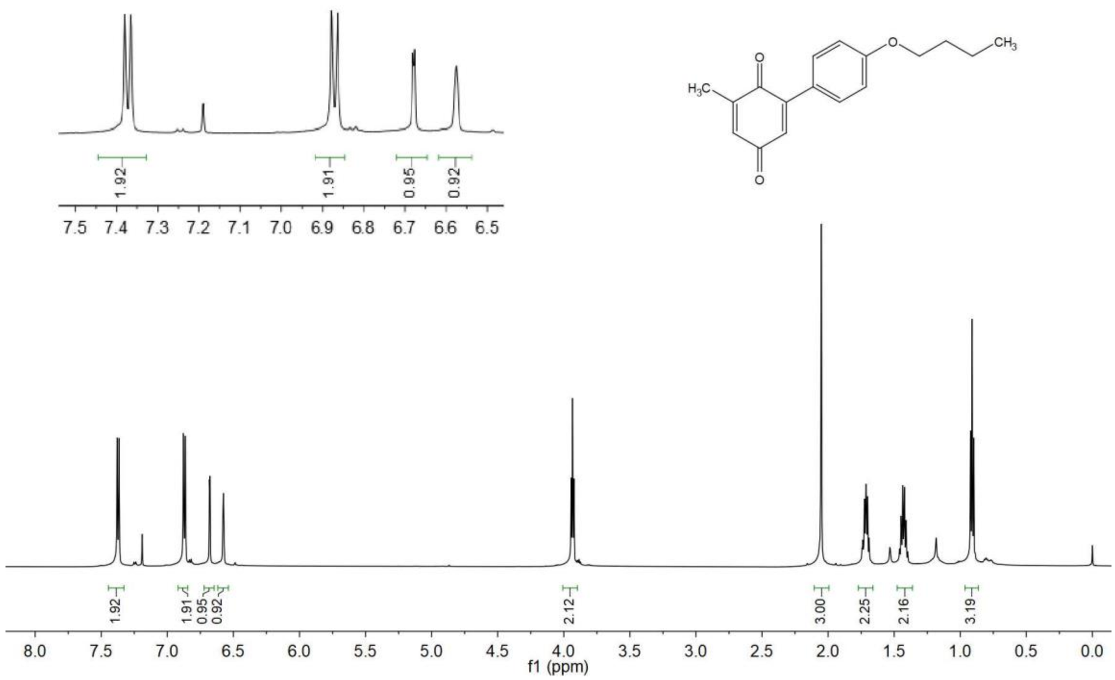

^{13}C NMR (**3c**)

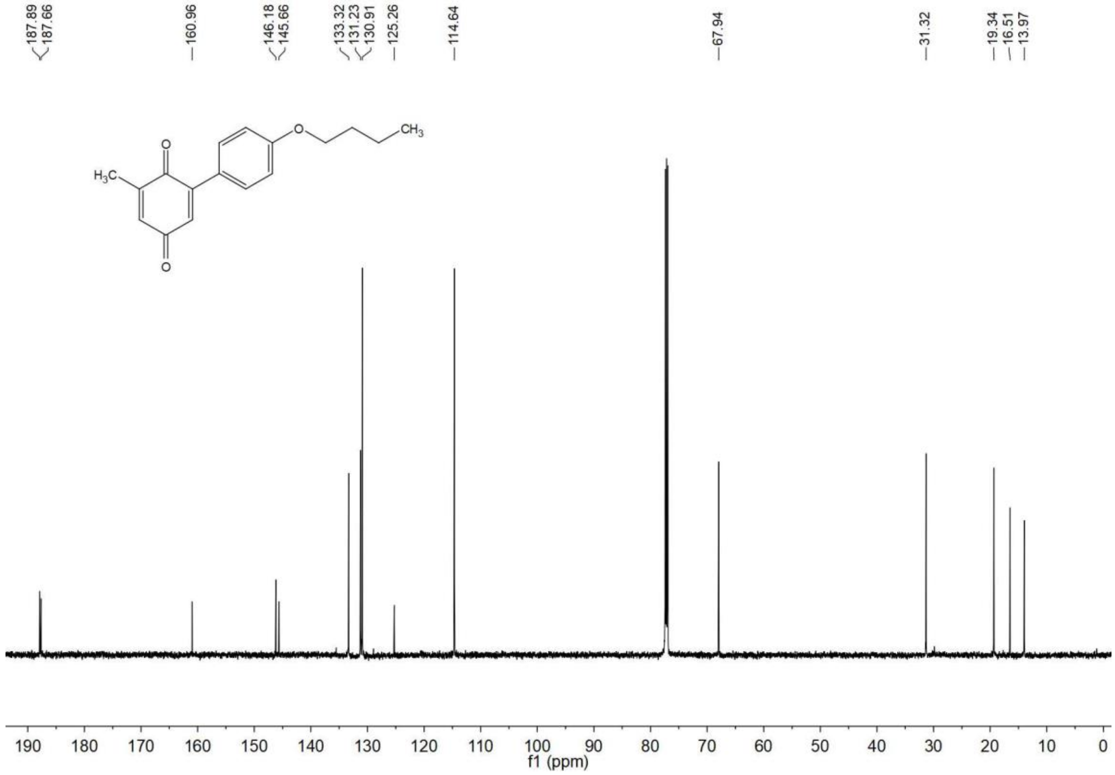

^{1}H NMR (**4c**)

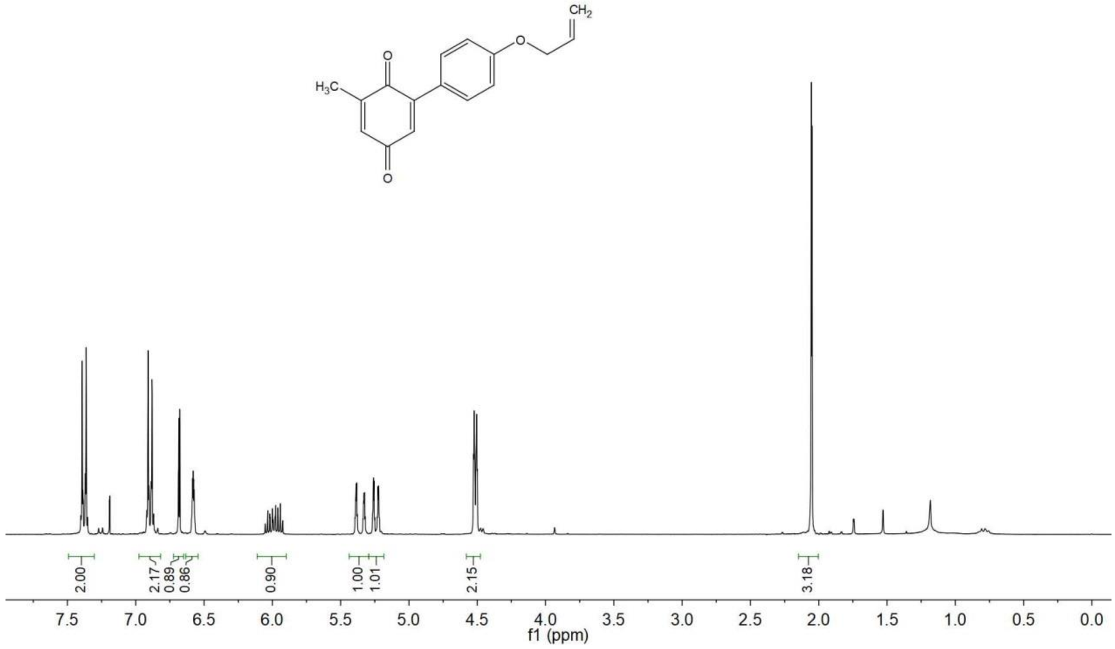

^{13}C NMR (**4c**)

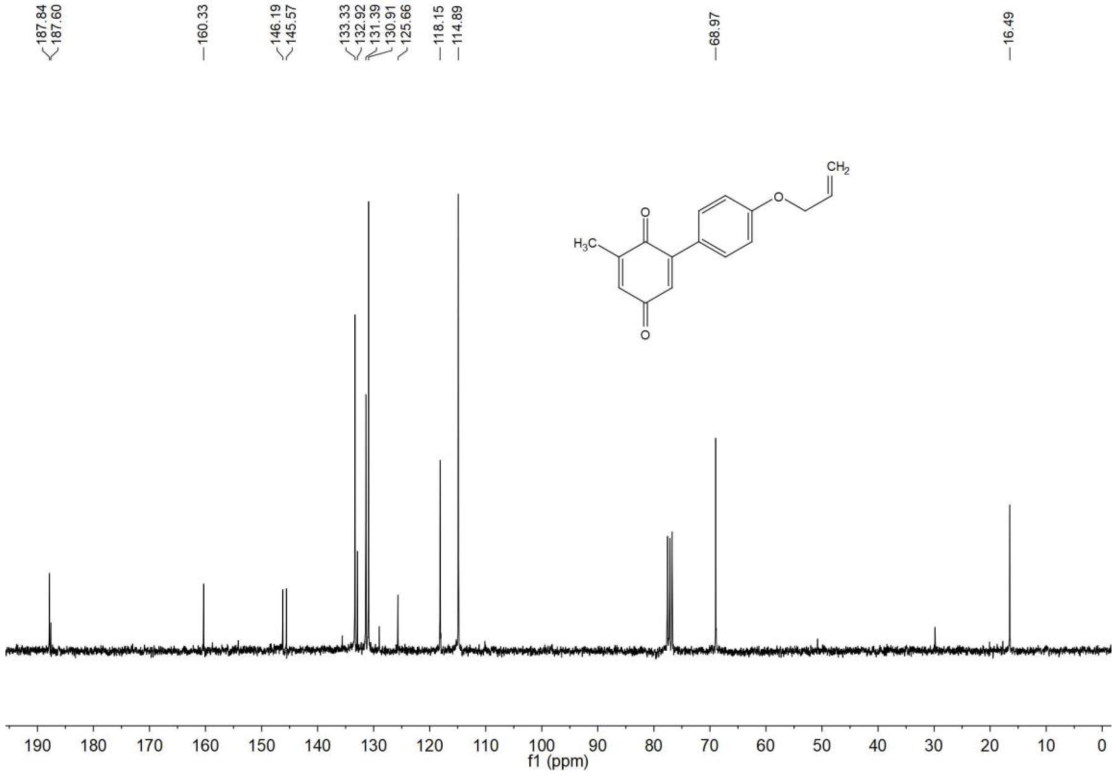

^{1}H NMR (**5c**)

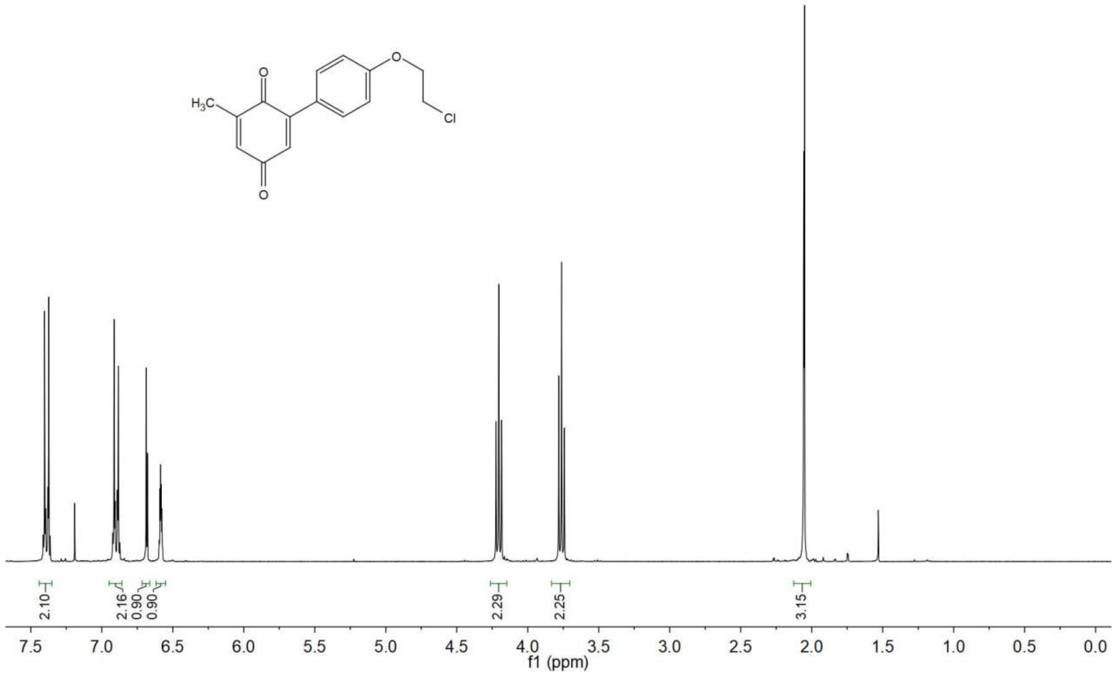

^{13}C NMR (**5c**)

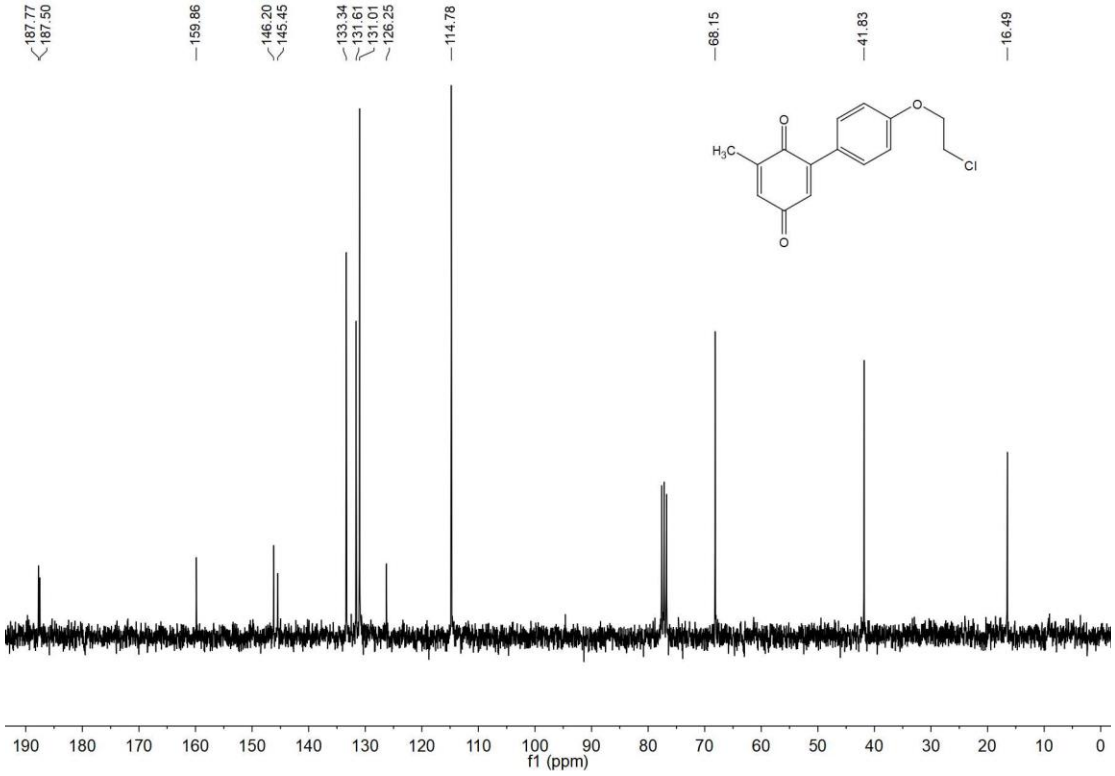

^{1}H NMR (**6c**)

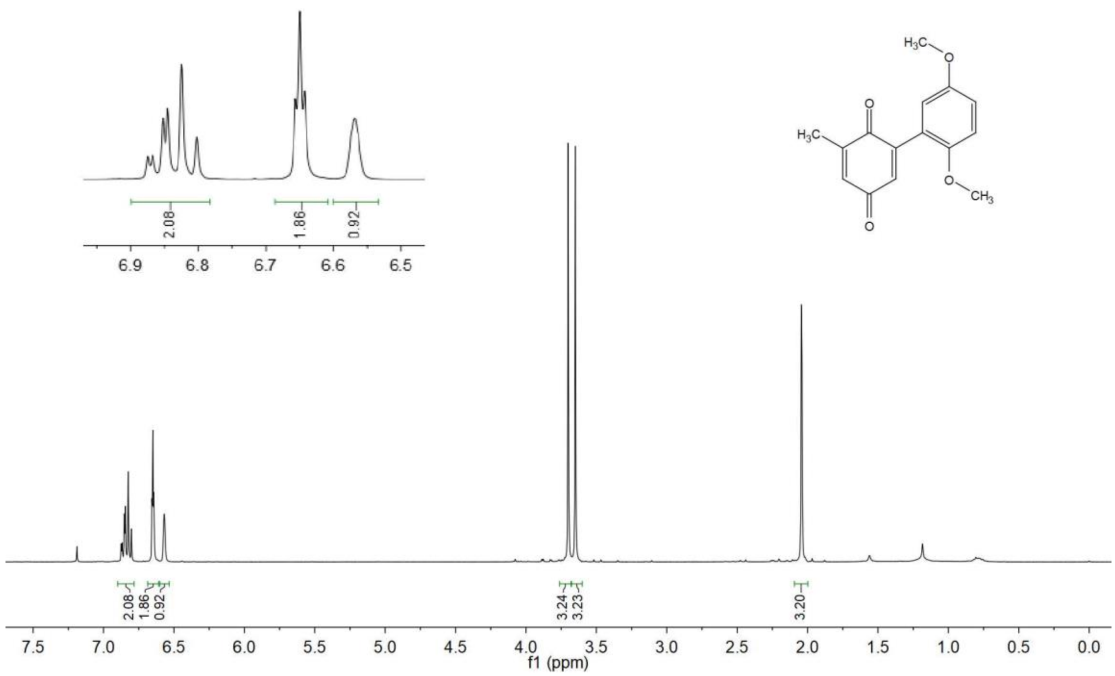

^{13}C NMR (**6c**)

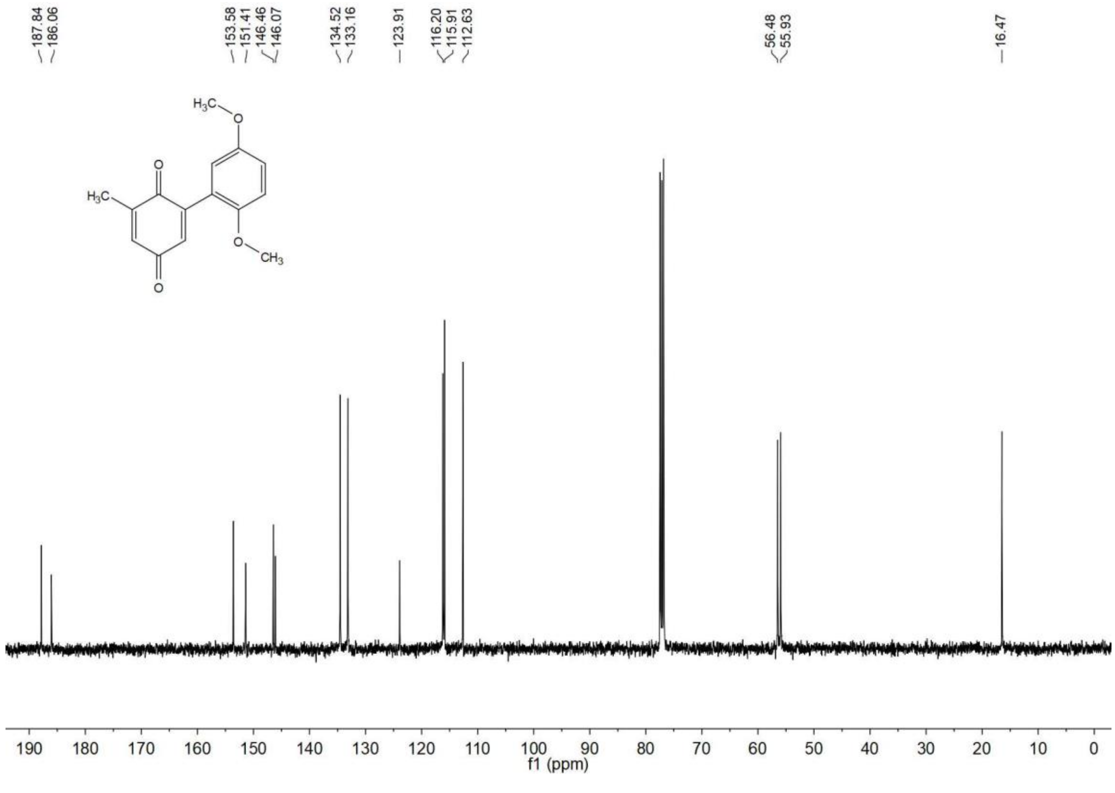

^{1}H NMR (**7c**)

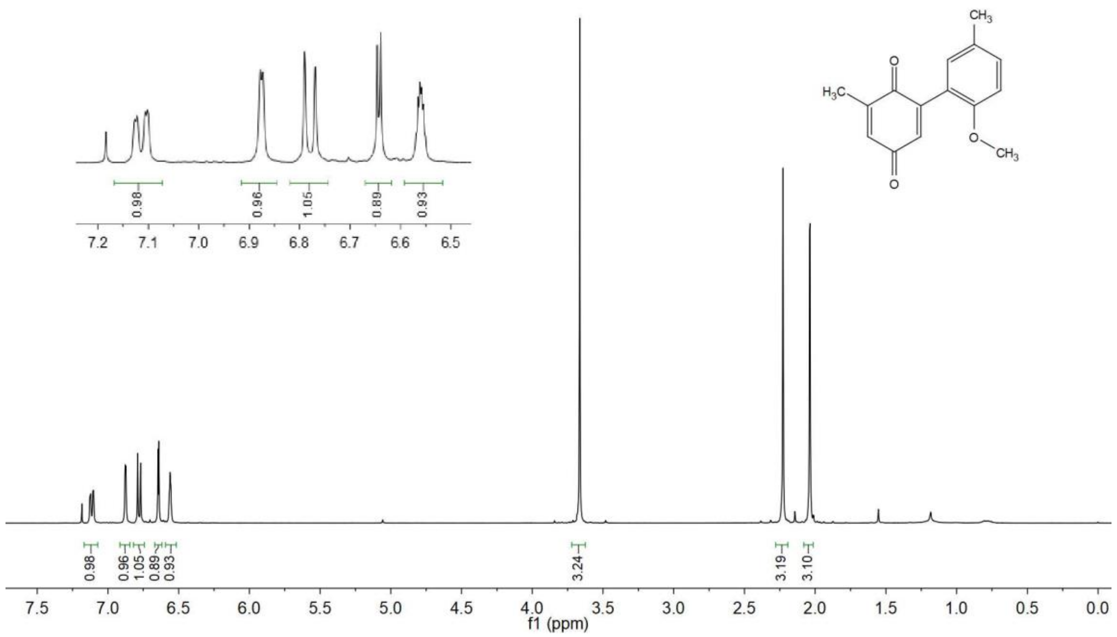

^{13}C NMR (**7c**)

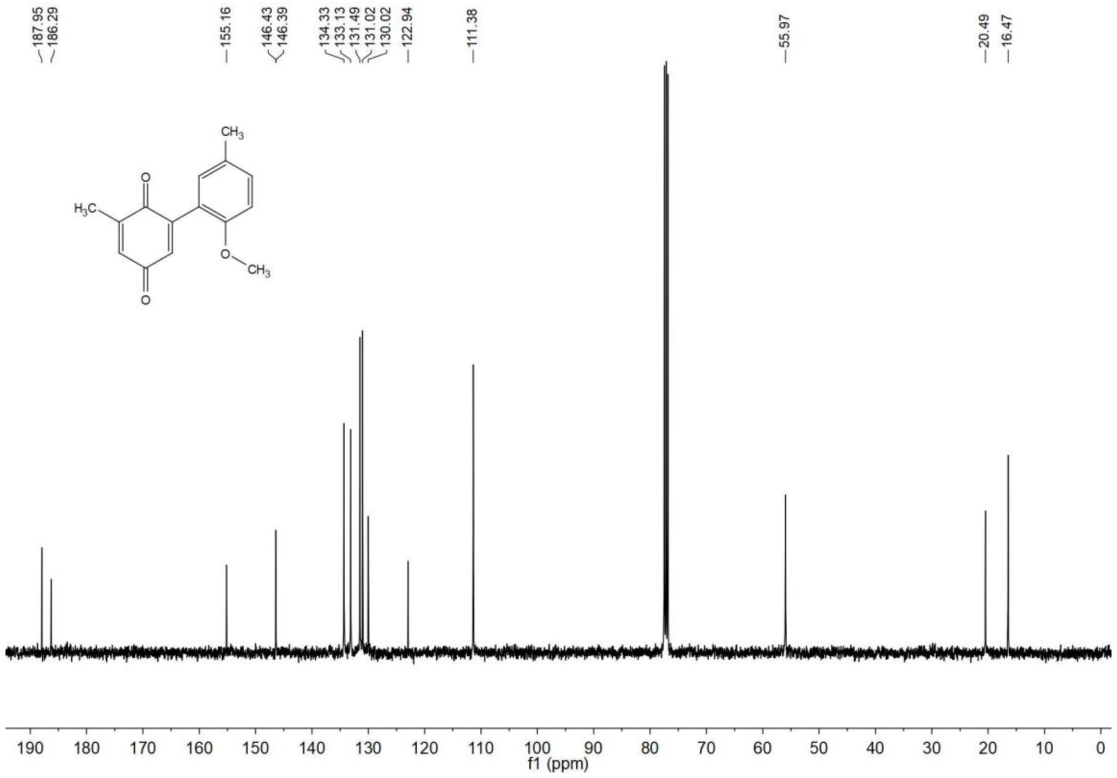

^{1}H NMR (**8c**)

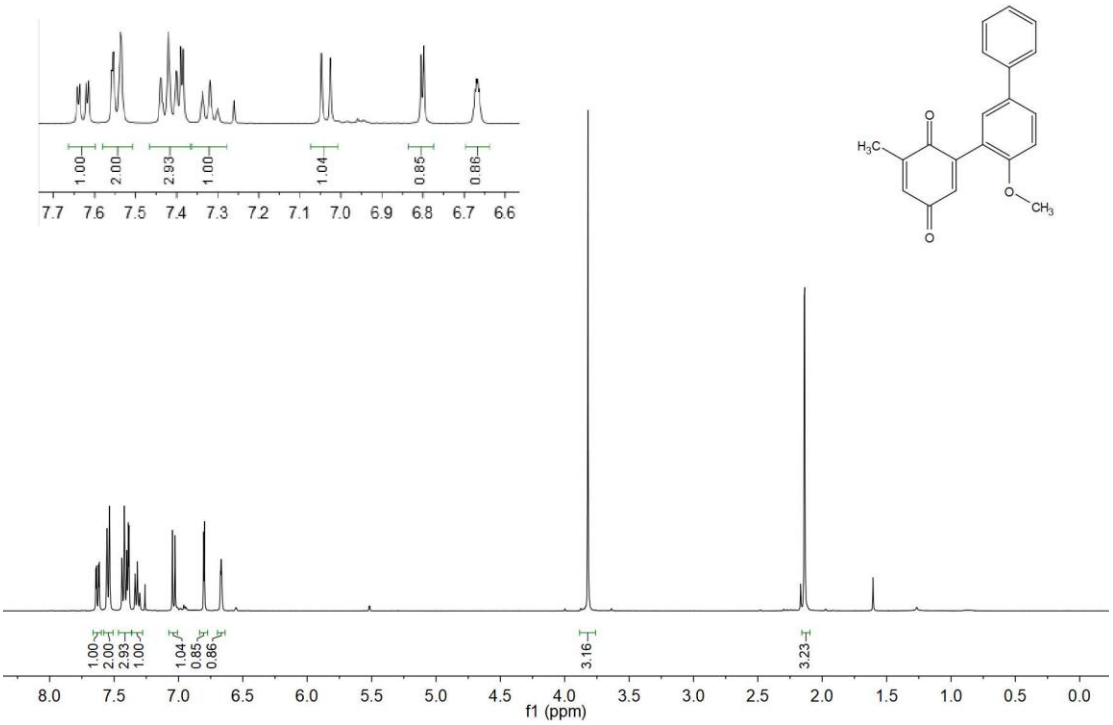

^{13}C NMR (**8c**)

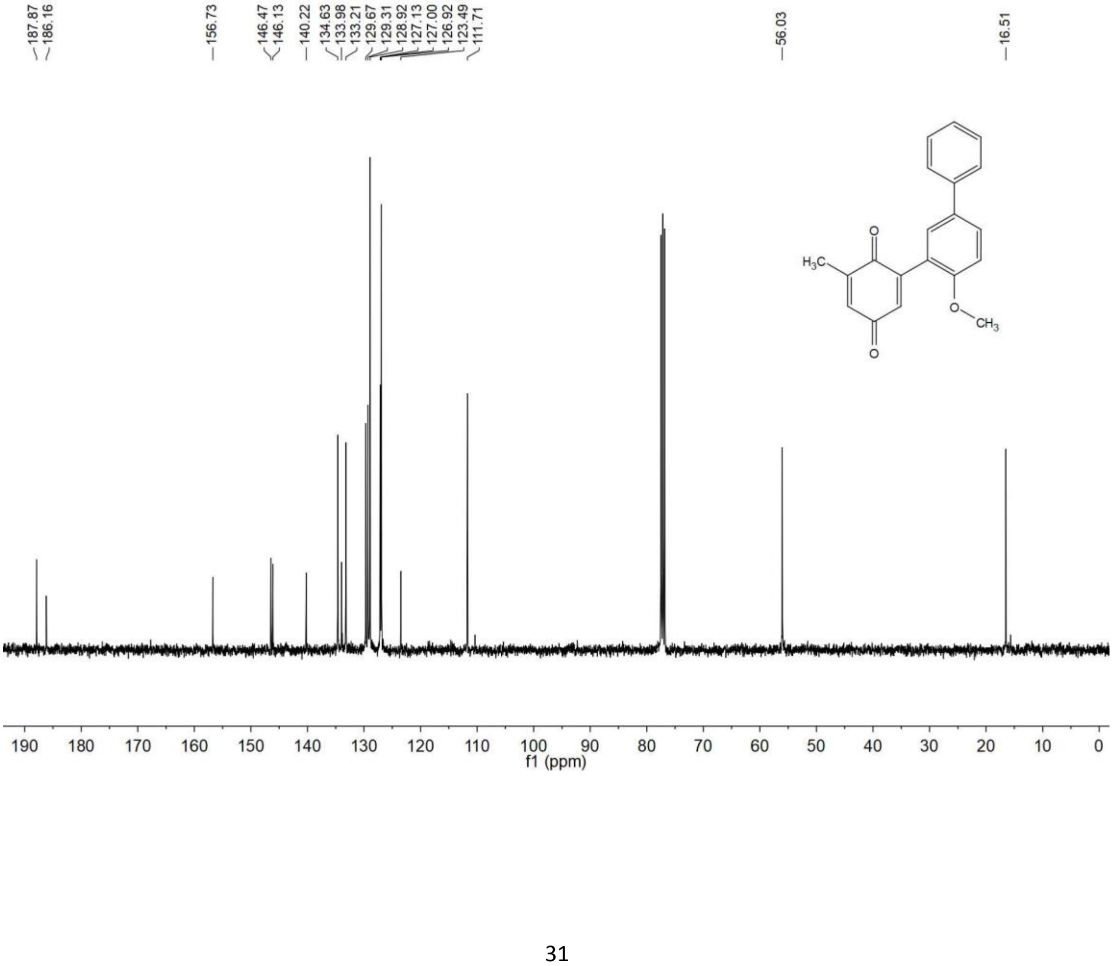

^{1}H NMR (**9c**)

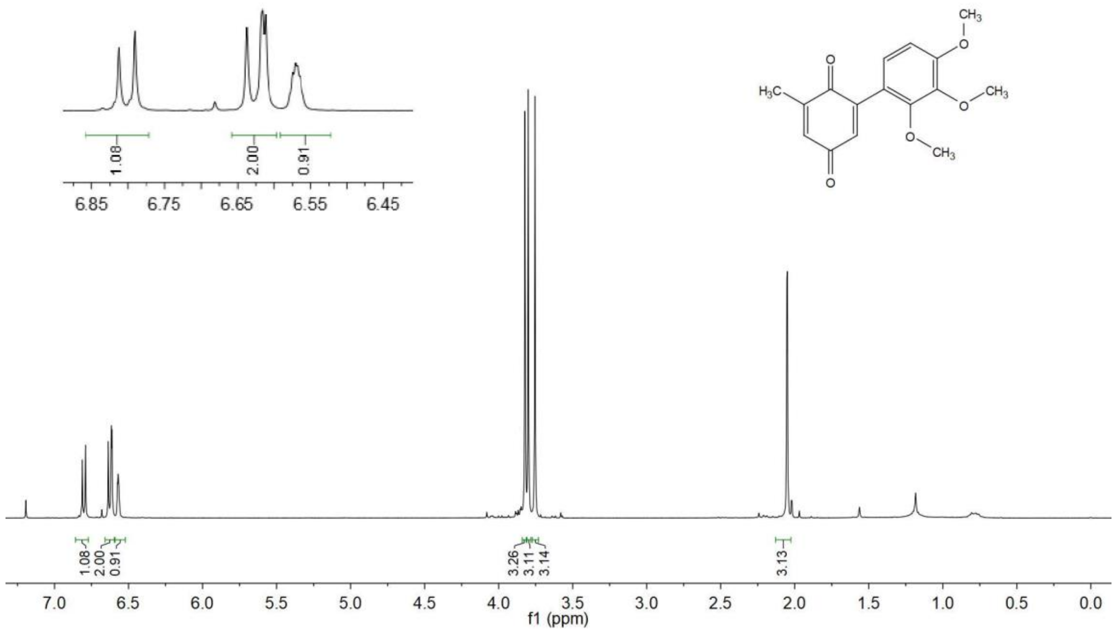

^{13}C NMR (**9c**)

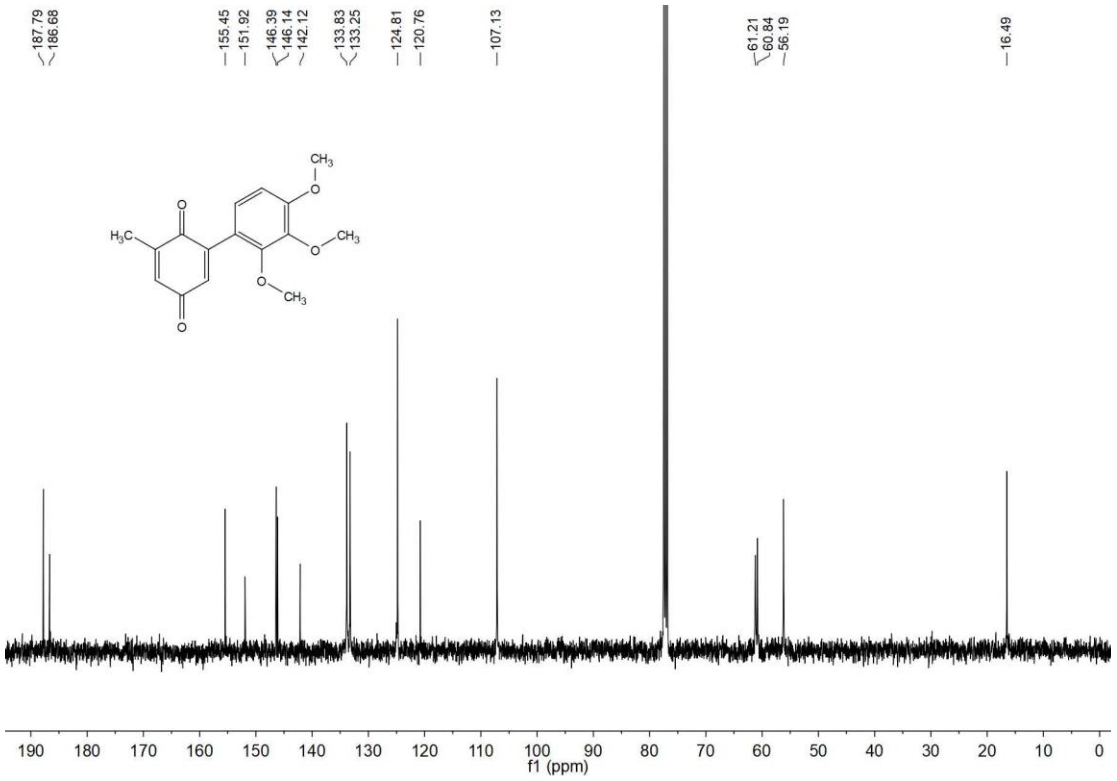

^{1}H NMR (**10c**)

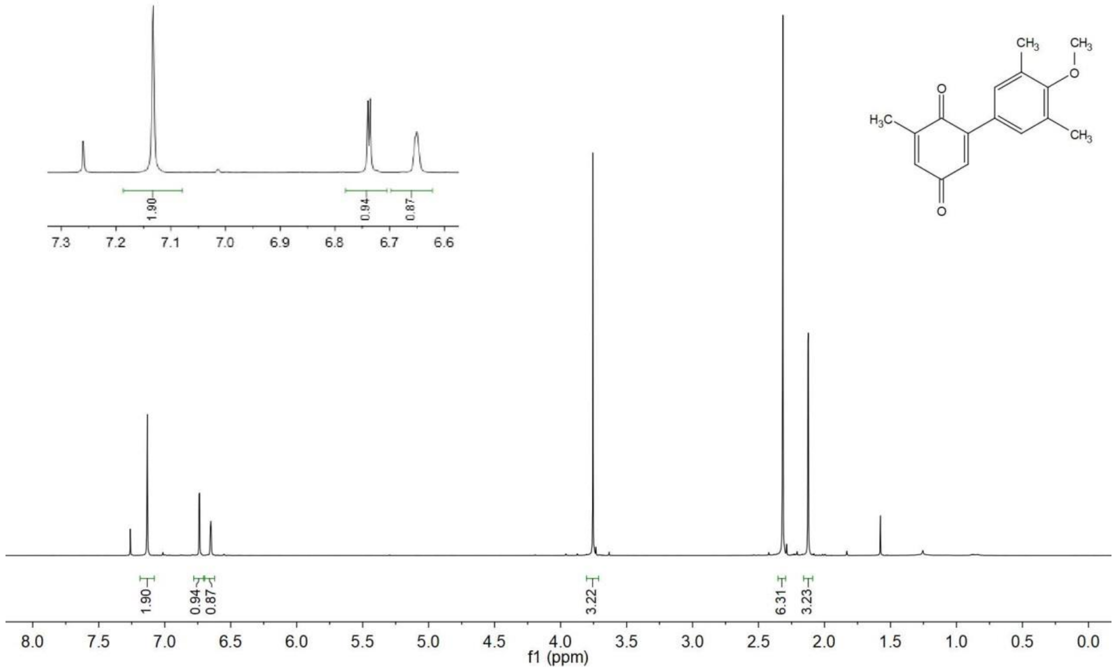

^{13}C NMR (**10c**)

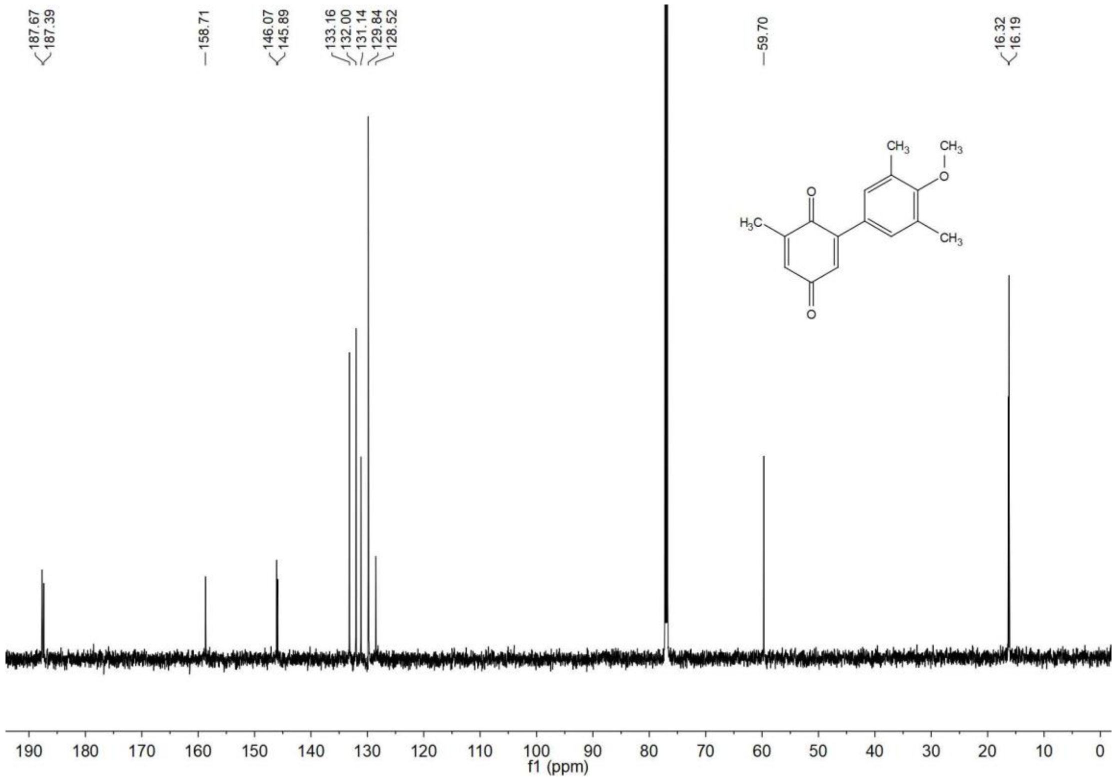

^{1}H NMR (**11c**)

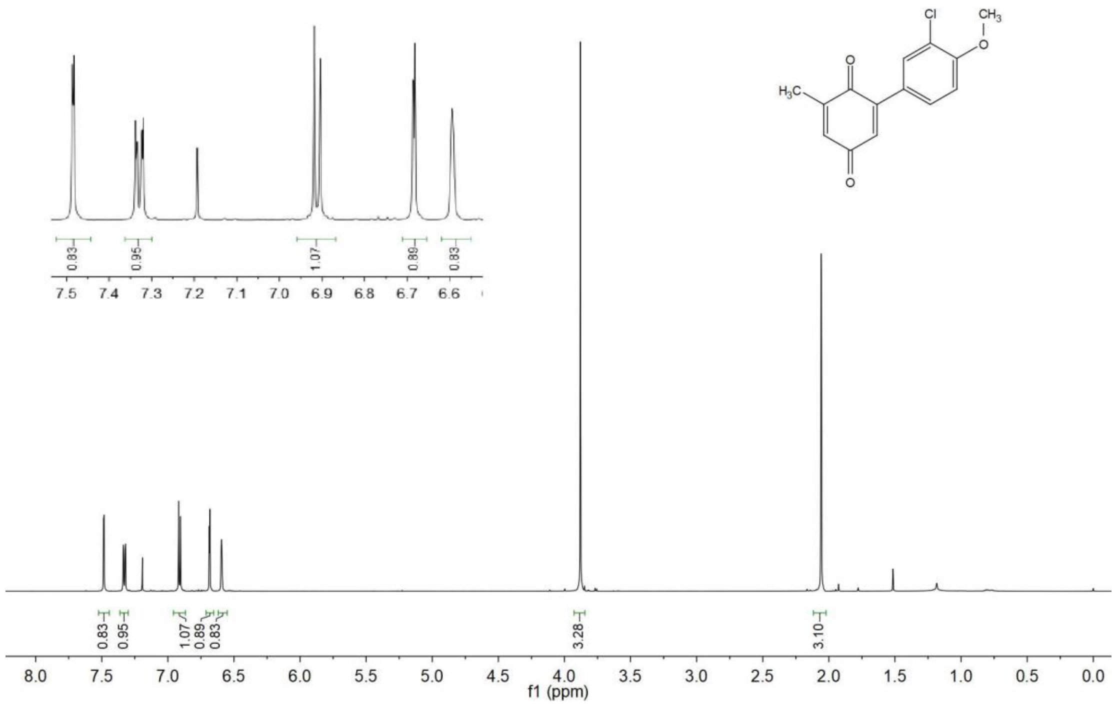

^{13}C NMR (**11c**)

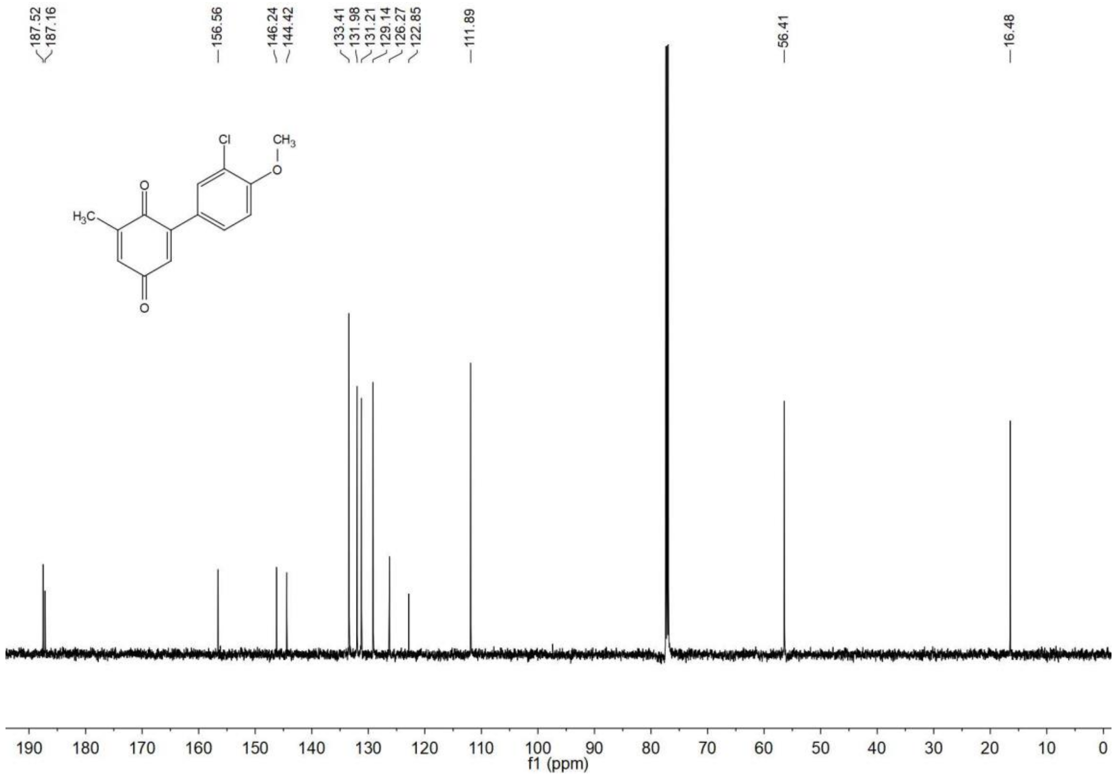

^{1}H NMR (**12c**)

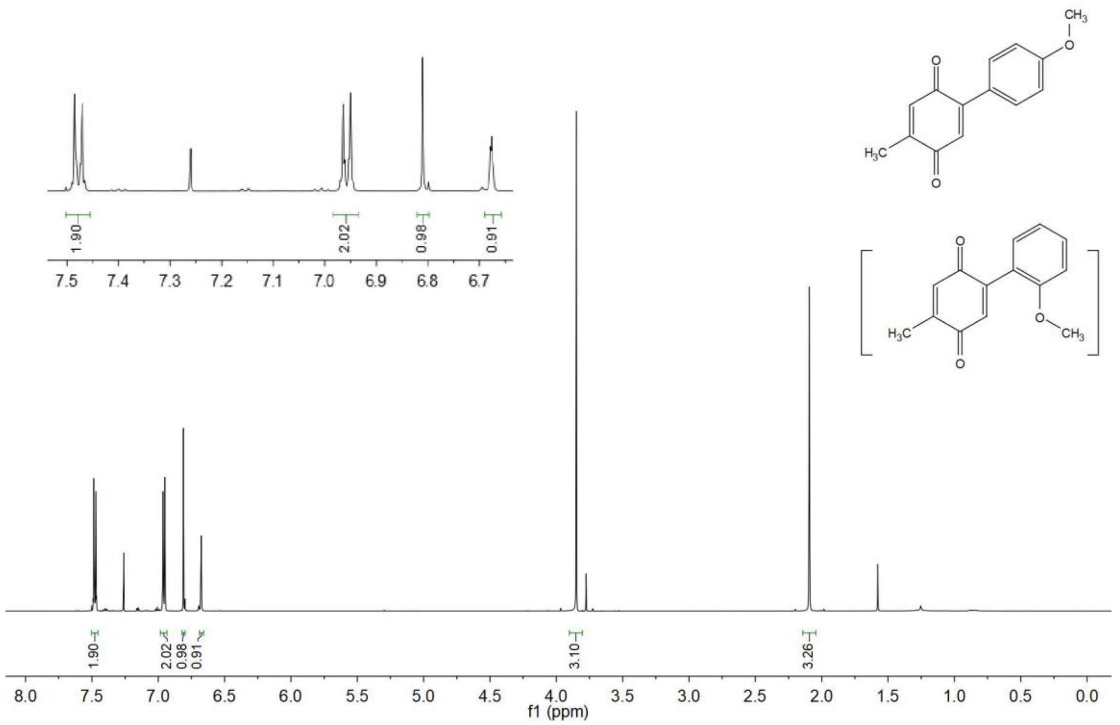

^{13}C NMR (**12c**)

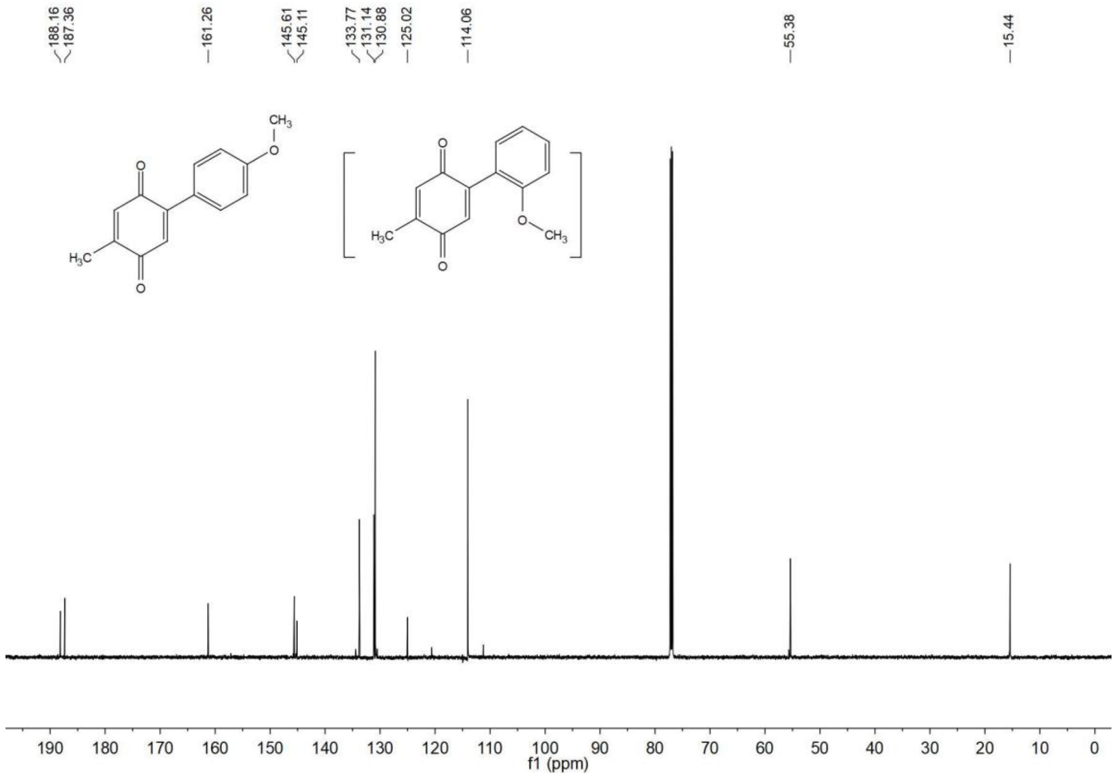

^{1}H NMR (**13c**)

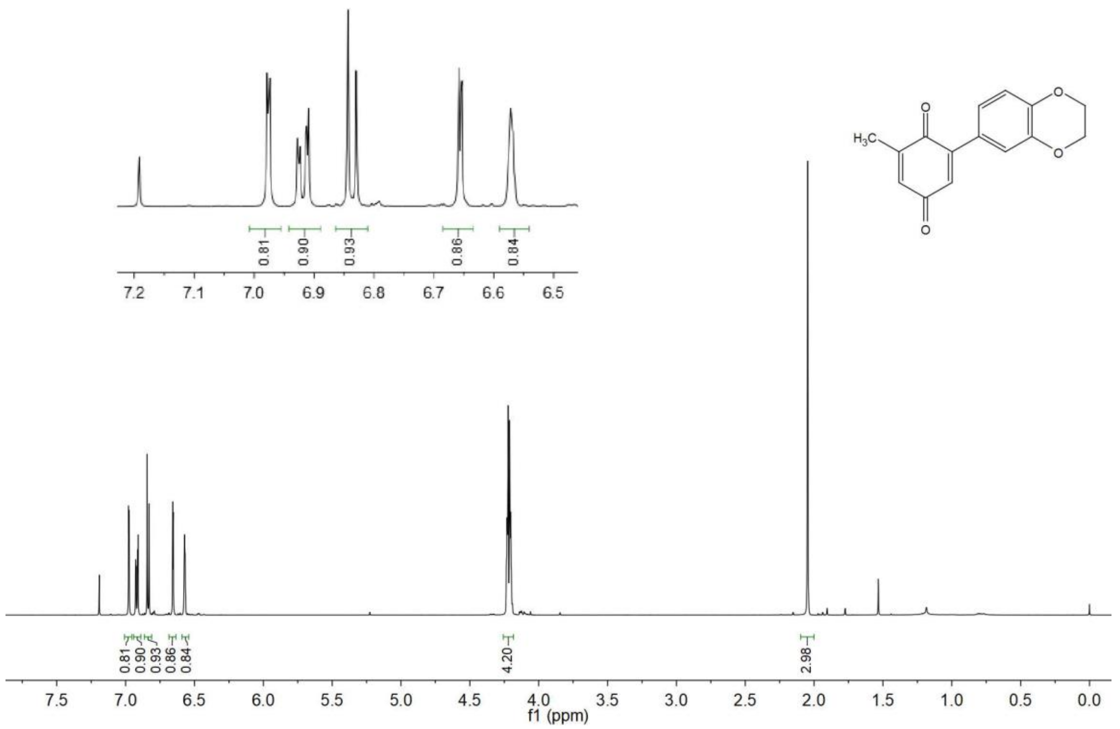

^{13}C NMR (**13c**)

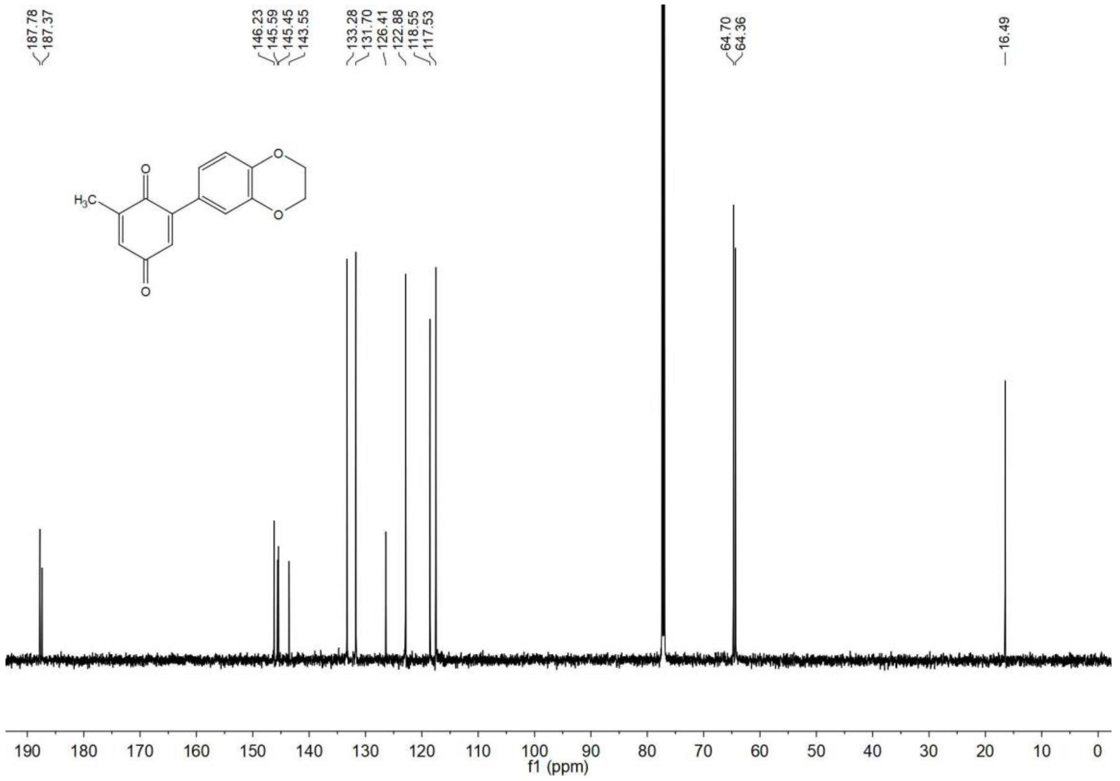

^{1}H NMR (**14c**)

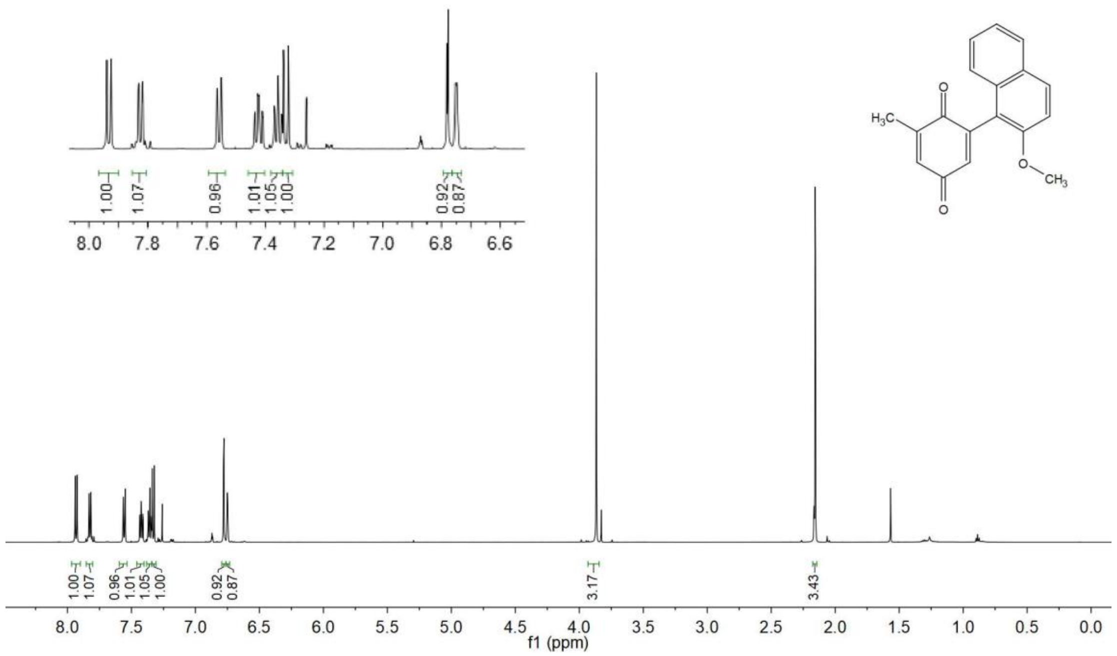

^{13}C NMR (**14c**)

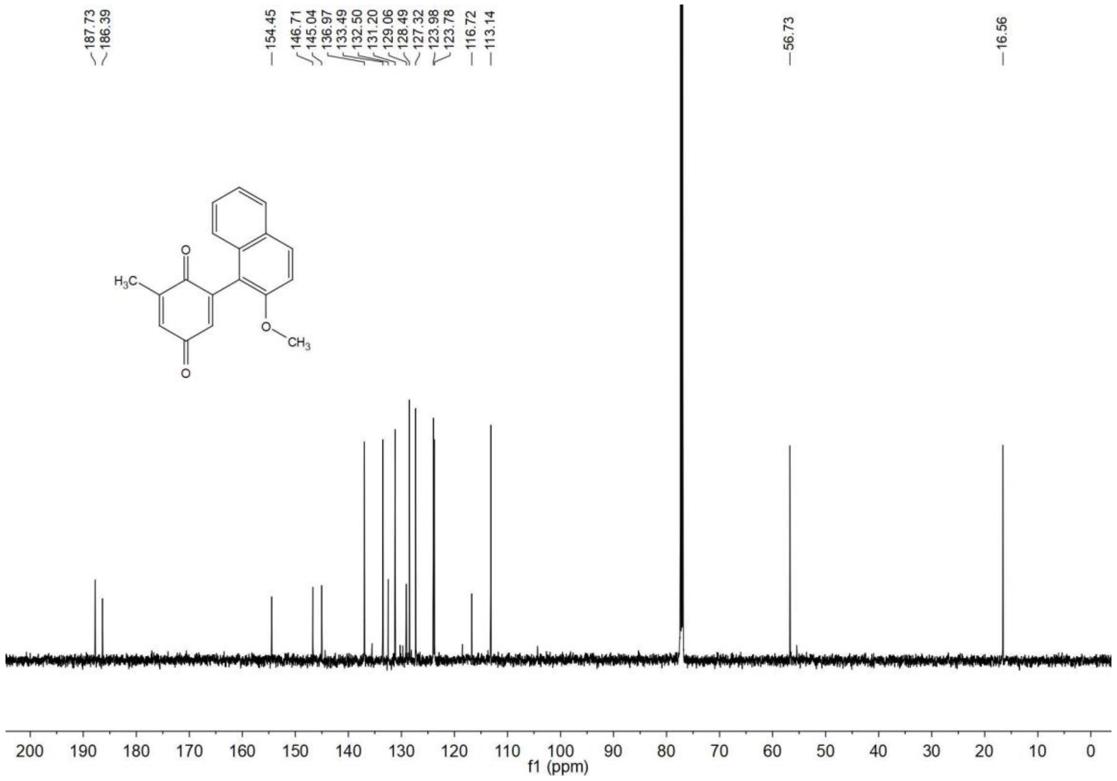

^{1}H NMR (**15c**)

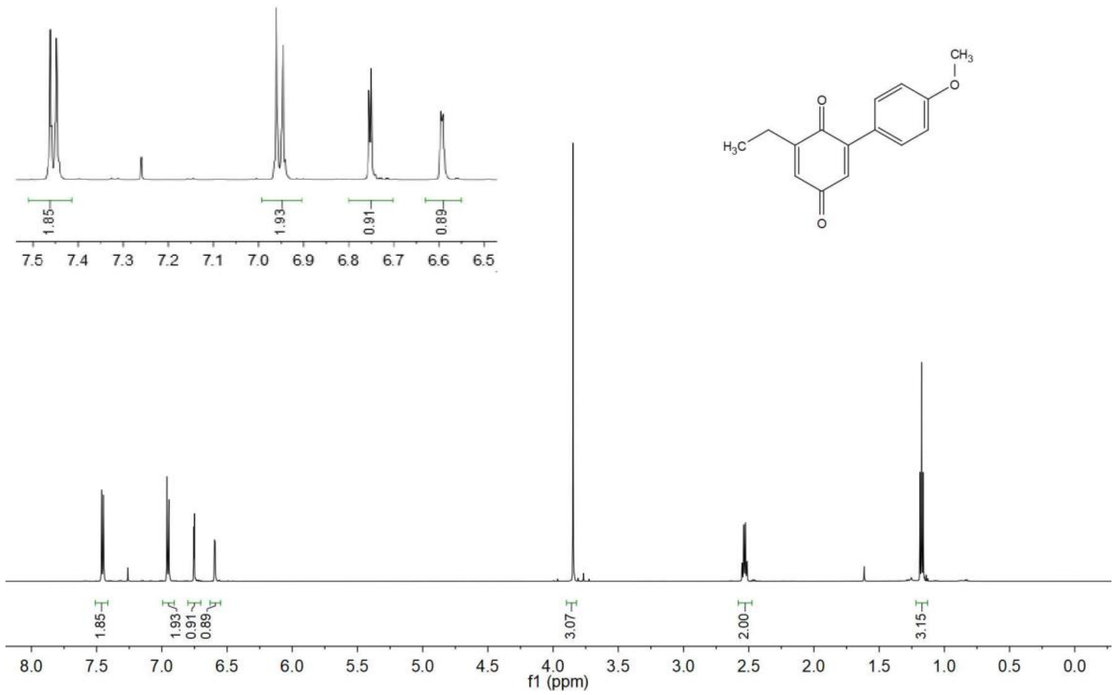

^{13}C NMR (**15c**)

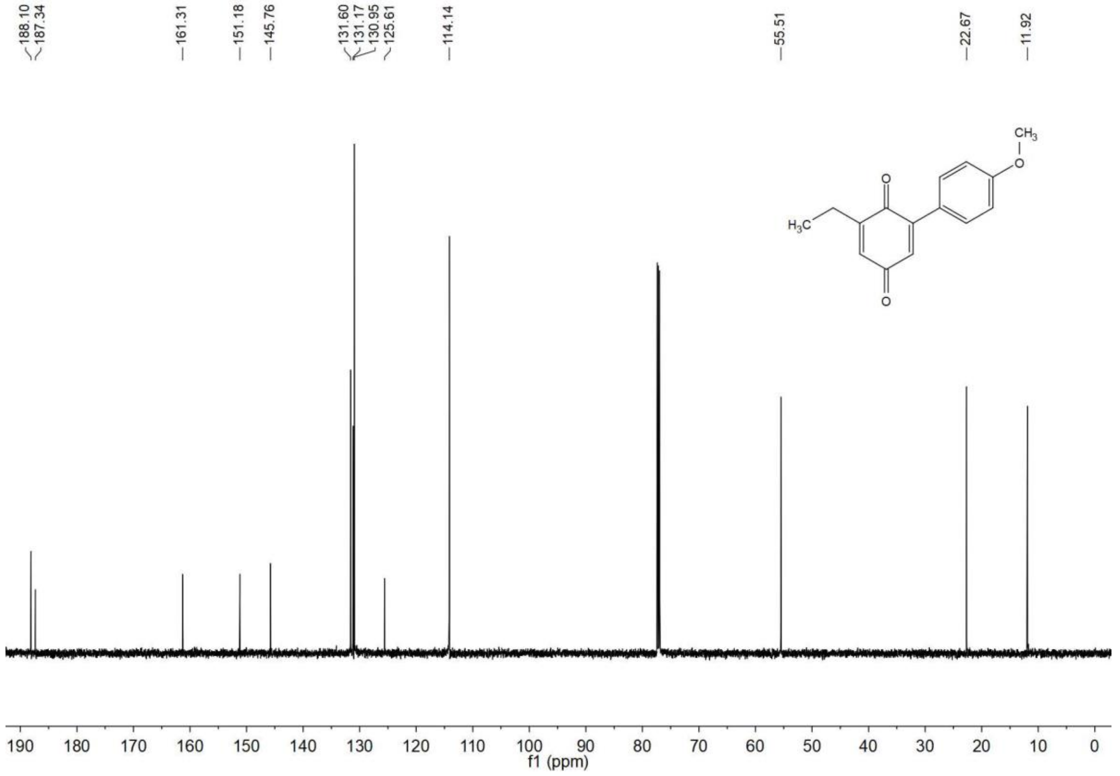

^{1}H NMR (**16c**)

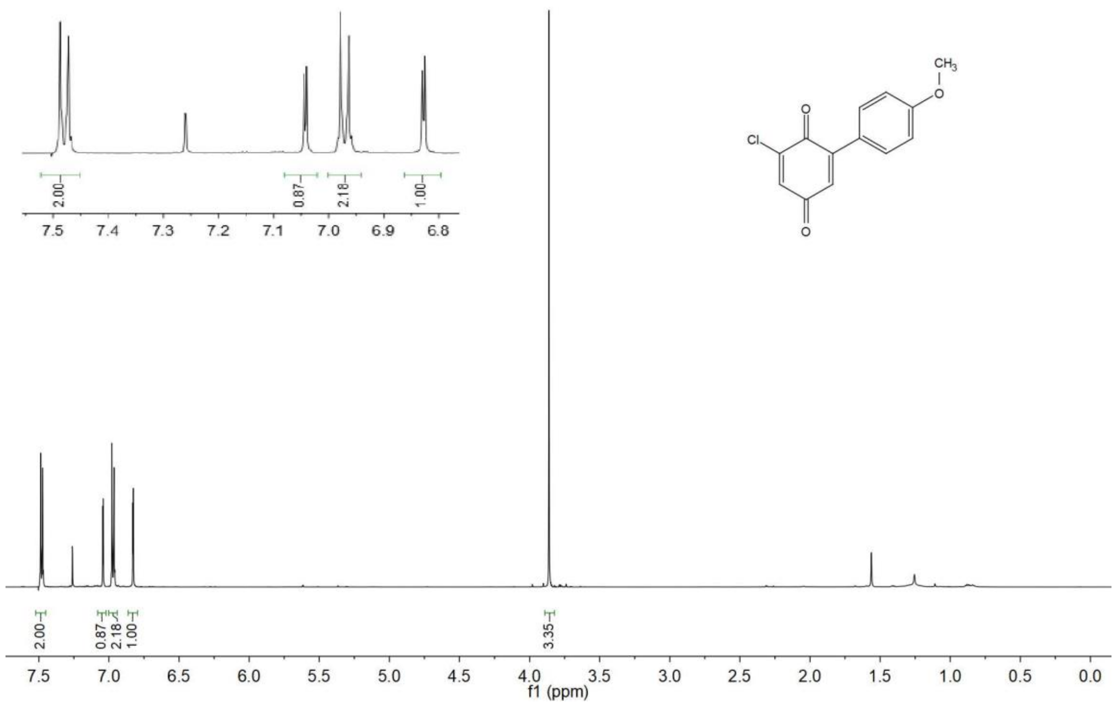

^{13}C NMR (**16c**)

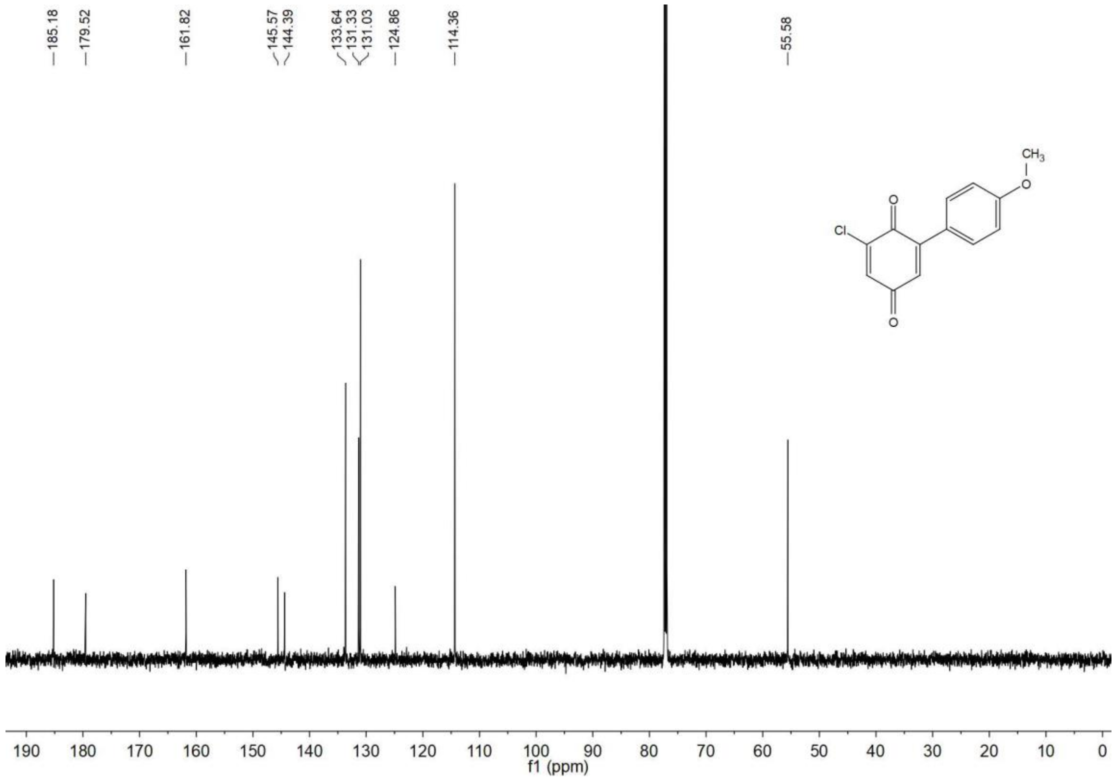

^{1}H NMR (**17c**)

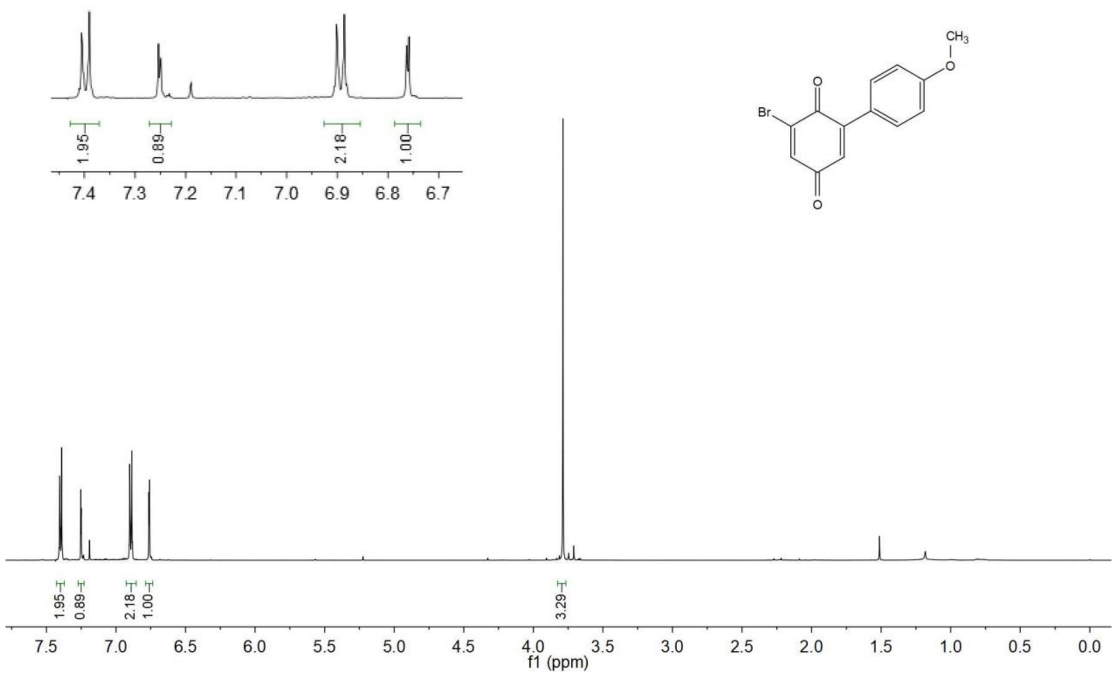

^{13}C NMR (**17c**)

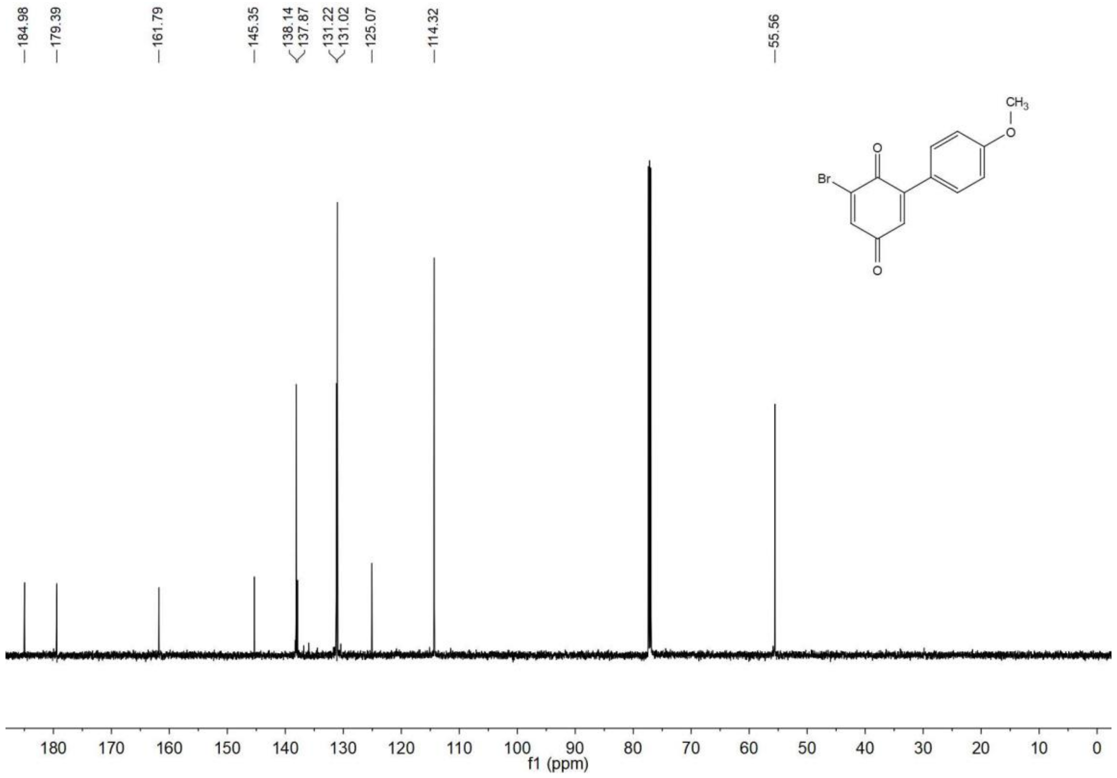

^{1}H NMR (**18c**)

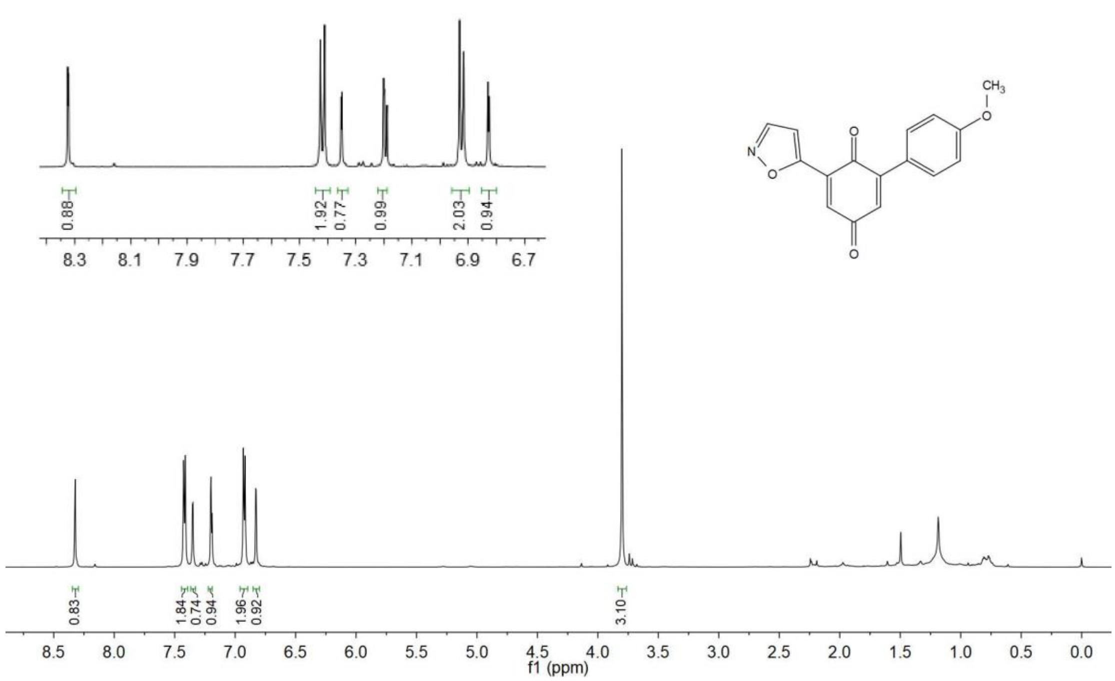

^{13}C NMR (**18c**)

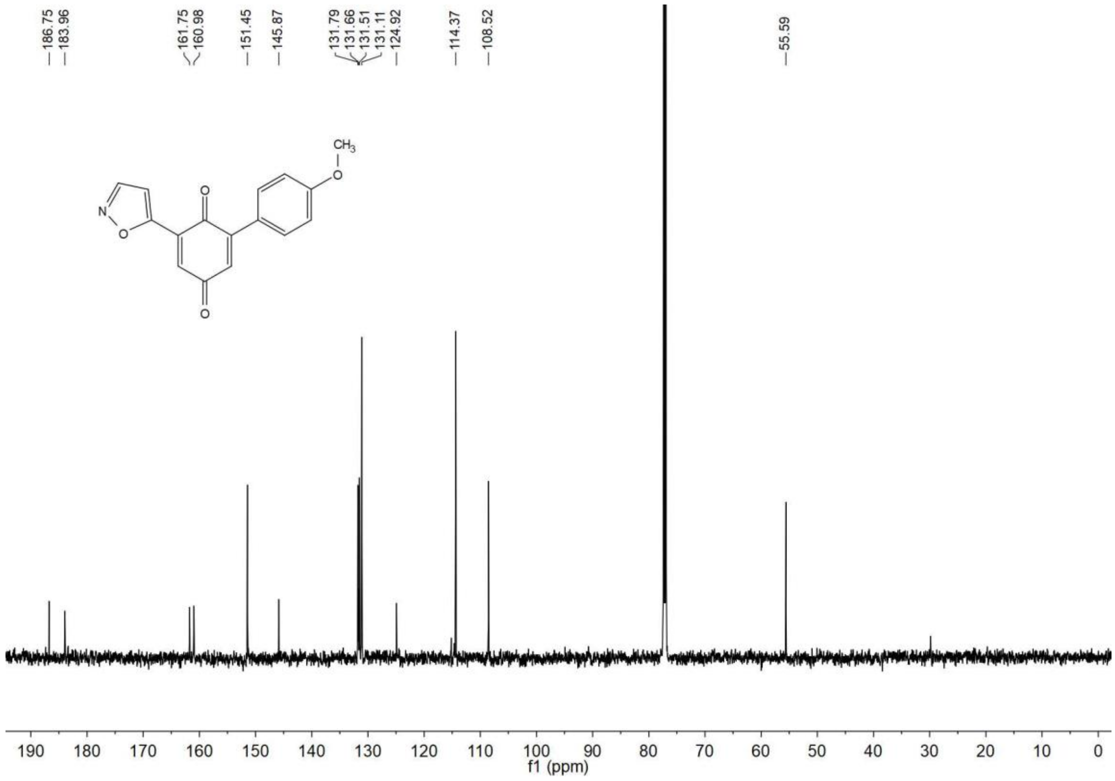

^{1}H NMR (**19c**)

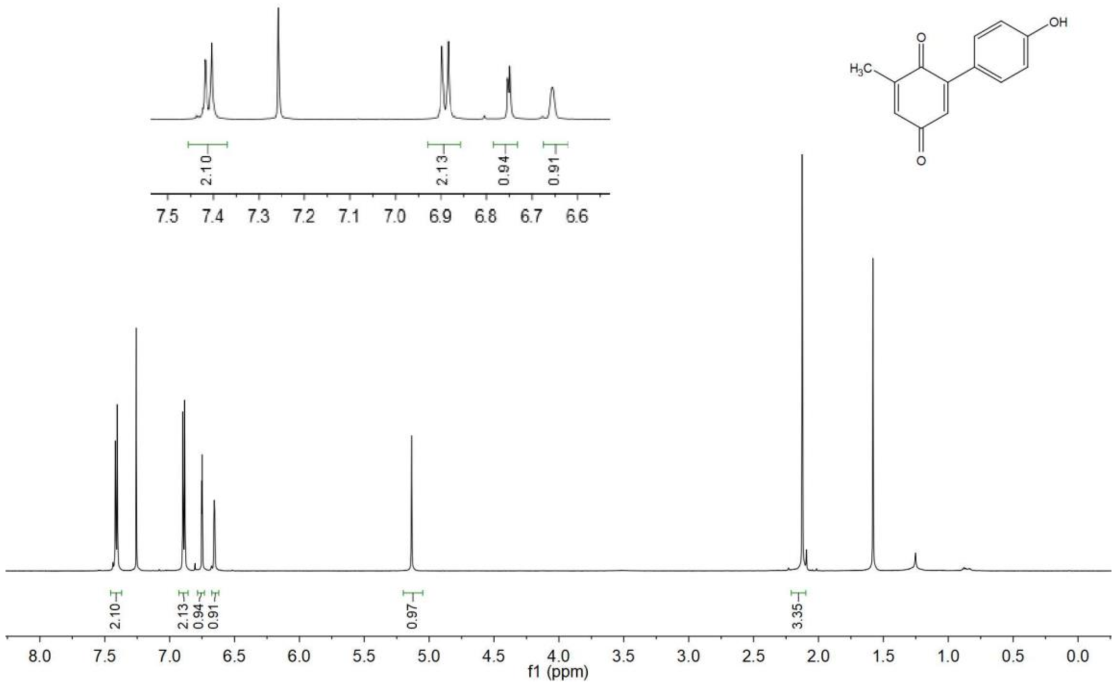

^{13}C NMR (**19c**)

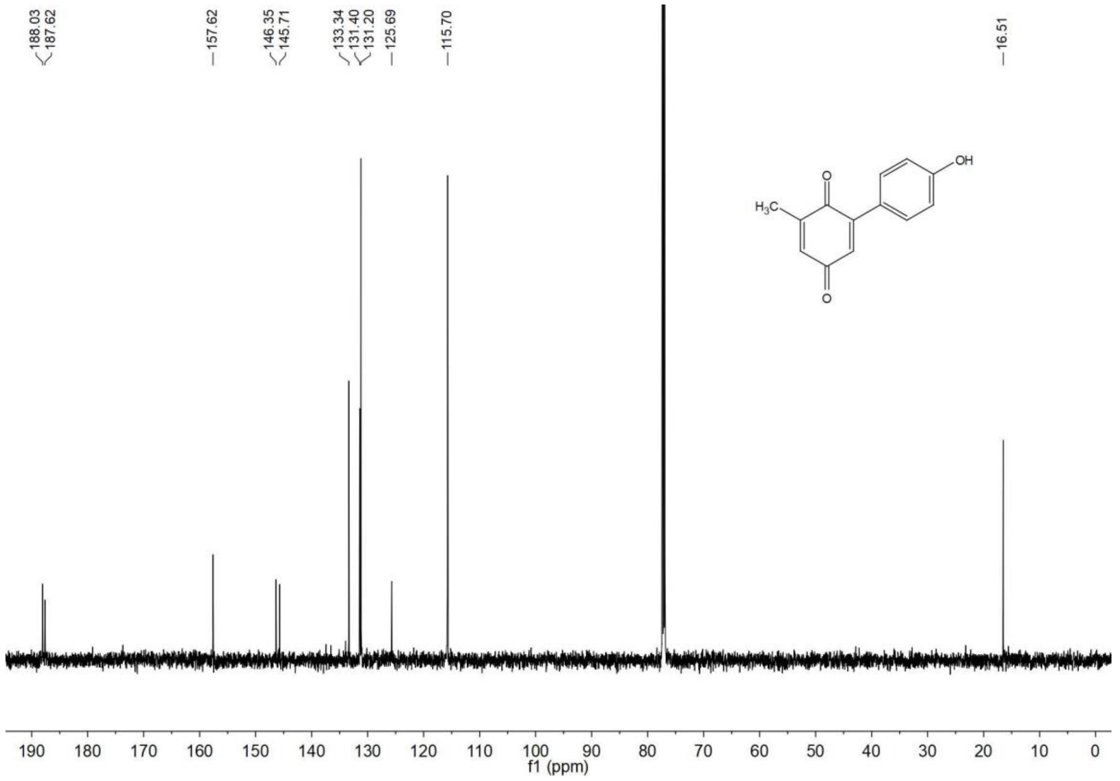

^{1}H NMR (**20c**)

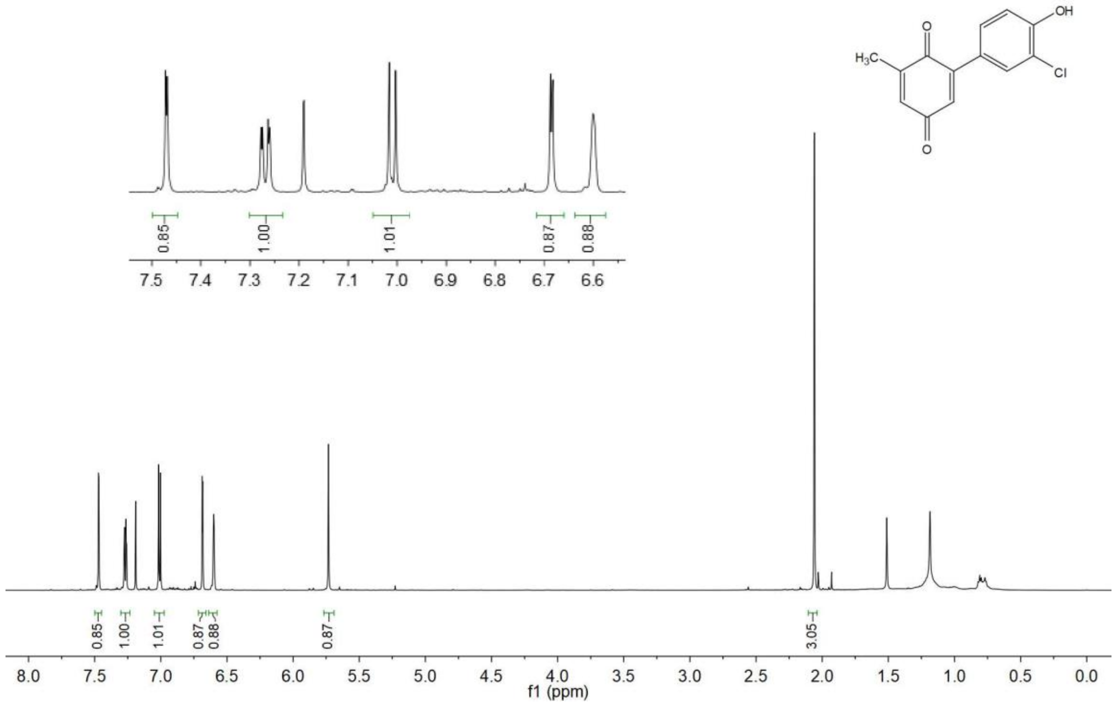

^{13}C NMR (**20c**)

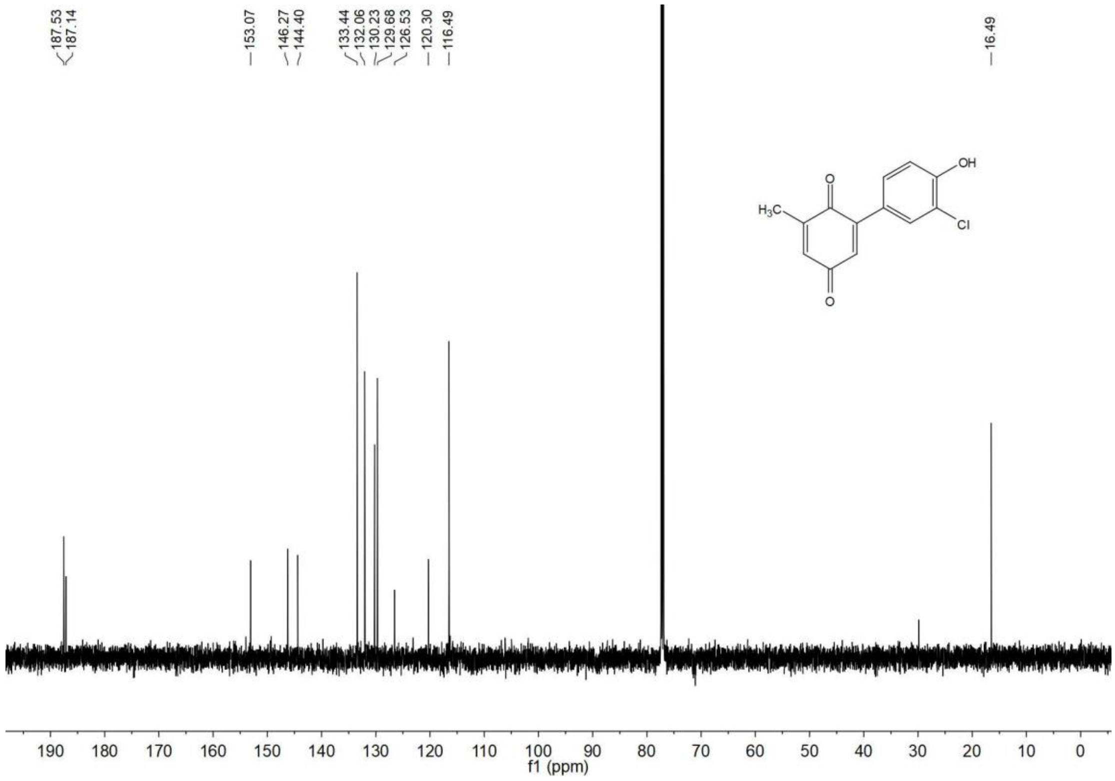

^{1}H NMR (**21c**)

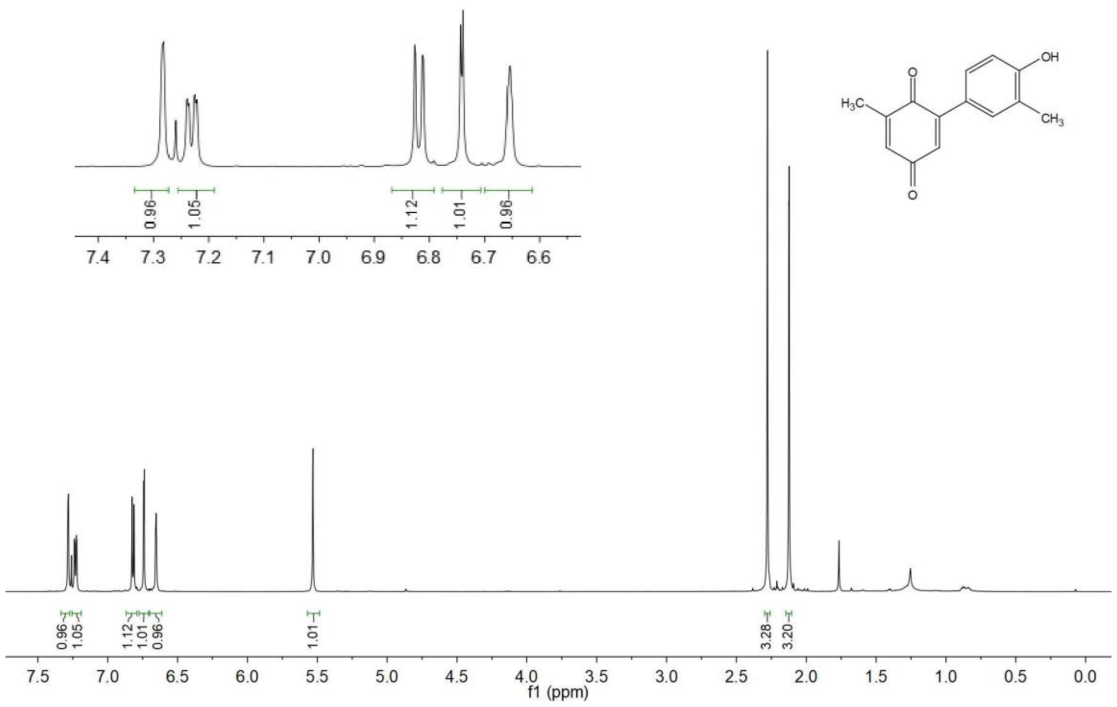

^{13}C NMR (**21c**)

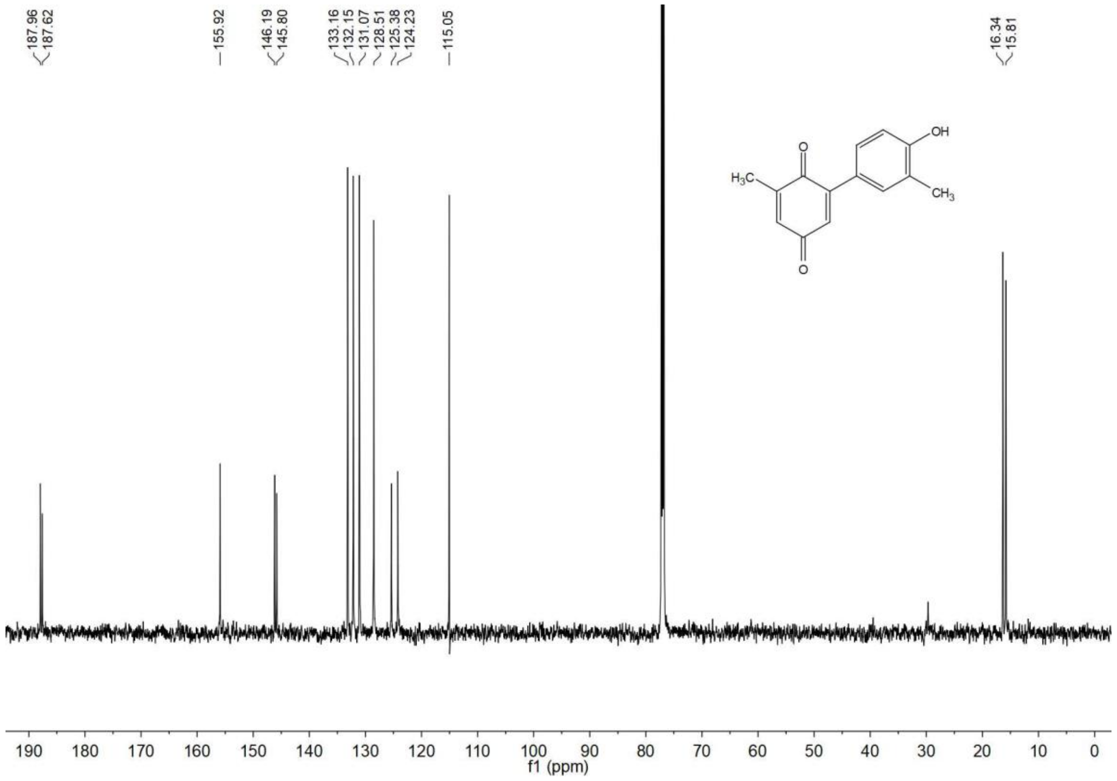

^{1}H NMR (**1e**)

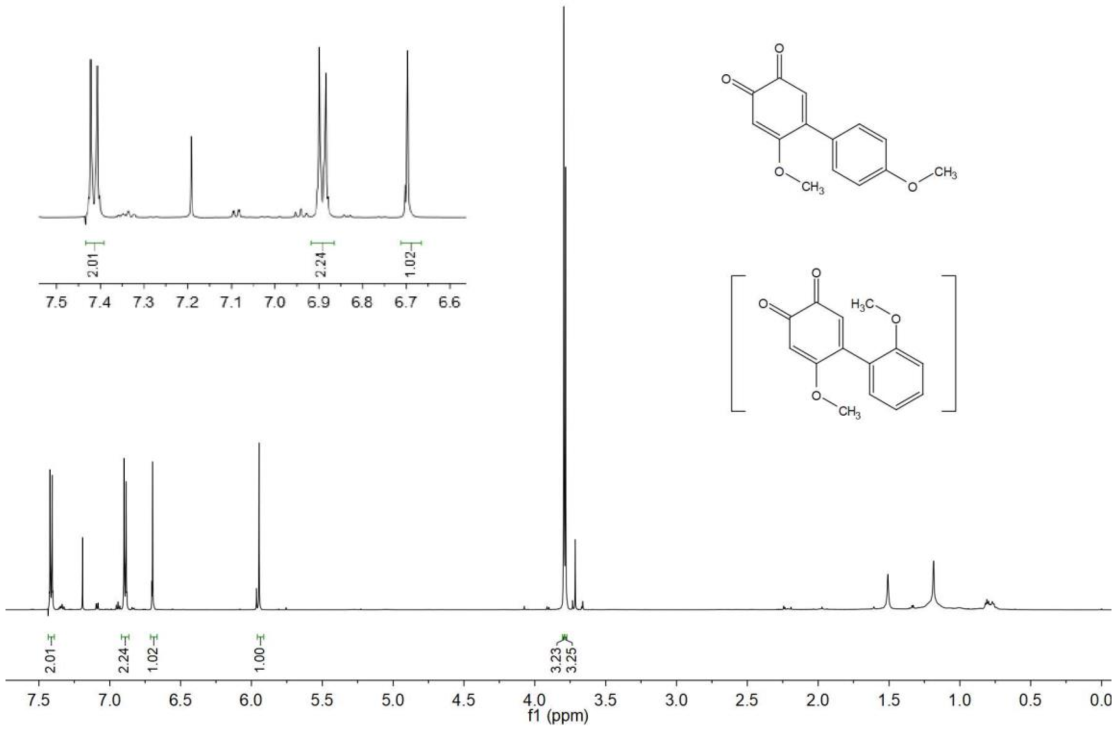

^{13}C NMR (**1e**)

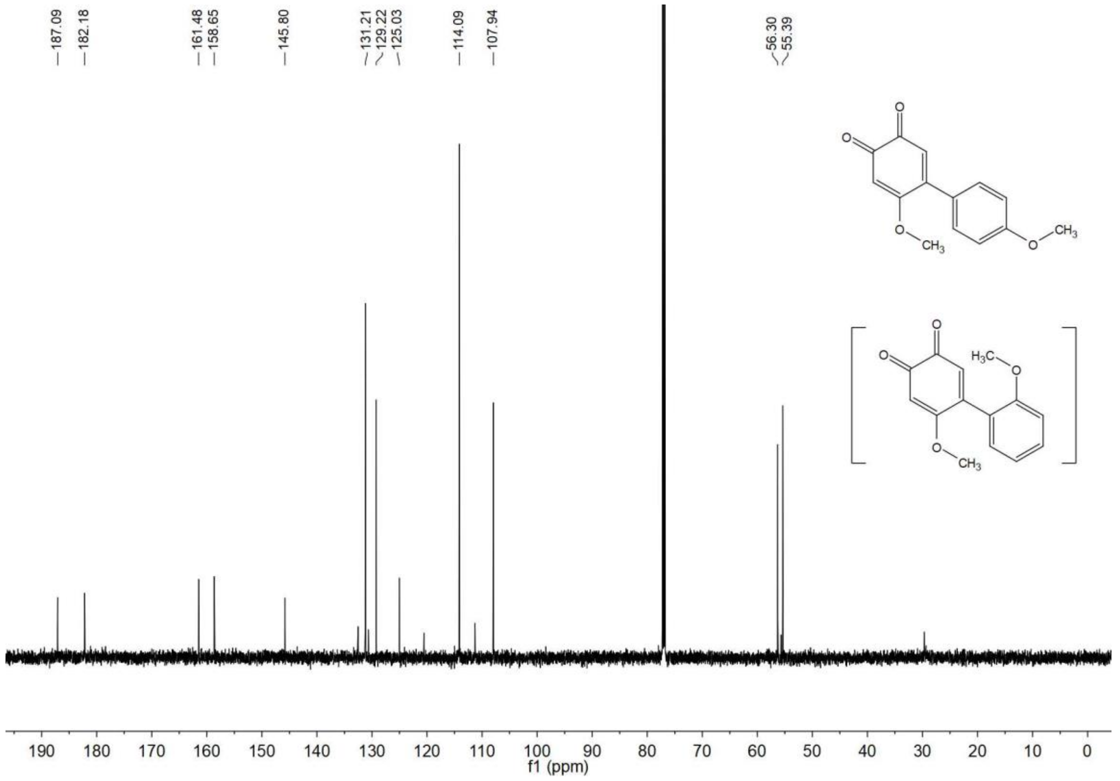

^{1}H NMR (**2e**)

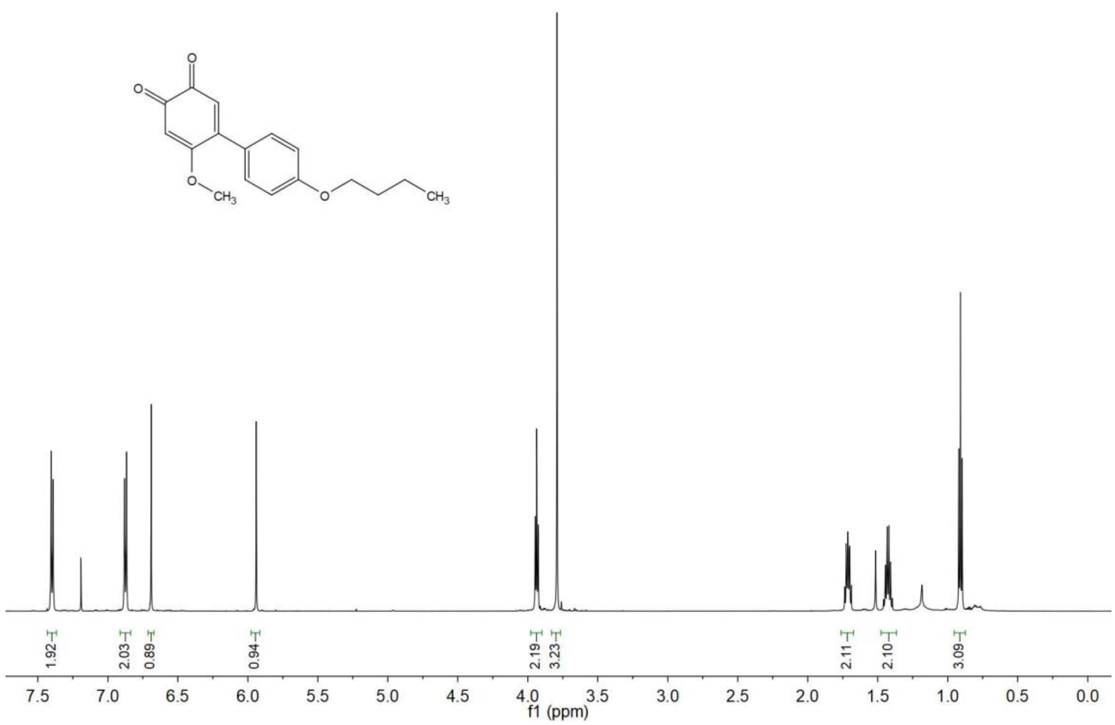

^{13}C NMR (**2e**)

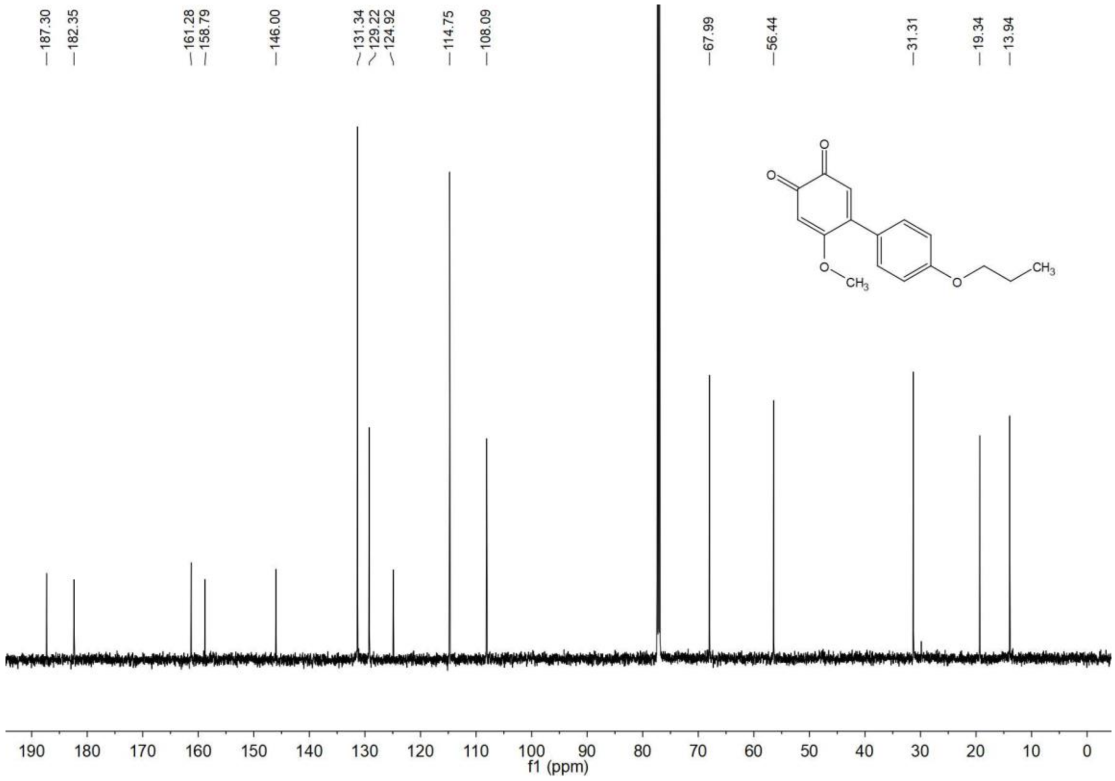

^{1}H NMR (**3e**)

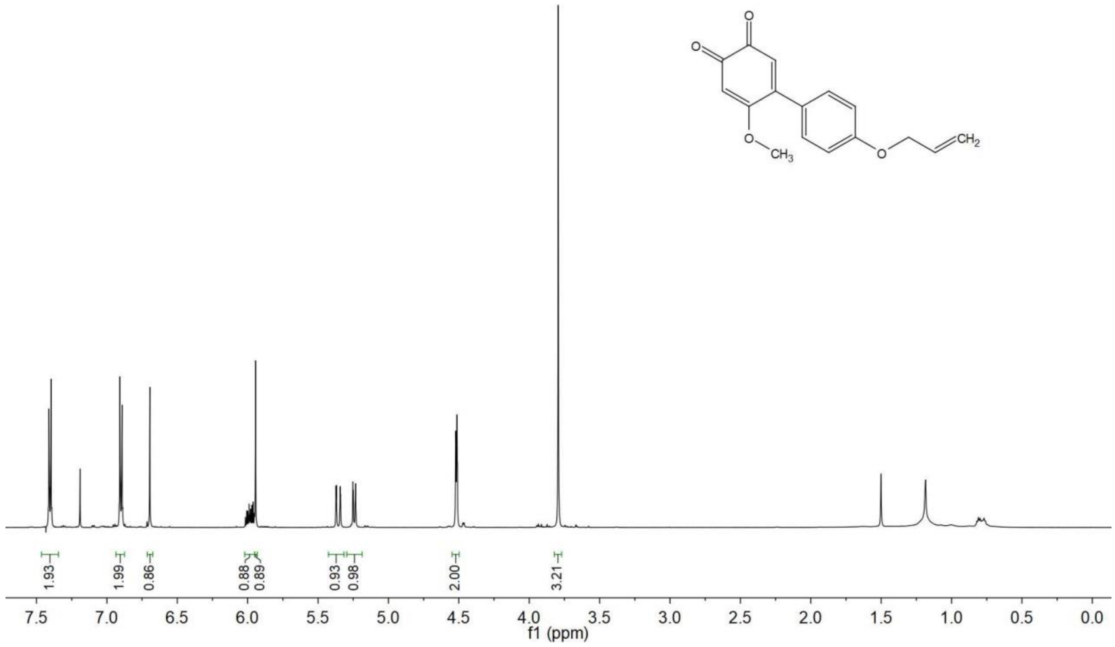

^{13}C NMR (**3e**)

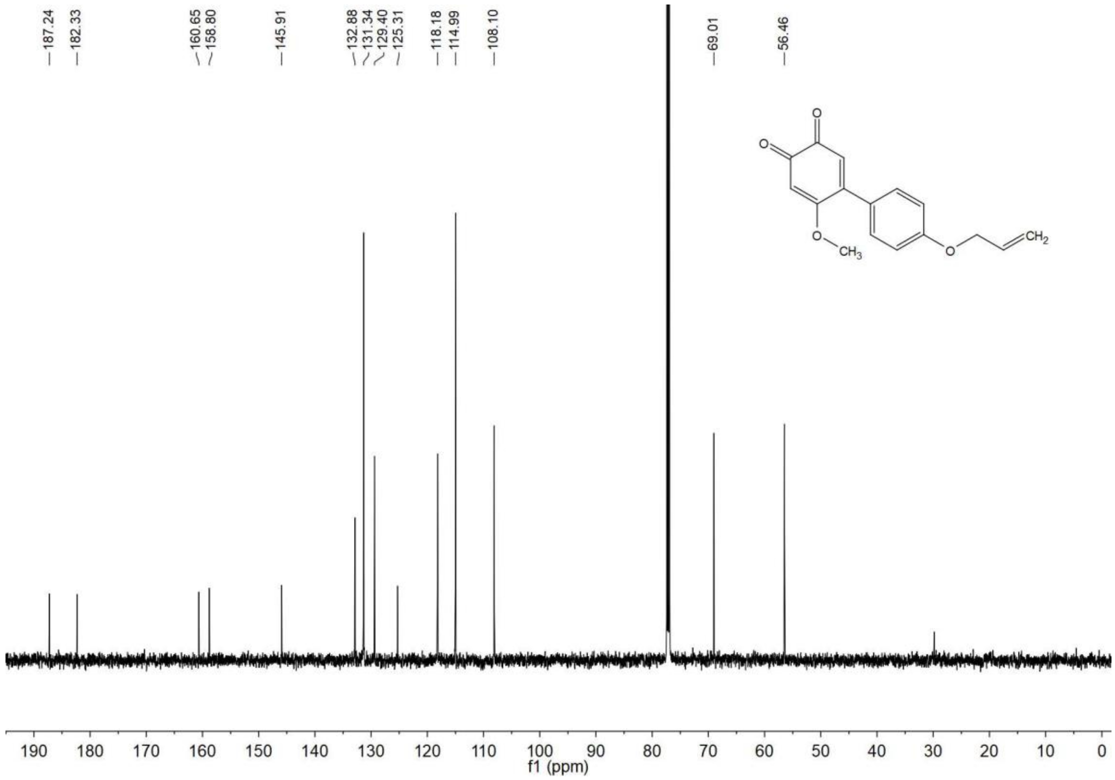

^{1}H NMR (**4e**)

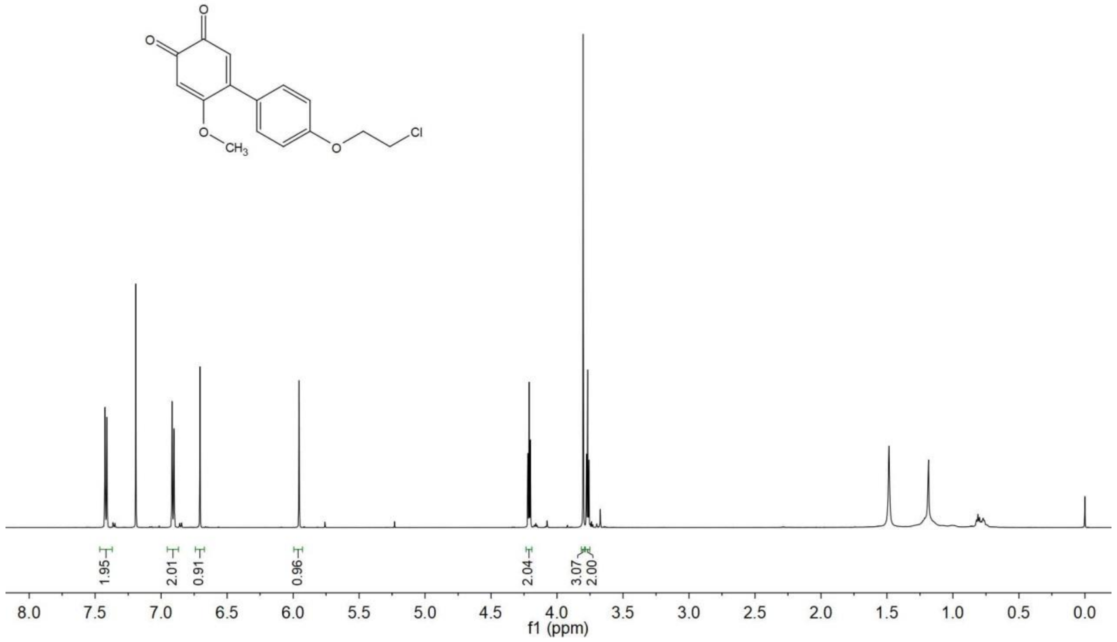

^{13}C NMR (**4e**)

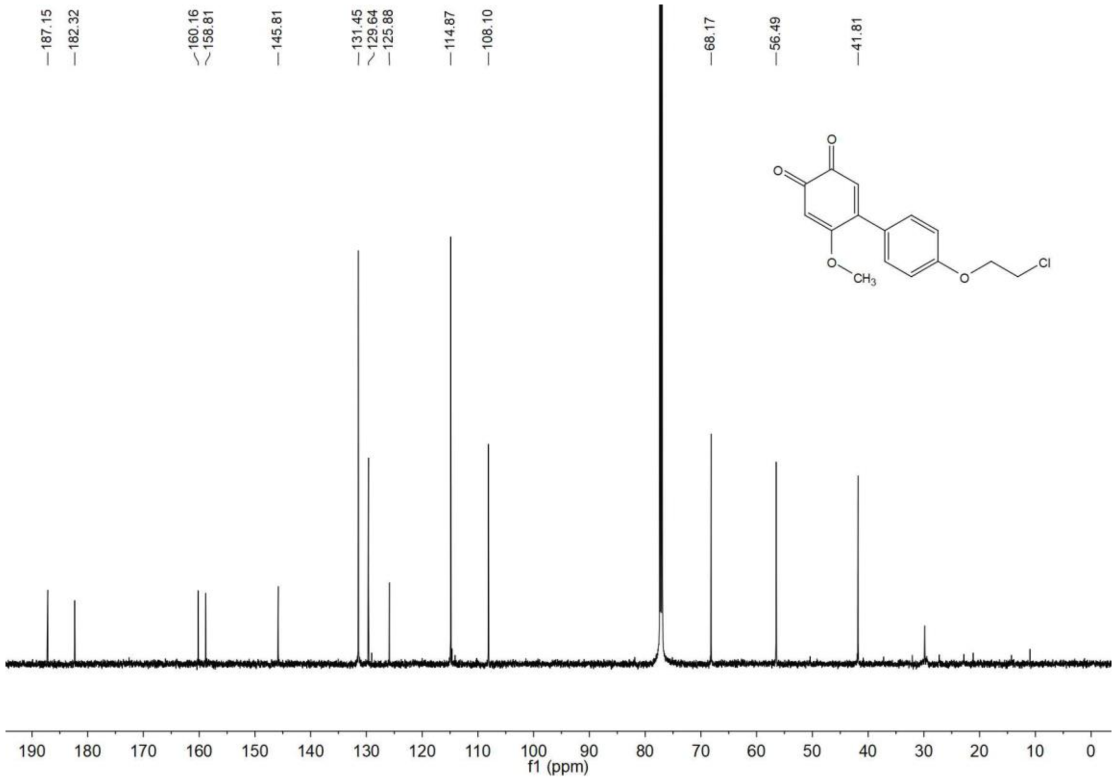

^{1}H NMR (**5e**)

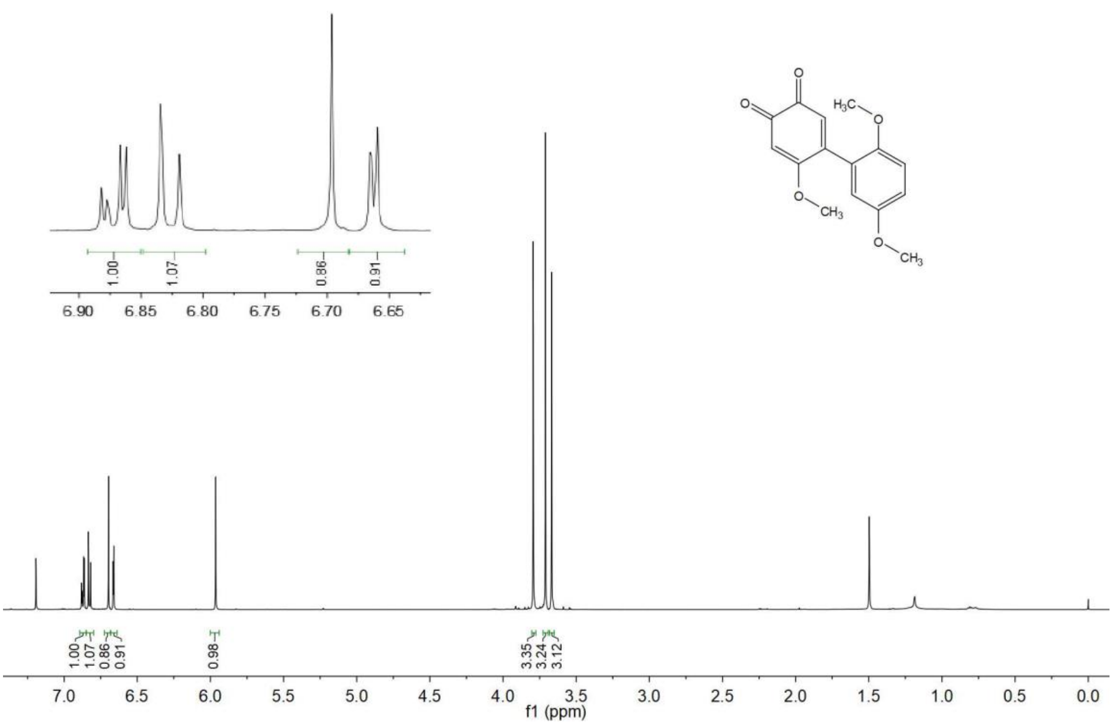

^{13}C NMR (**5e**)

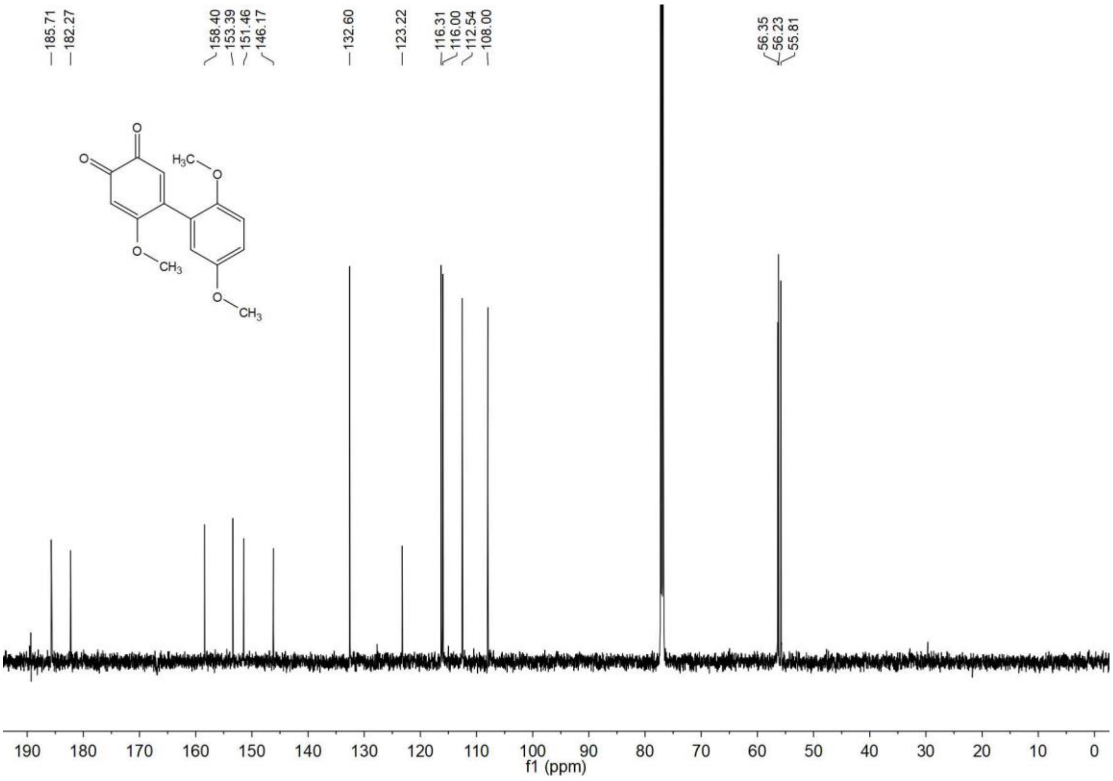

^{1}H NMR (**6e**)

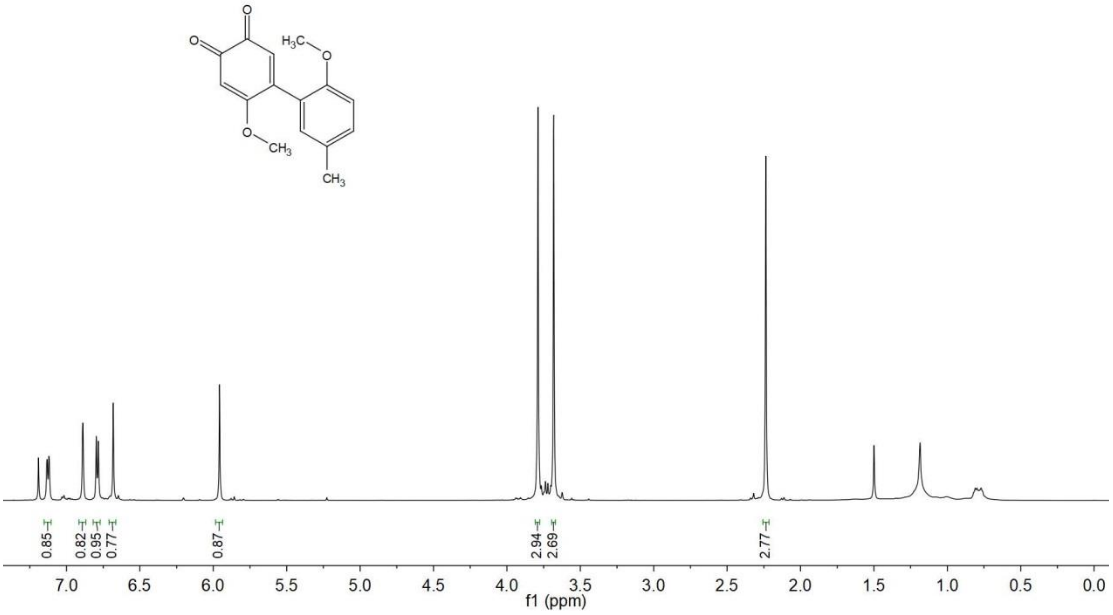

^{13}C NMR (**6e**)

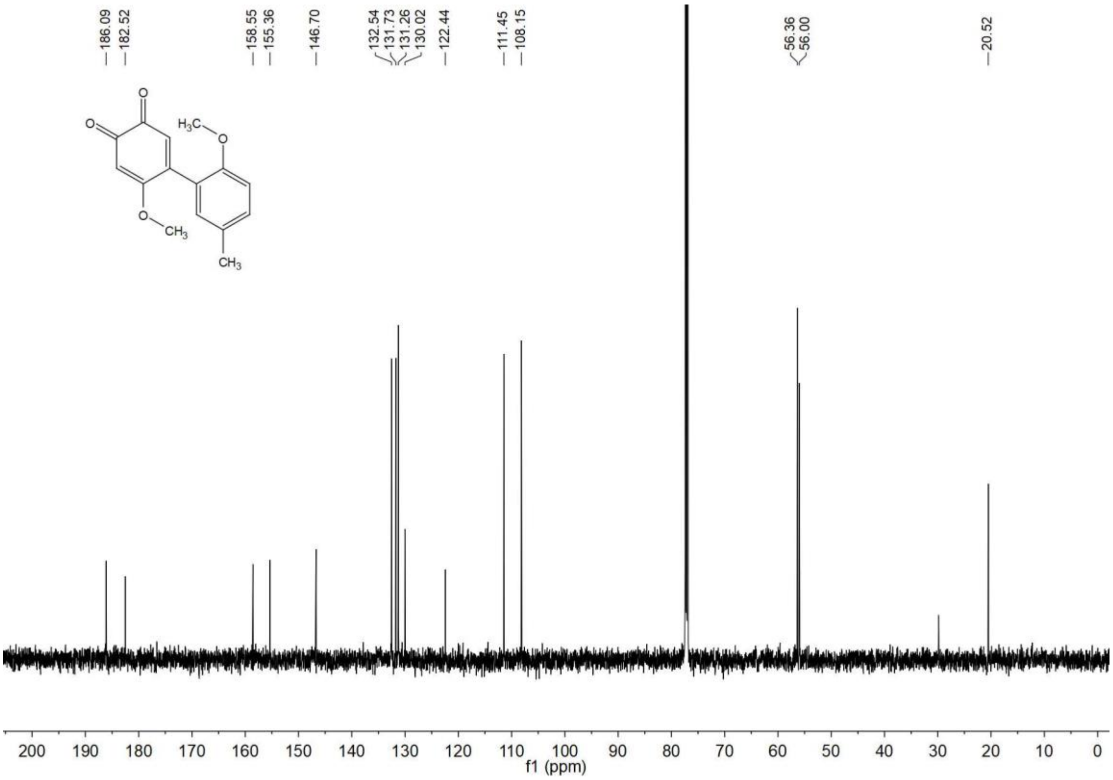

^{1}H NMR (**7e**)

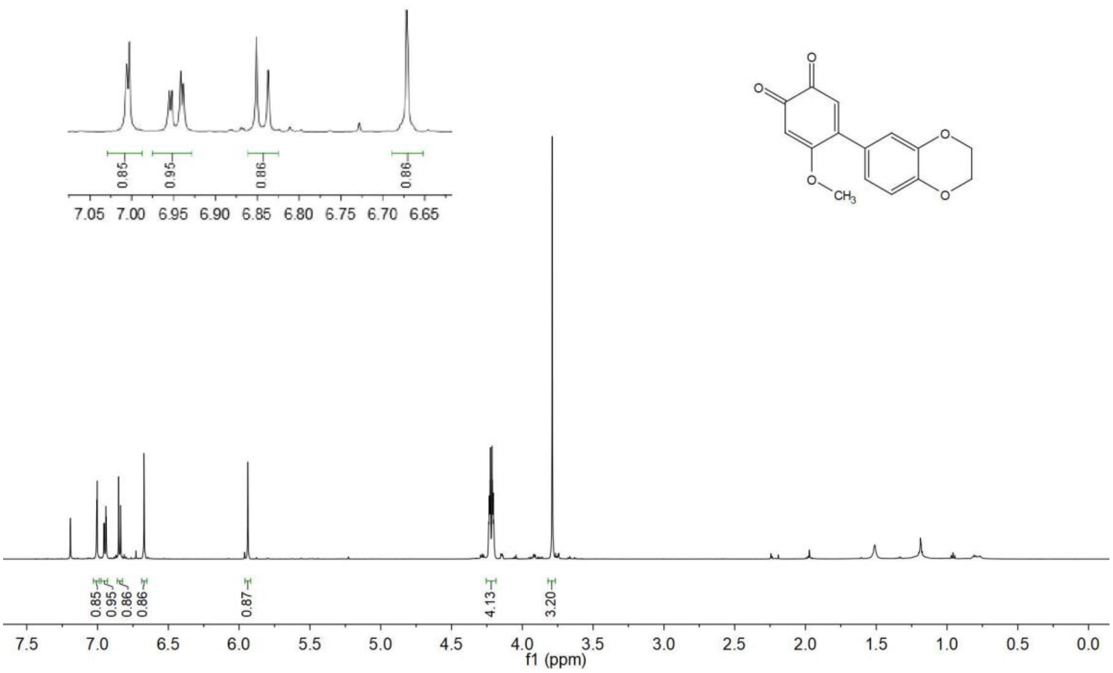

^{13}C NMR (**7e**)

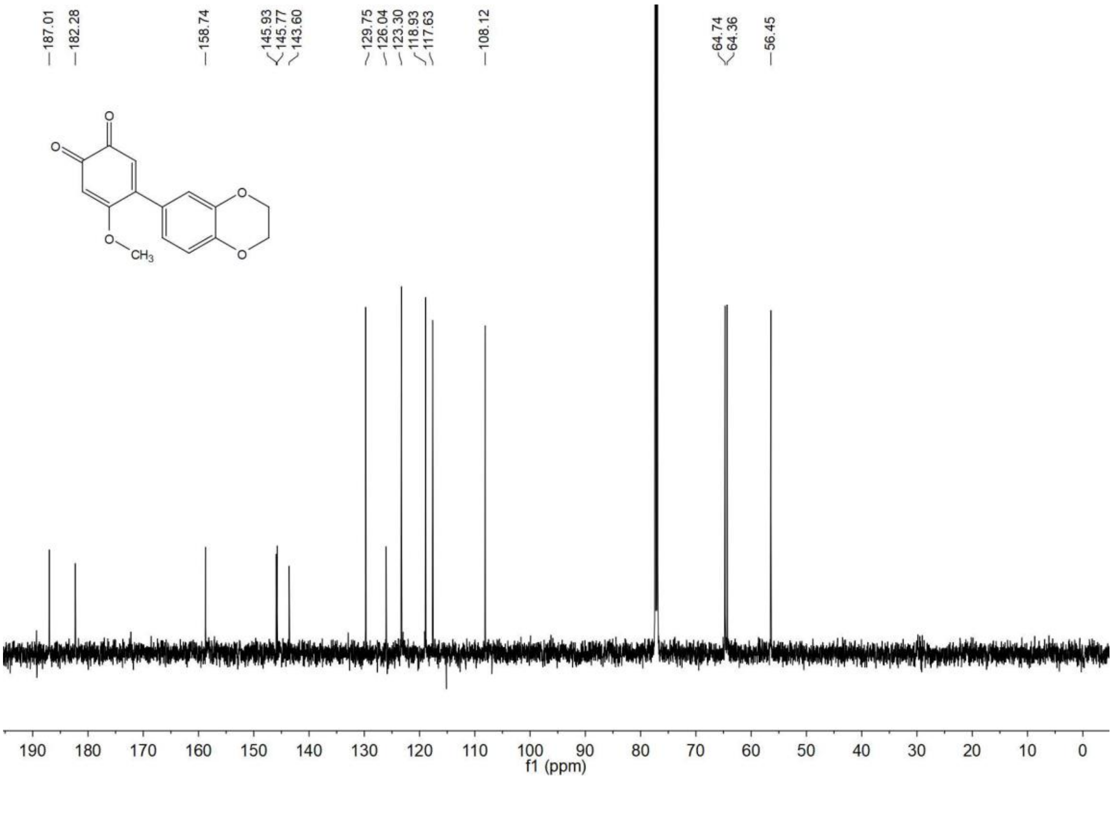

^{1}H NMR (**8e**)

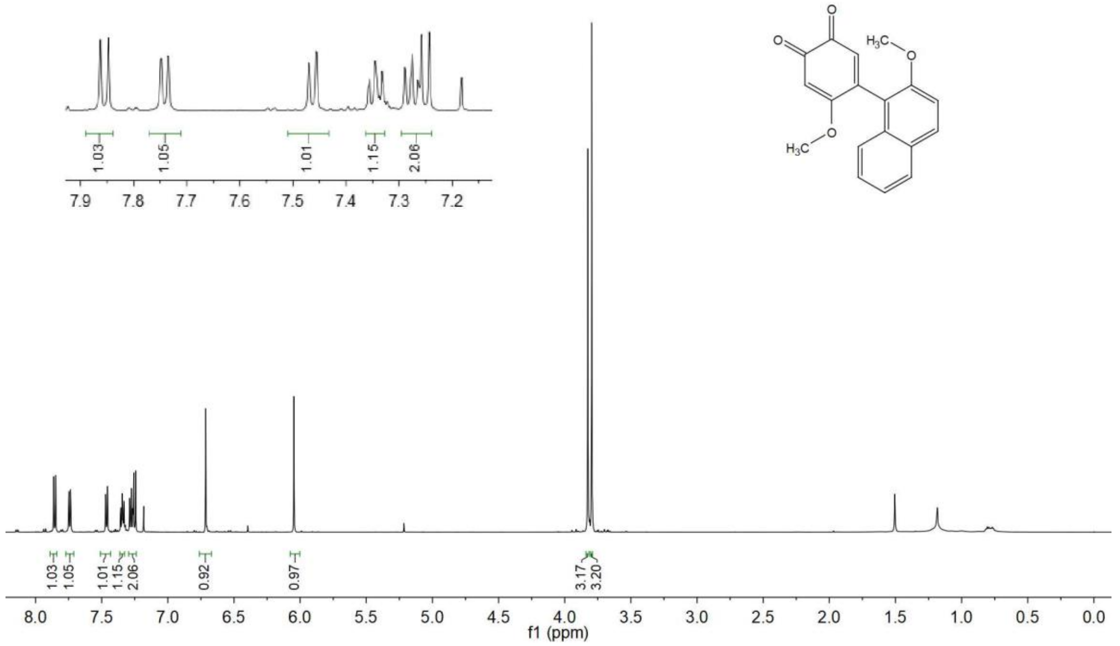

^{13}C NMR (**8e**)

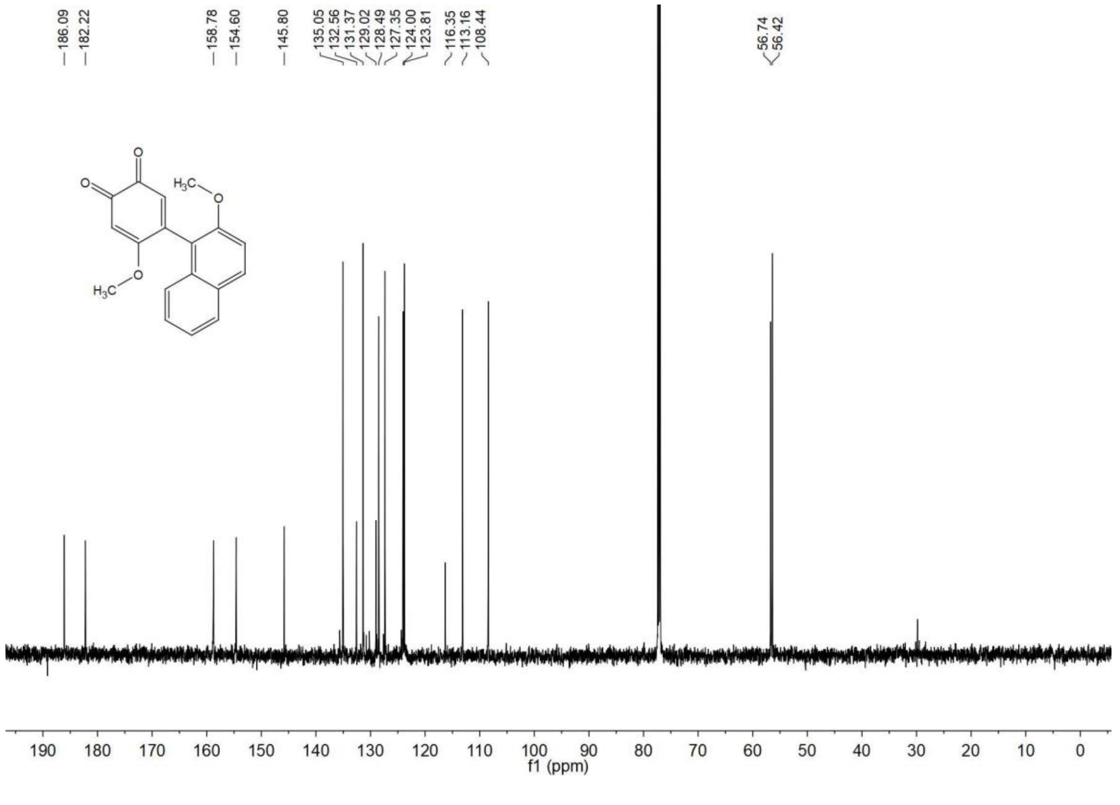

^{1}H NMR (**9e**)

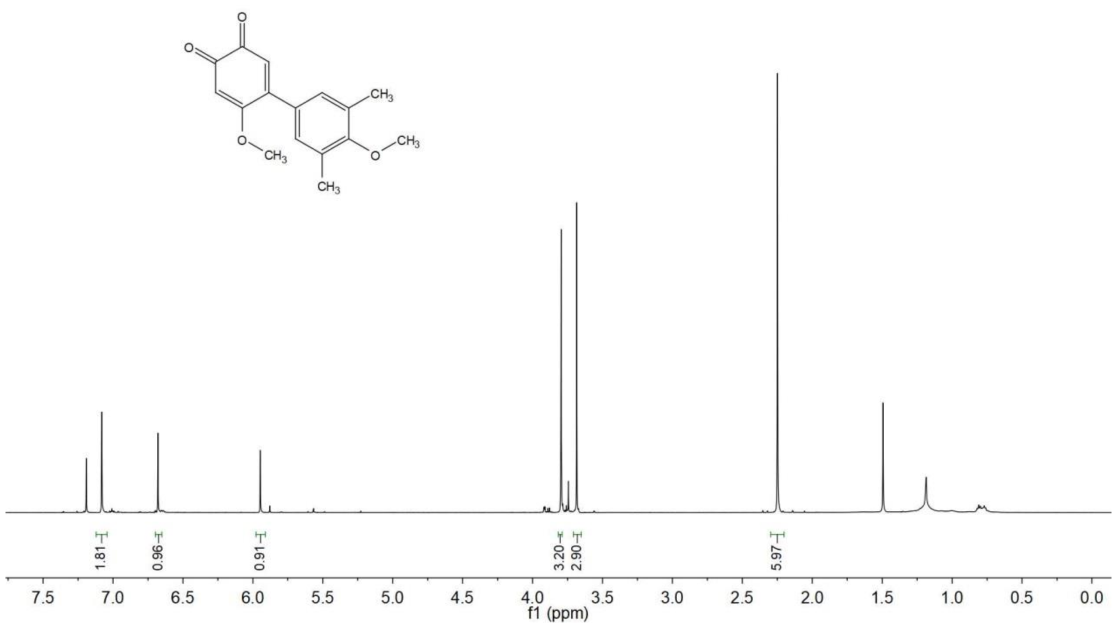

^{13}C NMR (**9e**)

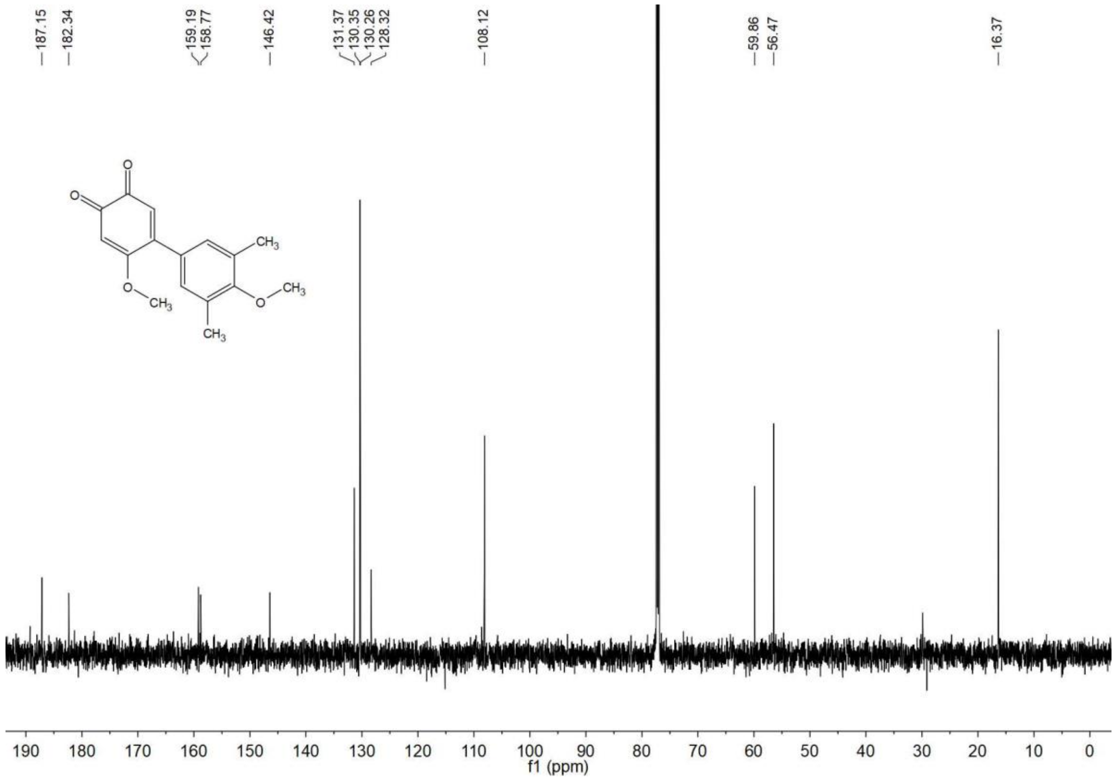

^{1}H NMR (**1g**)

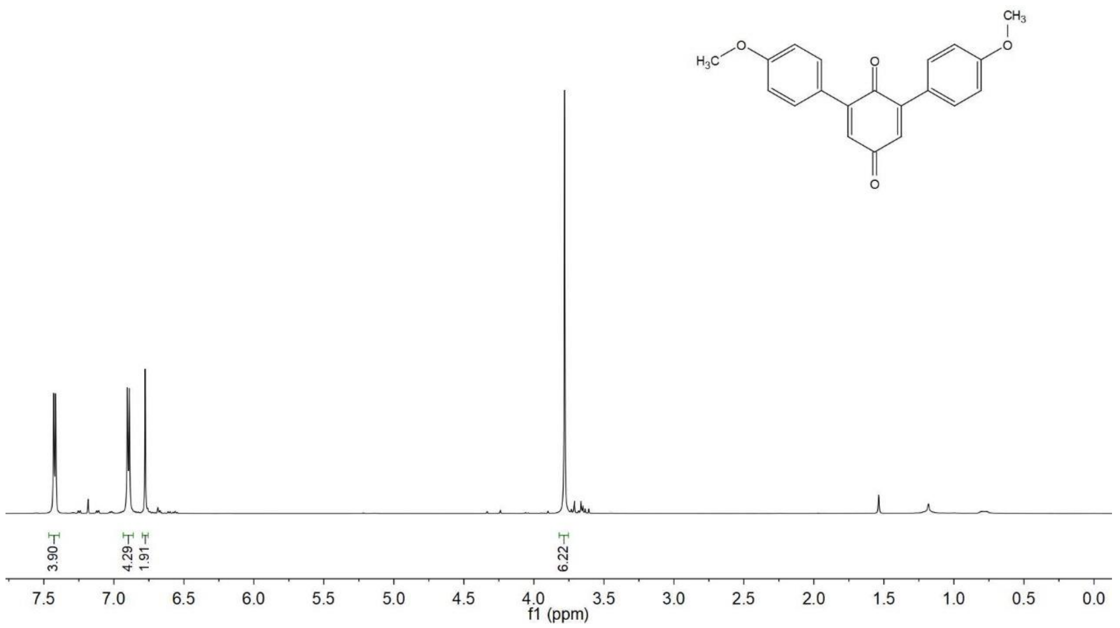

^{13}C NMR (**1g**)

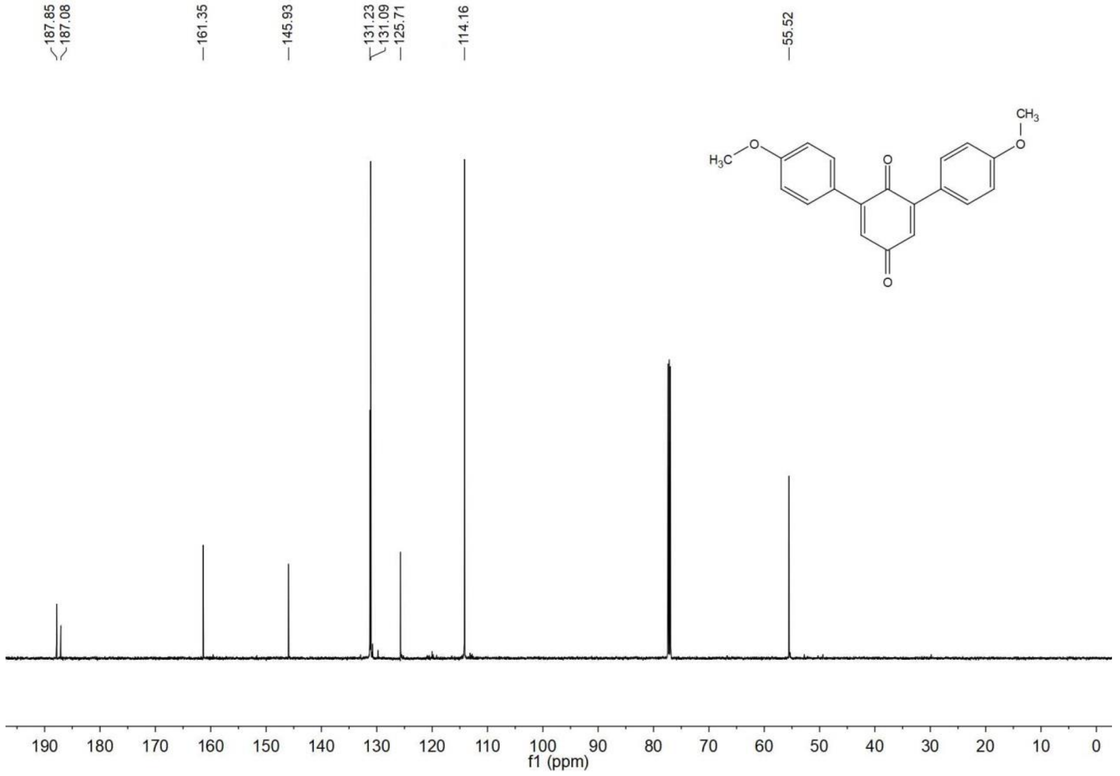

^{1}H NMR (**2g**)

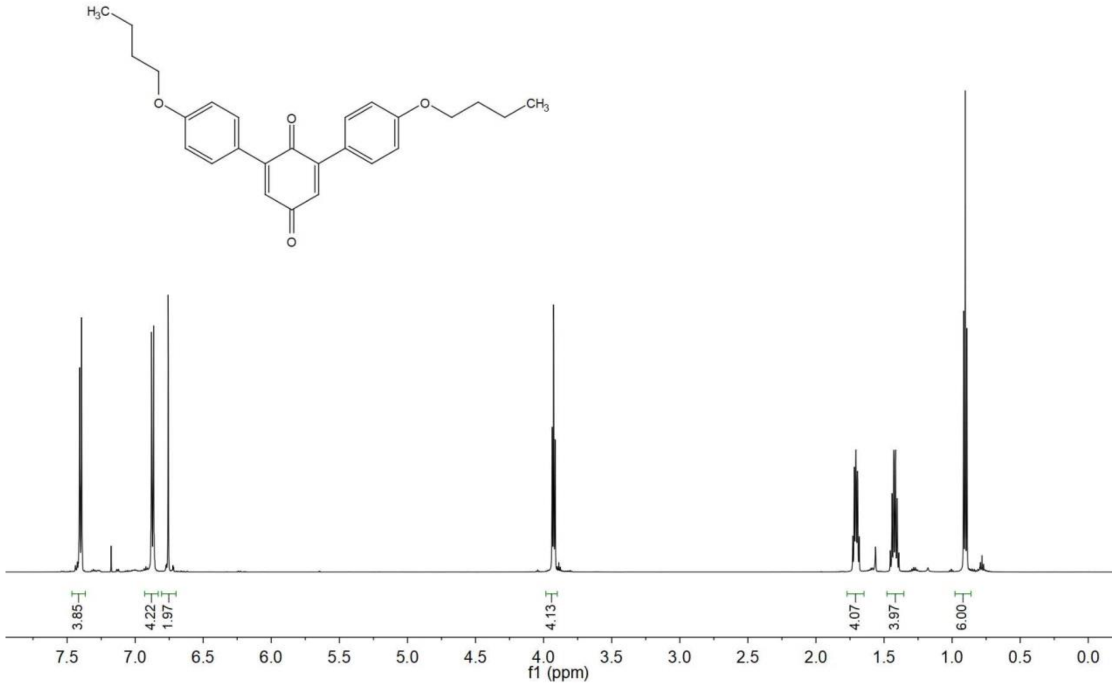

^{13}C NMR (**2g**)

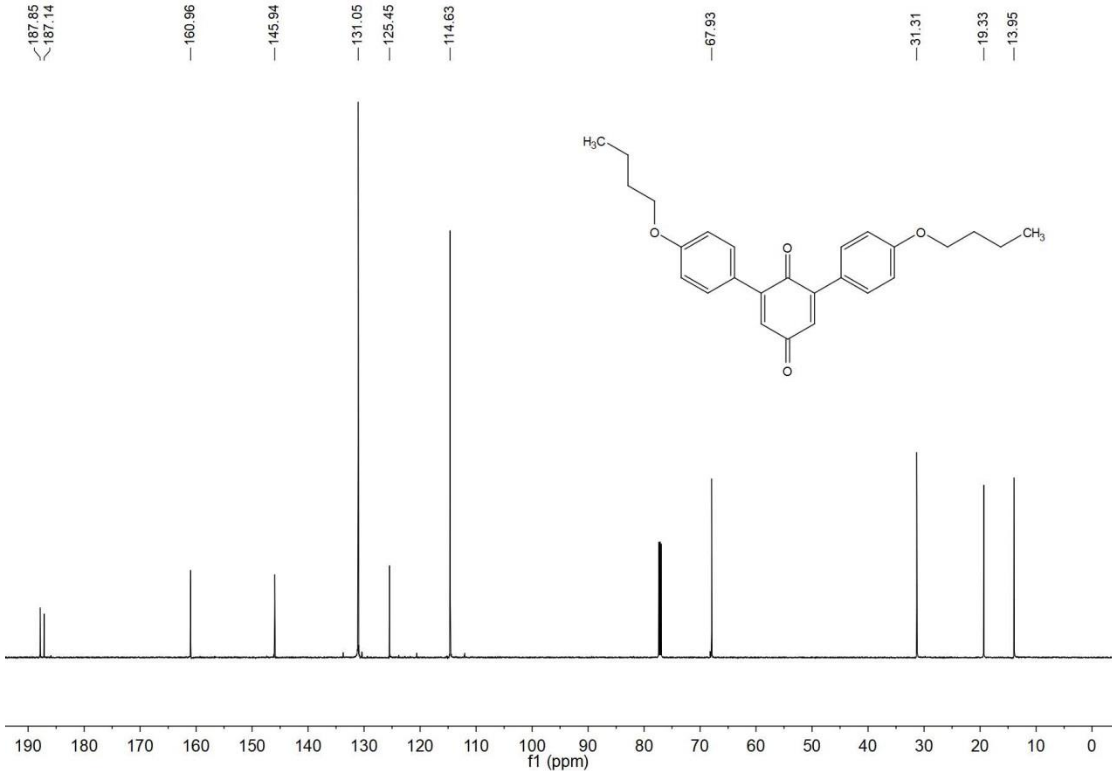

^{1}H NMR (**3g**)

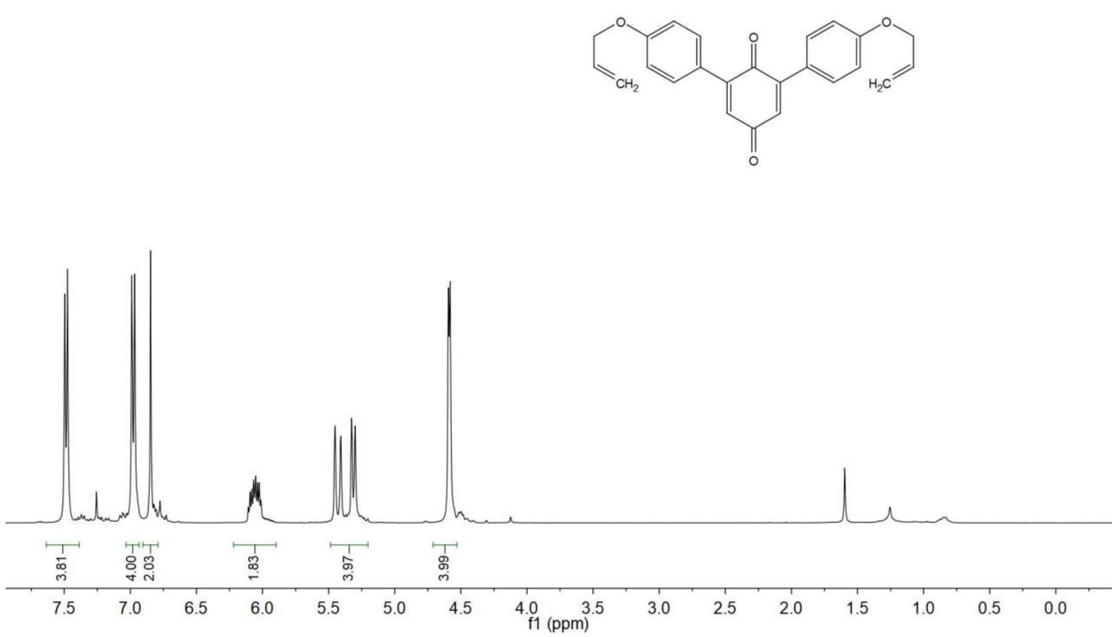

^{13}C NMR (**3g**)

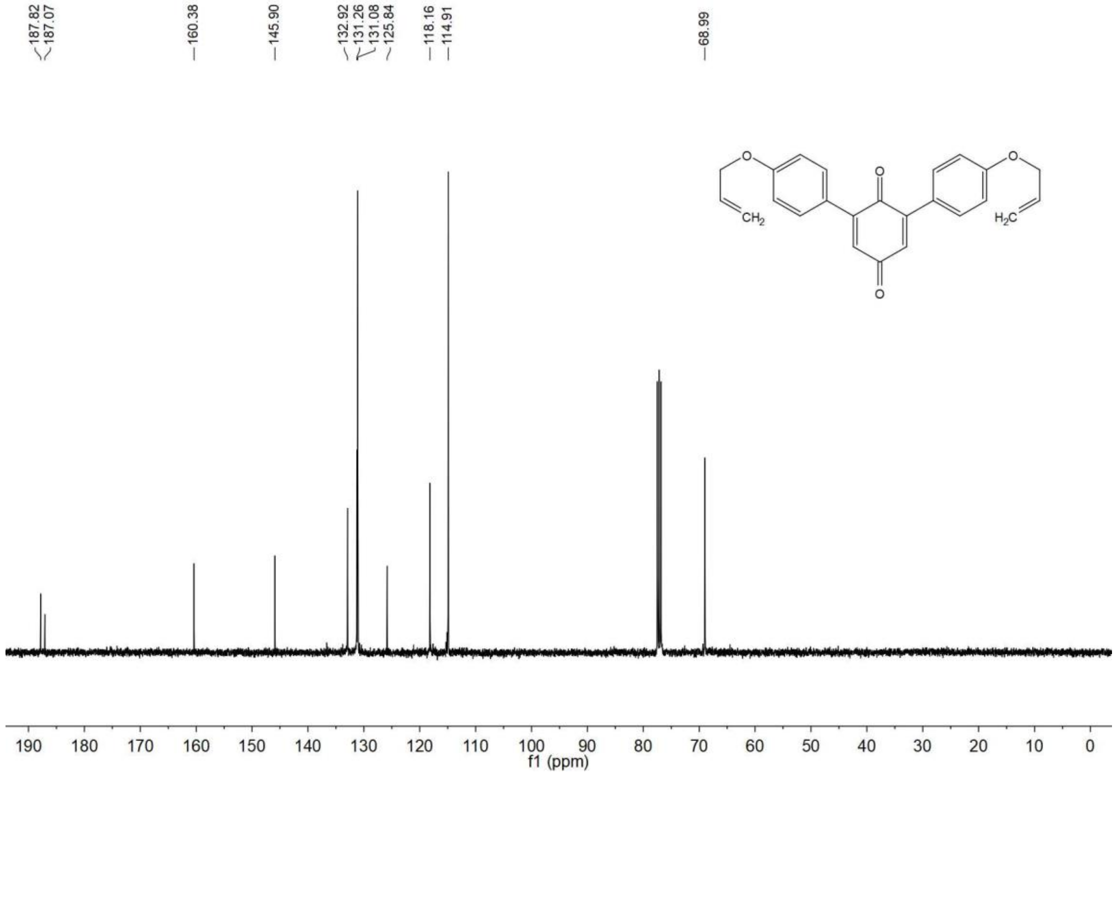

^{1}H NMR (**4g**)

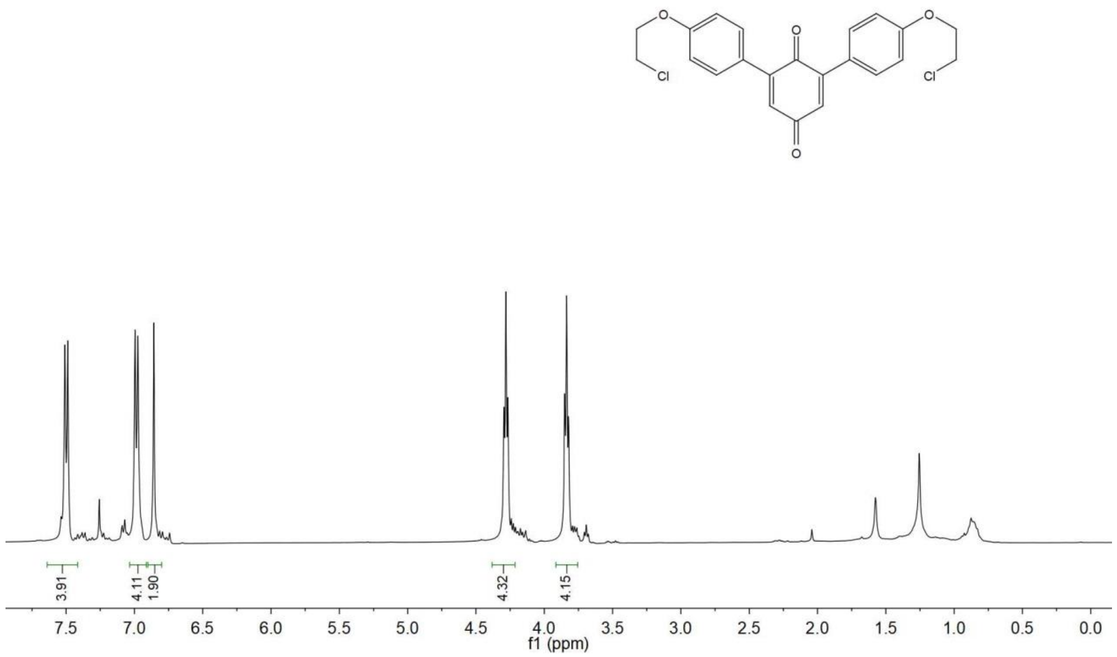

^{13}C NMR (**4g**)

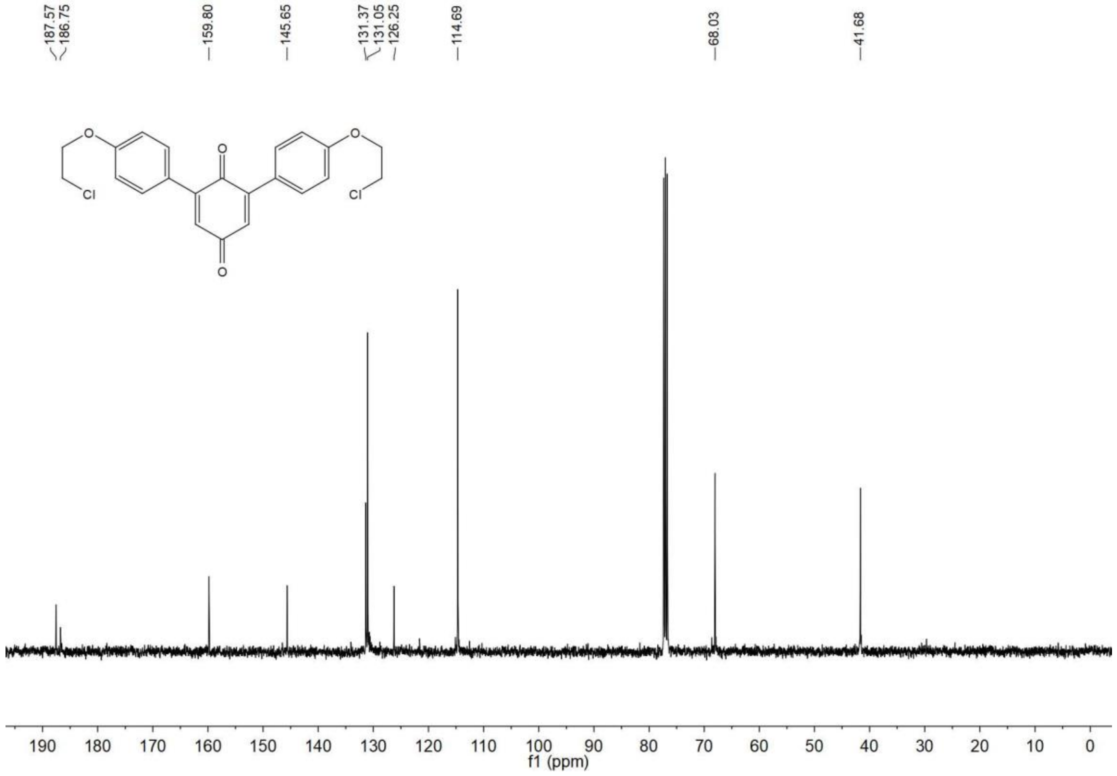

^{1}H NMR (**5g**)

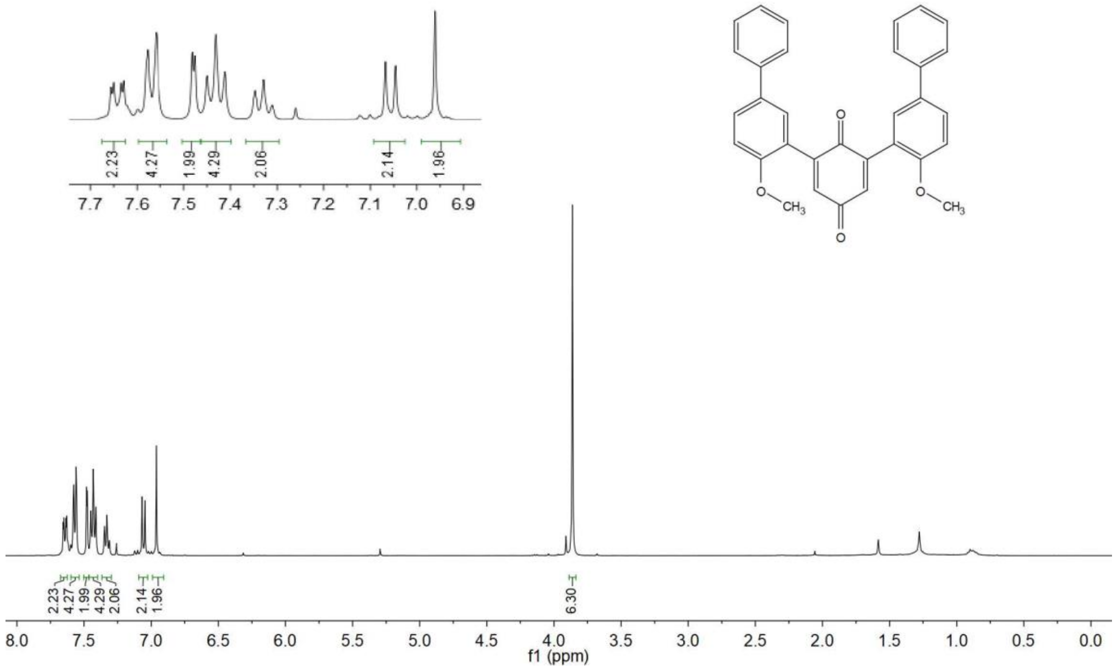

^{13}C NMR (**5g**)

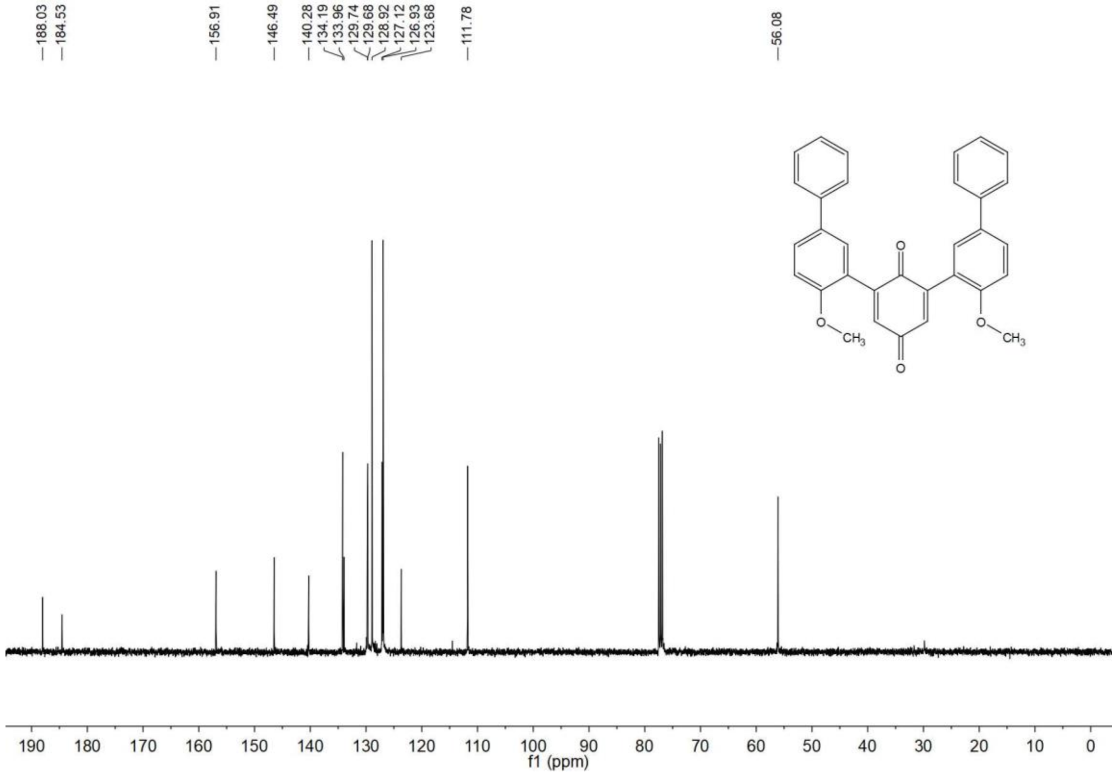

^{1}H NMR (**6g**)

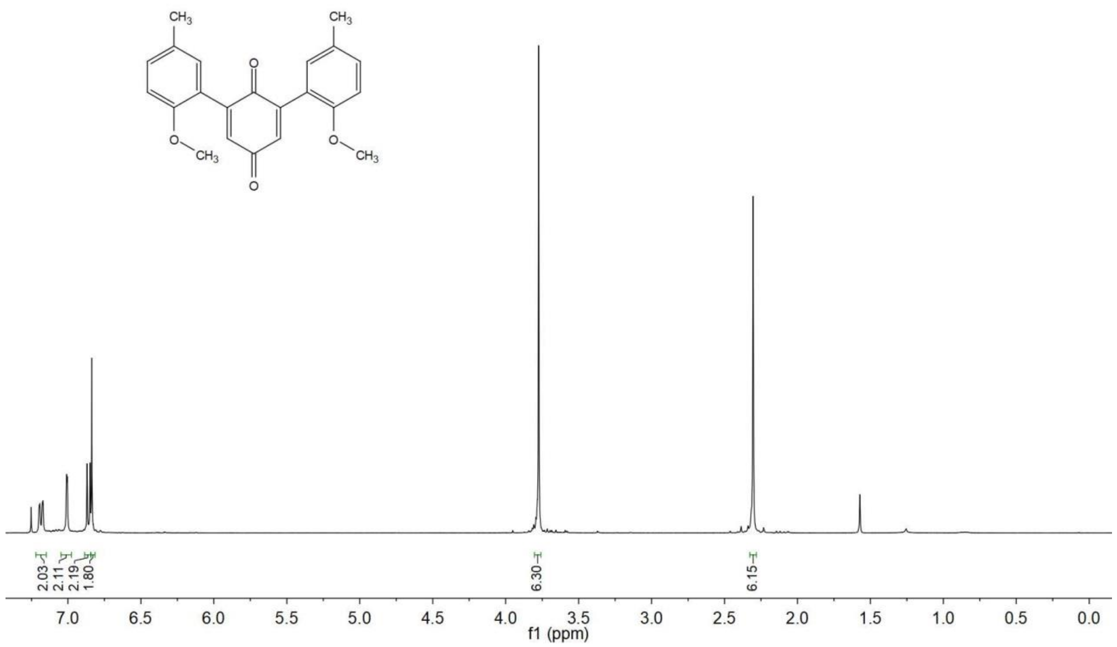

^{13}C NMR (**6g**)

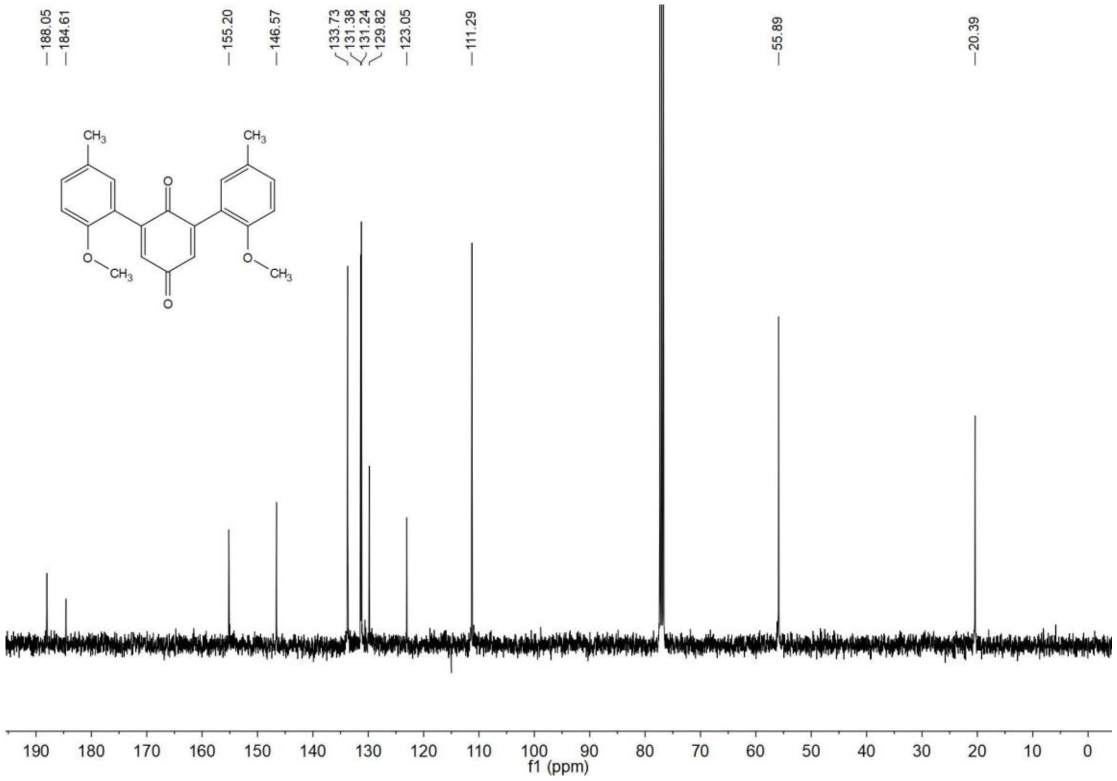

^{1}H NMR (**7g**)

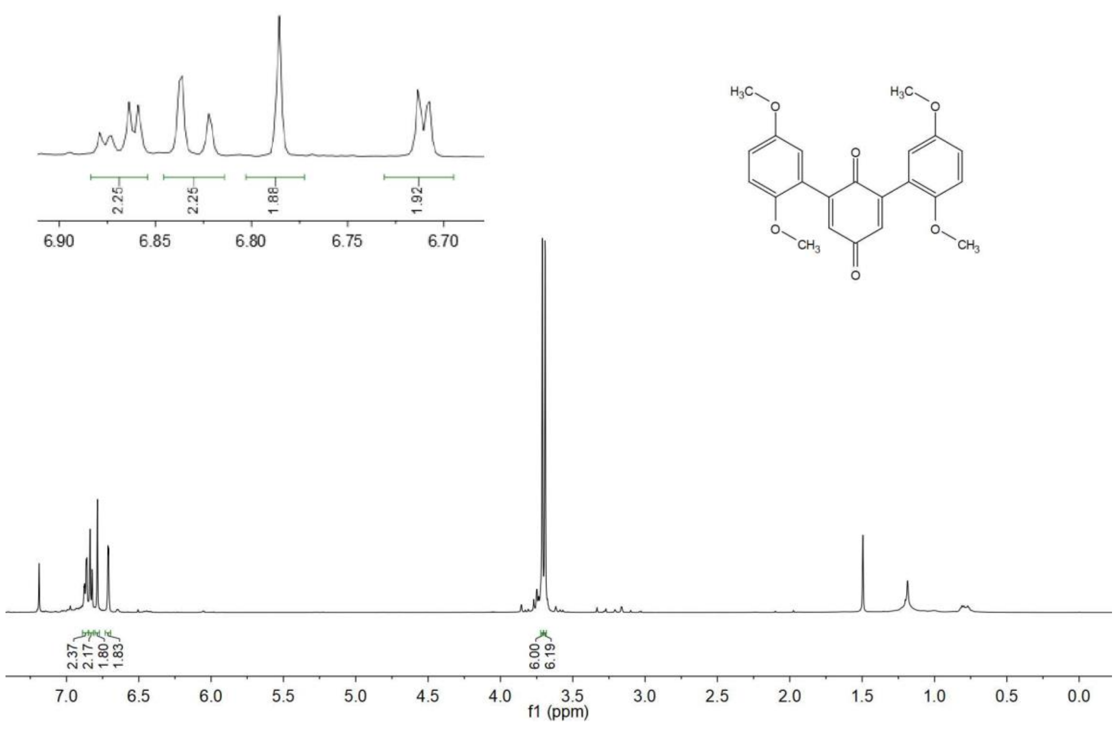

^{13}C NMR (**7g**)

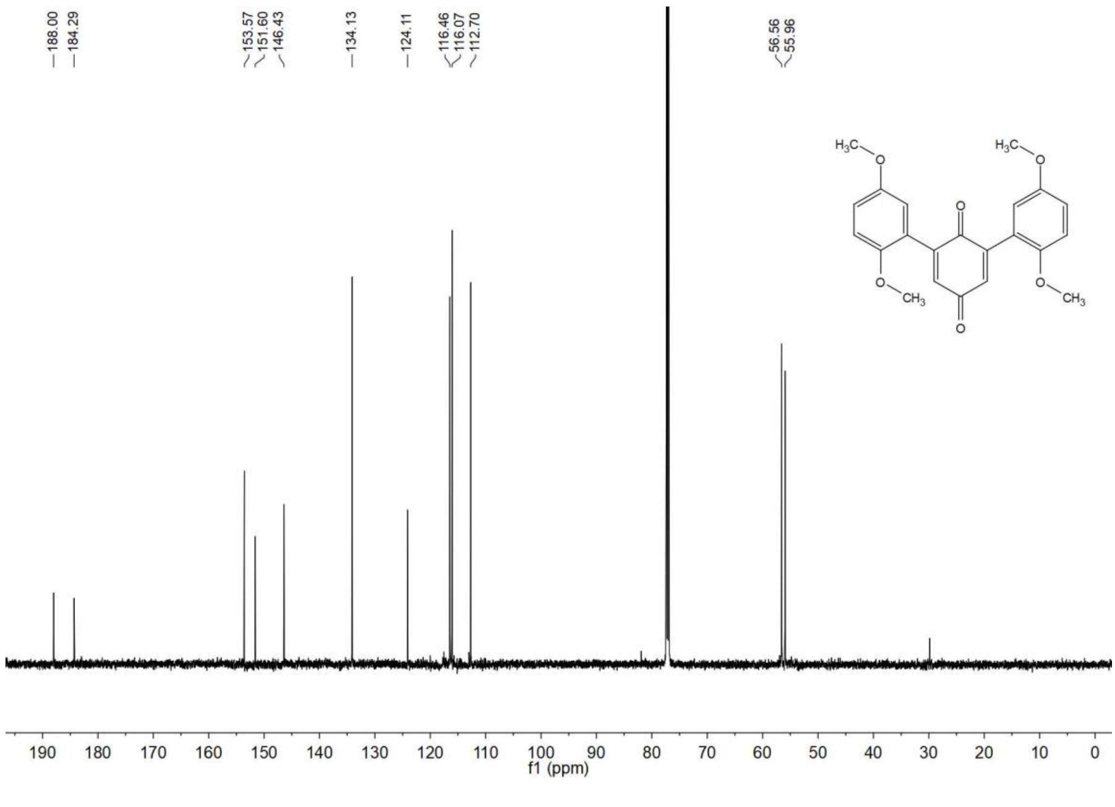

^{1}H NMR (**8g**)

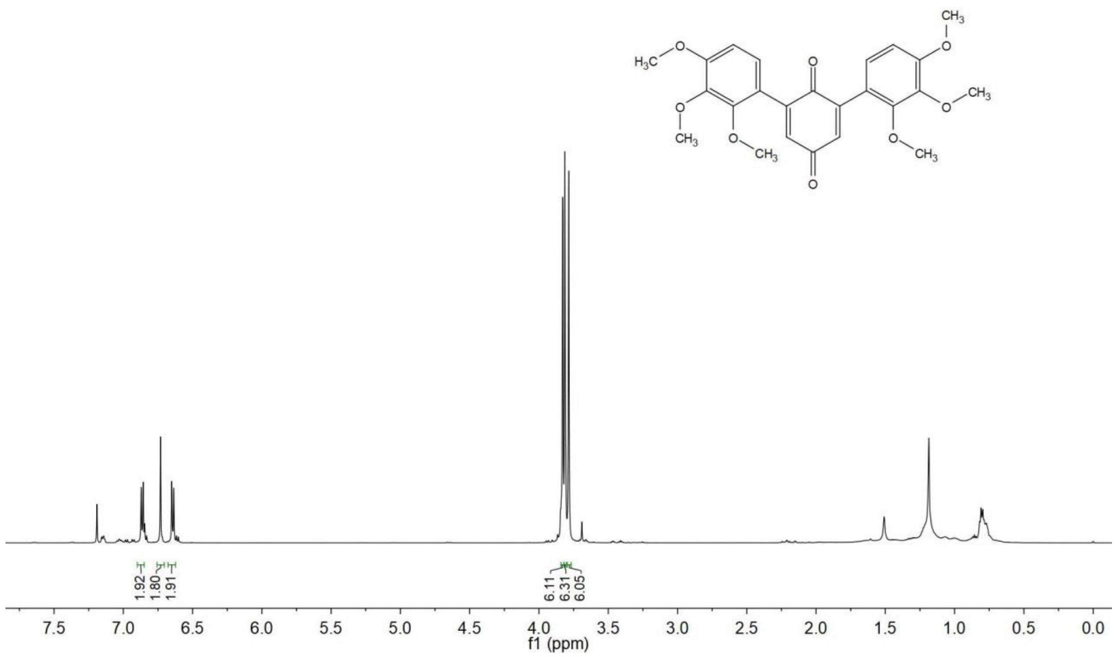

^{13}C NMR (**8g**)

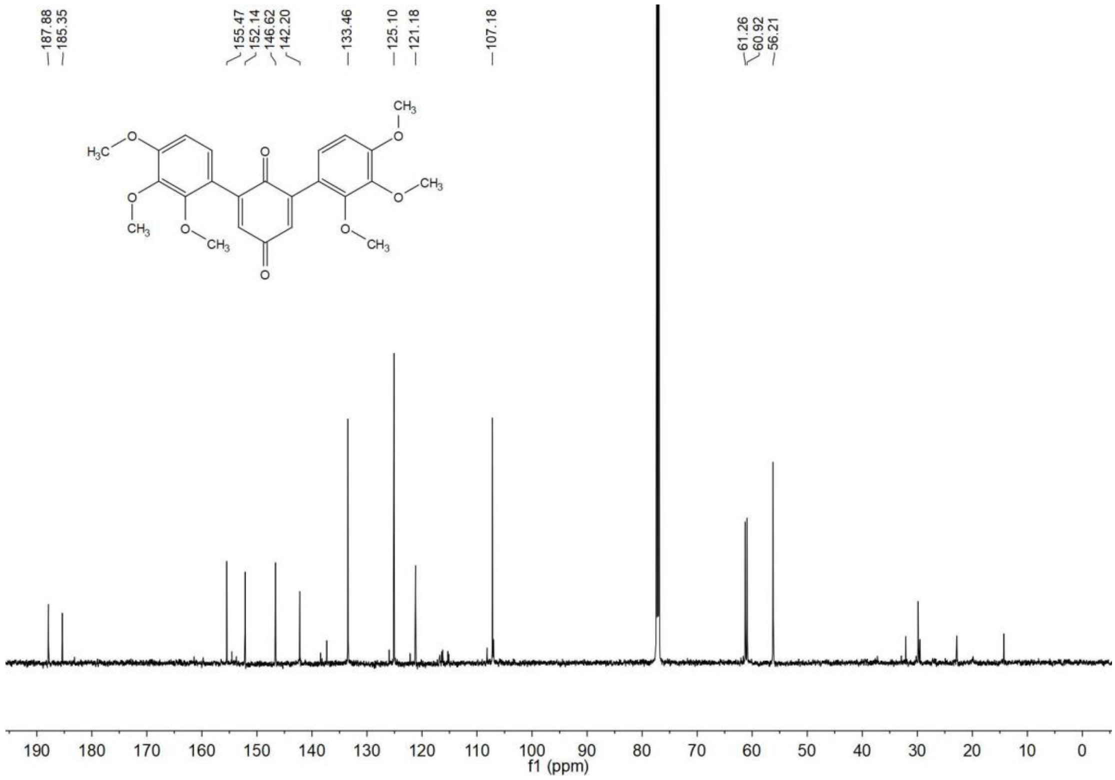

^{1}H NMR (**9g**)

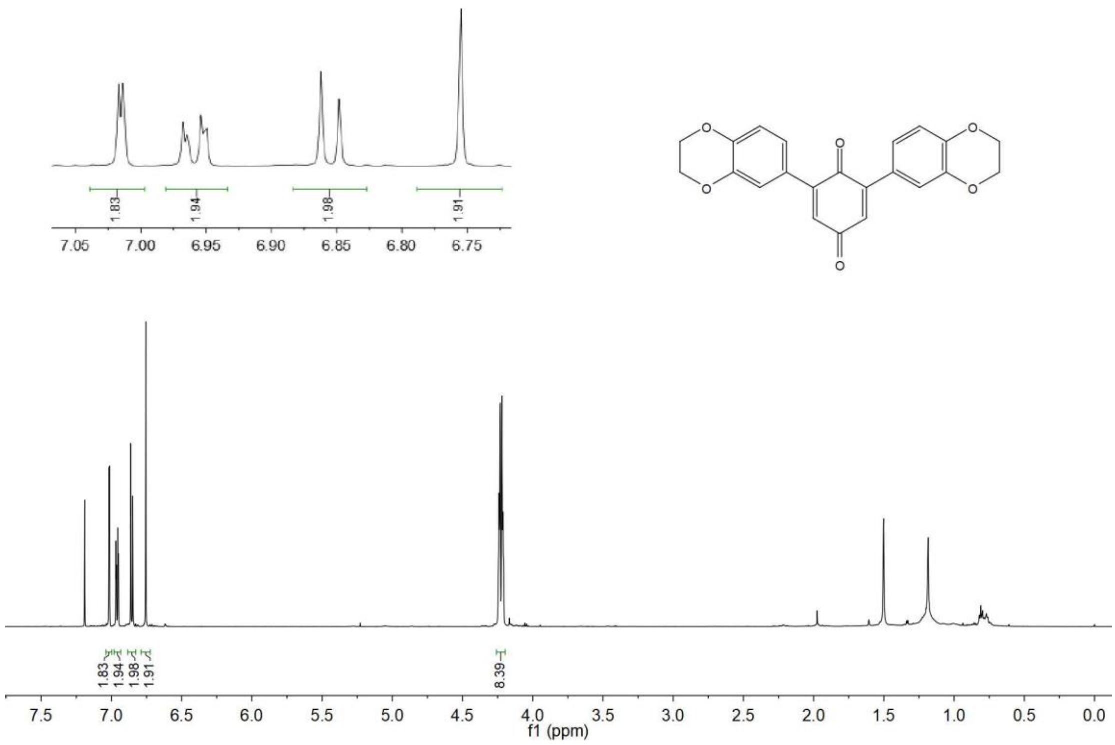

^{13}C NMR (**9g**)

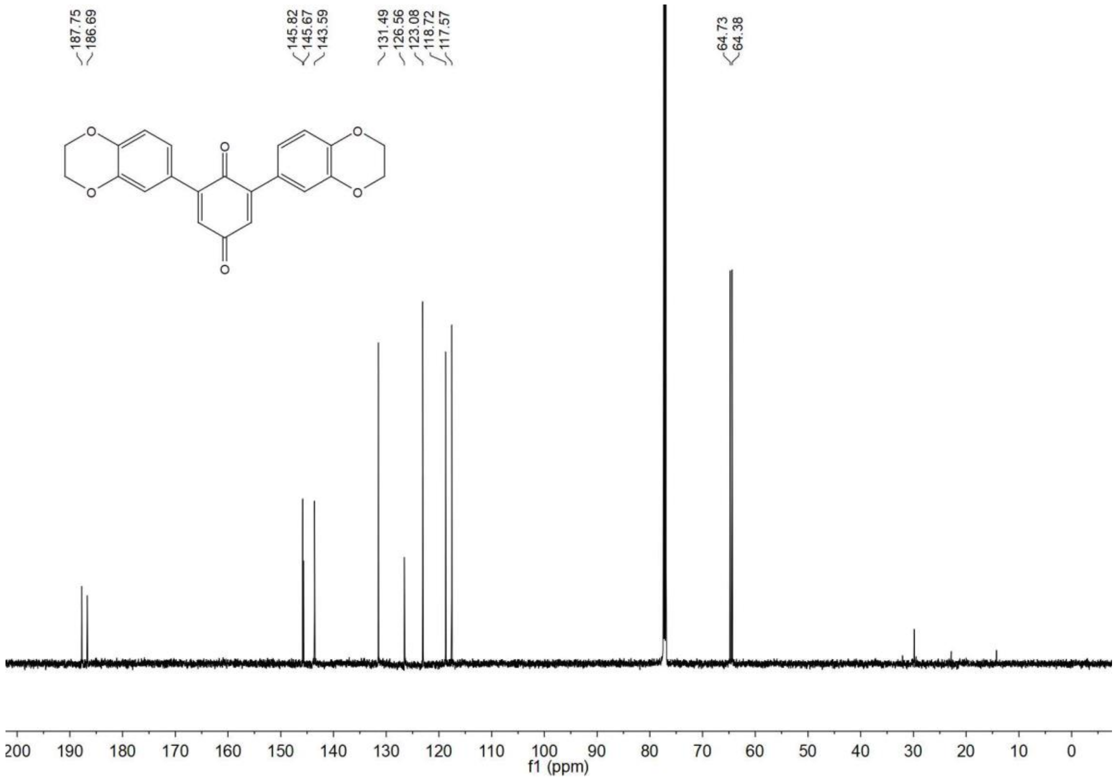

^{1}H NMR (**10g**)

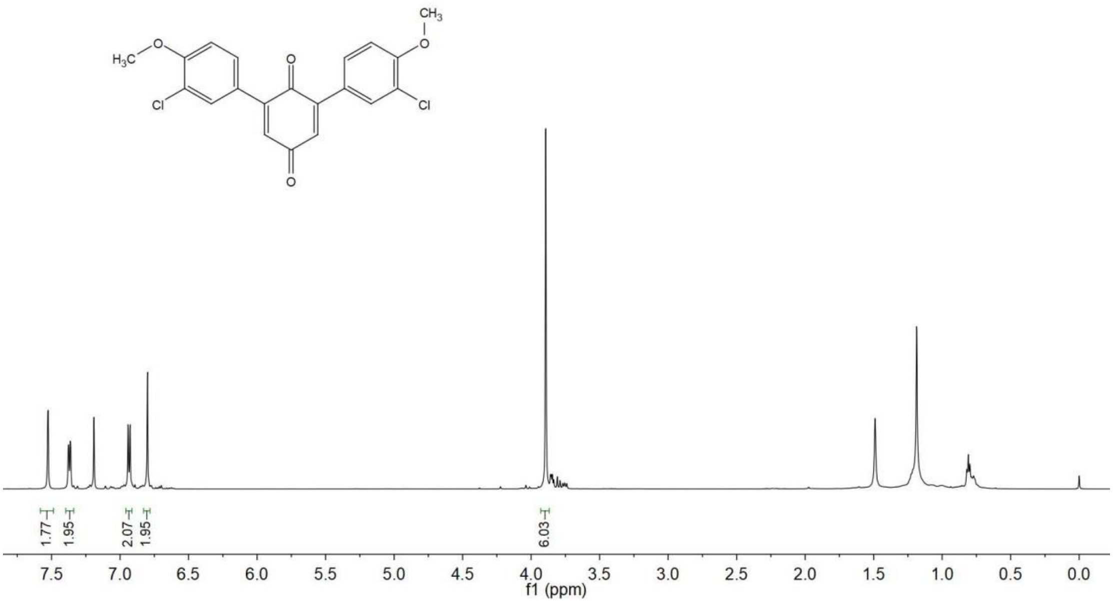

^{13}C NMR (**10g**)

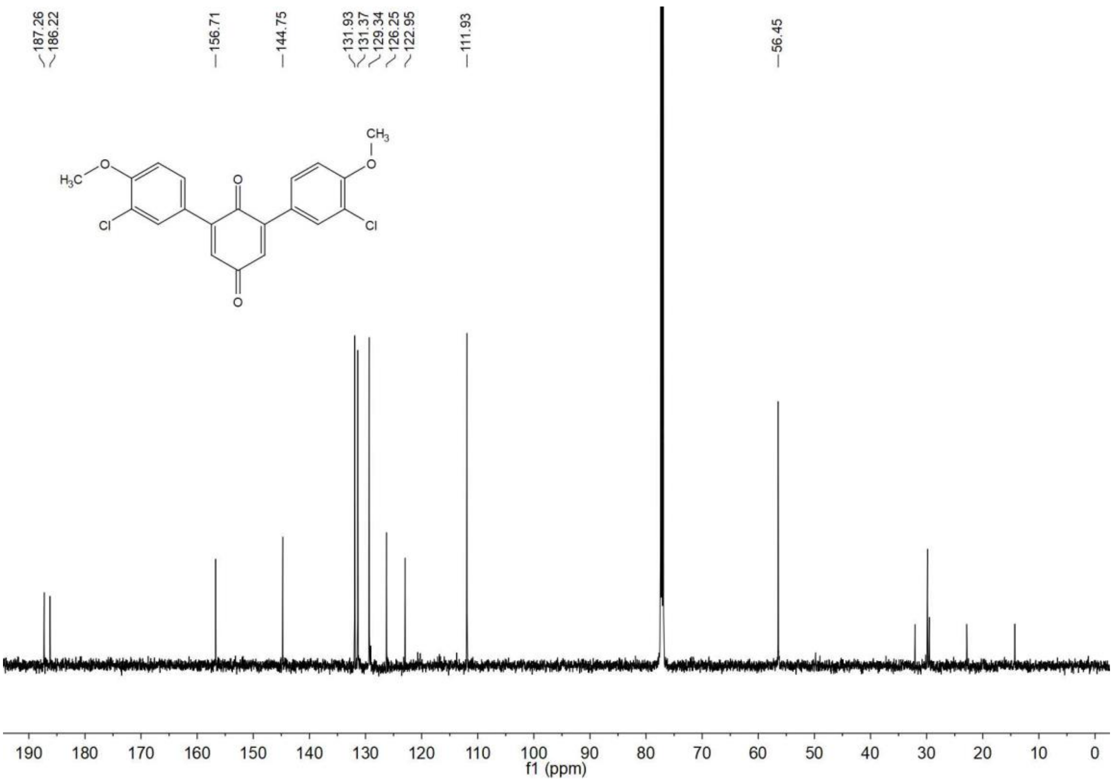

^{1}H NMR (**22c**)

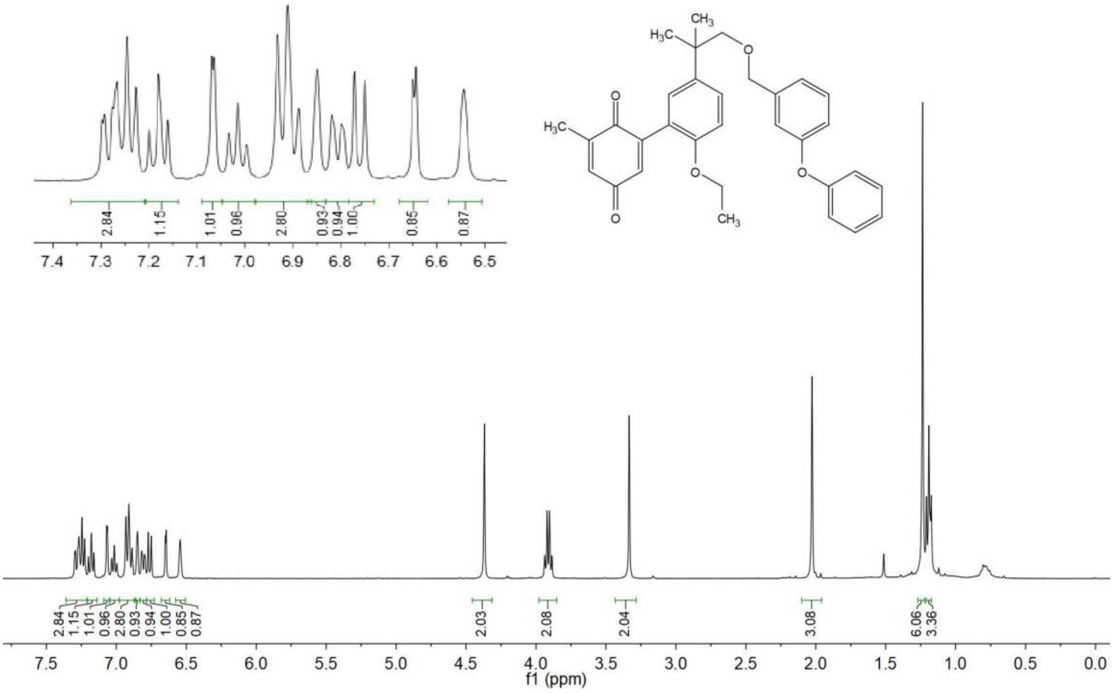

^{13}C NMR (**22c**)

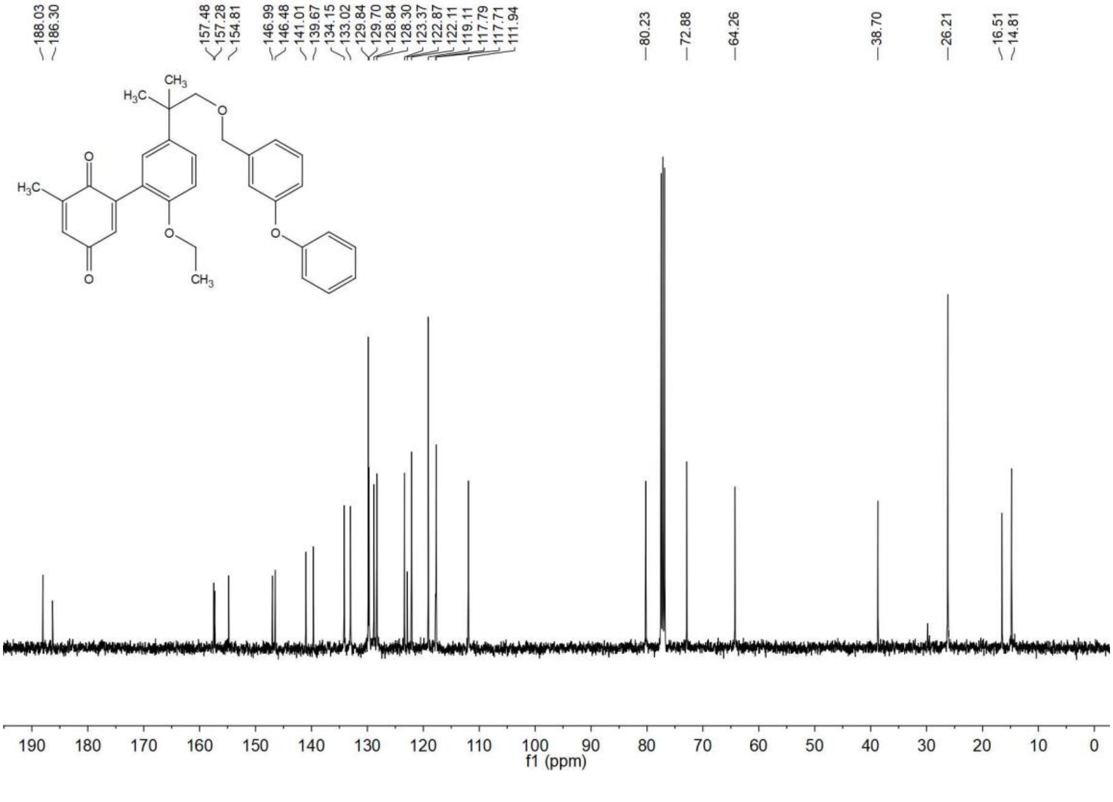

^{1}H NMR (**j**)

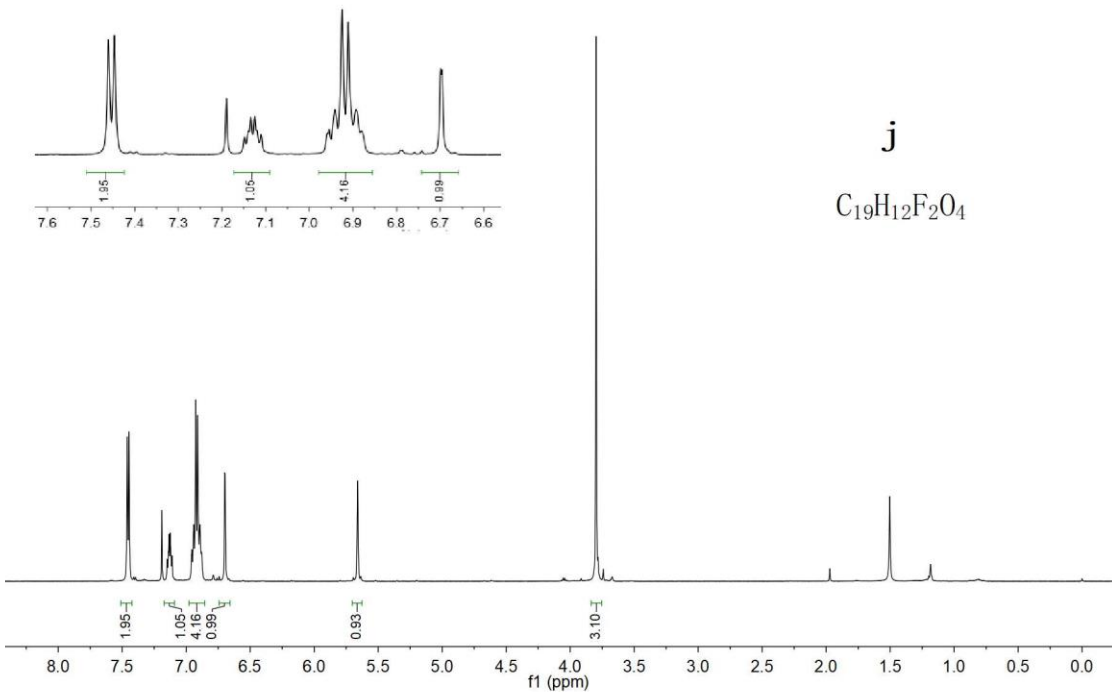

^{13}C NMR (**j**)

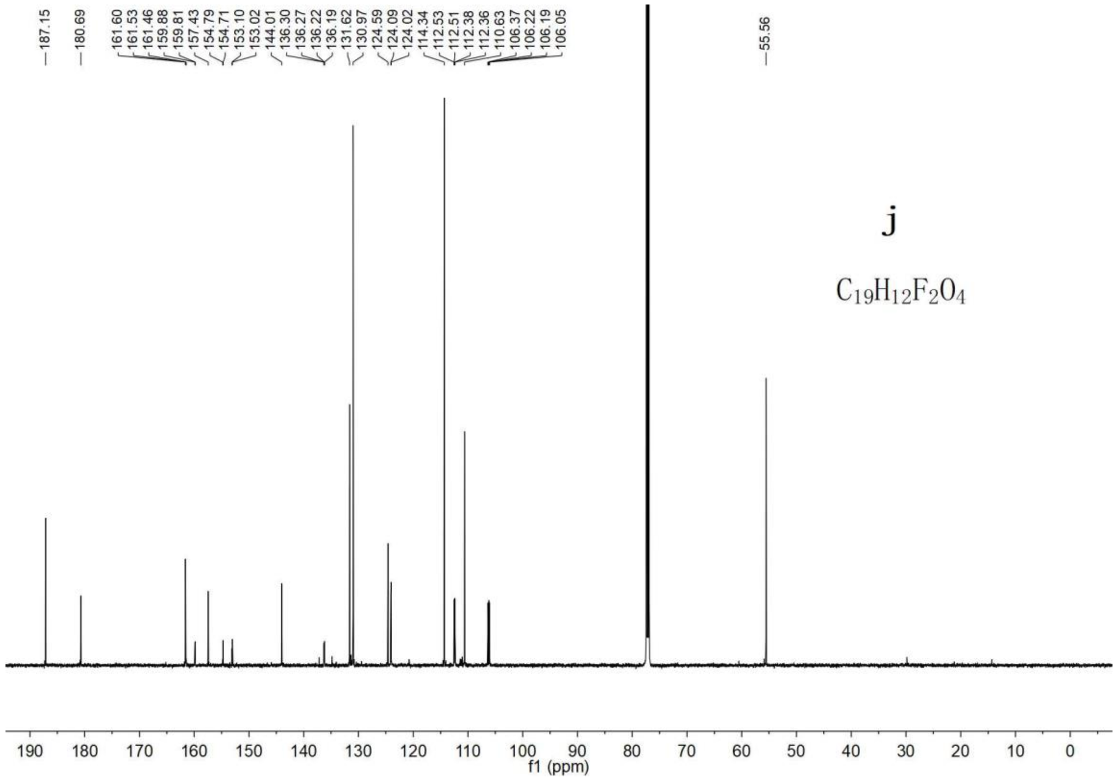

^{19}F NMR (**j**)

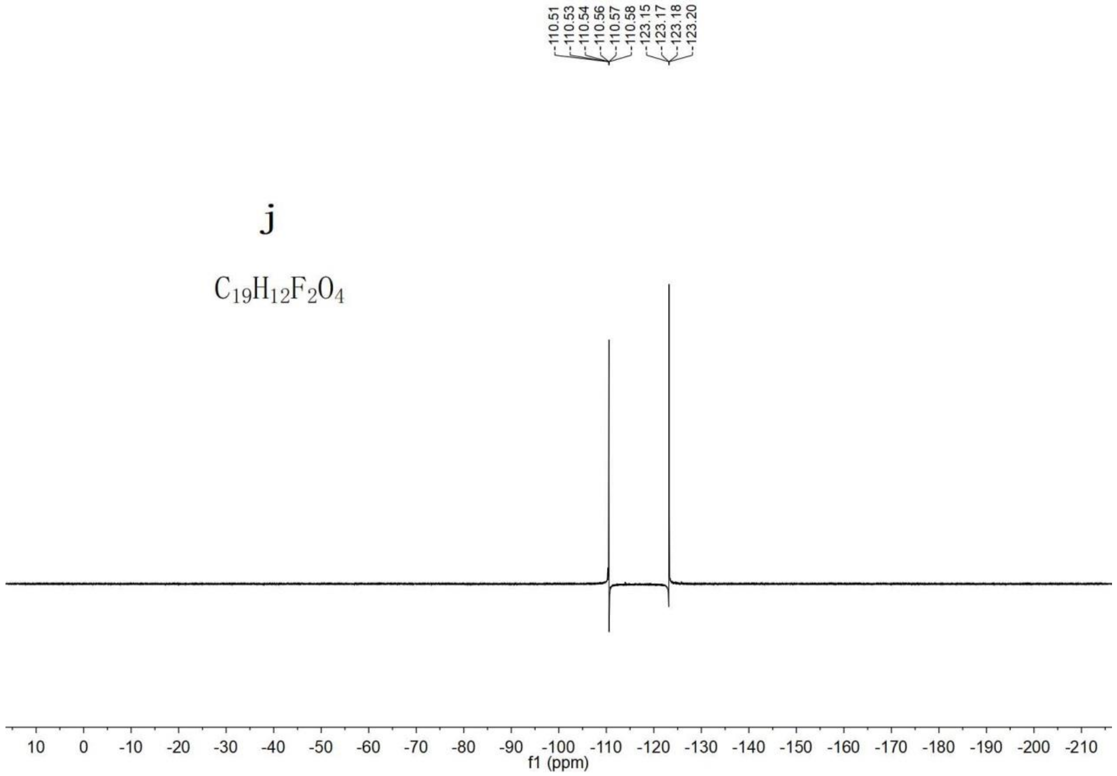

-110.51
-110.53
-110.54
-110.56
-110.57
-110.58
-123.15
-123.17
-123.18
-123.20
j
C19H12F2O4
10 0 -10 -20 -30 -40 -50 -60 -70 -80 -90 -100 -110 -120 -130 -140 -150 -160 -170 -180 -190 -200 -210
f1 (ppm)

NOESY (**6e**)

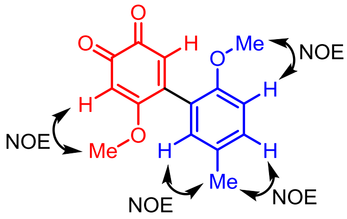

6e

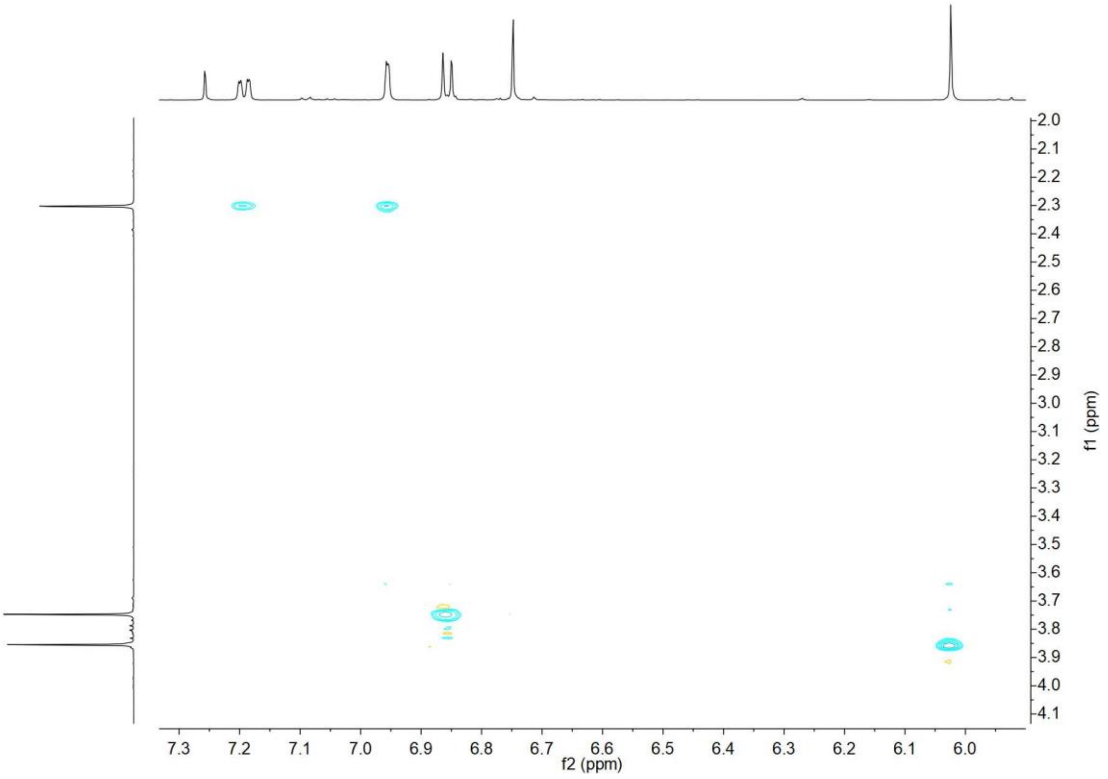

Supplement: Supplementary file 1 — Supplementary [file ANIE-58-18530-s001.pdf]
